# Influence of Tartrate Ligand Coordination over Luminescence Properties of Chiral Lanthanide-Based Metal–Organic Frameworks

**DOI:** 10.3390/nano12223999

**Published:** 2022-11-13

**Authors:** Uxua Huizi-Rayo, Xuban Gastearena, Ana M. Ortuño, Juan M. Cuerva, Antonio Rodríguez-Diéguez, Jose Angel García, Jesus Ugalde, Jose Manuel Seco, Eider San Sebastian, Javier Cepeda

**Affiliations:** 1 Donostia International Physics Center, Paseo Manuel de Lardizabal 4, 20018 Donostia, Spain; 2 Departament of Applied Chemistry, Faculty of Chemistry, University of the Basque Country (UPV/EHU), 20018 Donostia, Spain; 3 Department of Organic Chemistry, UEQ, C/Severo Ochoa s/n, University of Granada, 18071 Granada, Spain; 4 Department of Inorganic Chemistry, UEQ, C/Severo Ochoa s/n, University of Granada, 18071 Granada, Spain; 5 Departament of Physics, Faculty of Science and Technology, University of the Basque Country (UPV/EHU), 48940 Leioa, Spain

**Keywords:** chiral MOFs, lanthanides, photoluminescence properties, layered compounds, luminescent thermometry, charge transfers calculation, circularly polarized light emission

## Abstract

The present work reports on a detailed discussion about the synthesis, characterization, and luminescence properties of three pairs of enantiopure 3D metal–organic frameworks (MOFs) with general formula {[Ln_2_(L/D-tart)_3_(H_2_O)_2_]·3H_2_O}_n_ (**3D_Ln-L/D**, where Ln = Sm(III), Eu(III) or Gd(III), and L/D-tart = L- or D-tartrate), and ten pairs of enantiopure 2D coordination polymers (CPs) with general formula [Ln(L/D-Htart)_2_(OH)(H_2_O)_2_]_n_ (**2D_Ln-L/D**, where Ln = Y(III), Sm(III), Eu(III), Gd(III), Tb(III), Dy(III), Ho(III), Er(III), Tm(III) or Yb(III), and L/D-Htart = hydrogen L- or D-tartrate) based on single-crystal X-ray structures. Enantiopure nature of the samples has been further corroborated by Root Mean Square Deviation (RMSD) as well as by circular dichroism (CD) spectra. Solid-state emission spectra of Eu(III), Tb(III), and Dy(III)-based compounds confirm the occurrence of ligand-to-metal charge transfers in view of the characteristic emissions for these lanthanide ions, and emission decay curves were also recorded to estimate the emission lifetimes for the reported compounds. A complete theoretical study was accomplished to better understand the energy transfers occurring in the Eu-based counterparts, which allows for explaining the different performances of 3D-MOFs and 2D-layered compounds. As inferred from the colorimetric diagrams, emission characteristics of Eu-based 2D CPs depend on the temperature, so their luminescent thermometry has been determined on the basis of a ratiometric analysis between the ligand-centered and Eu-centered emission. Finally, a detailed study of the polarized luminescence intensity emitted by the samples is also accomplished to support the occurrence of chiro-optical activity.

## 1. Introduction

In recent years, metal–organic frameworks (MOFs) have caught the attention of researchers working in the field of multifunctional materials [1,2,3,4] due to their structural and chemical versatility, derived from an infinite number of possible combinations of organic ligands and central metal cations, yielding an equally infinite number of MOFs functionalized as desired [5,6,7]. This endless family of compounds is accompanied by a very wide list of interesting applications [8], derived from their large variety of physicochemical properties. New materials based on MOFs have currently demonstrated applications in the fields of gas adsorption and separation [9,10,11], drug or biomolecule release [12], heterogeneous catalysis [13], ionic conductivity [14,15] and crystallization templates [16,17].

The porosity of MOFs, being both ultrahigh and chemically easy to tailor, is partially responsible for the increasing interest in this family of materials, which can present such a high internal surface area that applications in catalysis and storage become straightforward [18,19]. Moreover, their rational design and tunability at the molecular level have caused MOFs to become adequate materials in the field of photoluminescence (PL)-based sensing [20], since this particular characteristic has allowed the development of luminescent MOFs with widely varied fluorimetric sensing properties following crystal engineering design rules and correlations between their structure and properties [21], which results in PL MOF growing, with large applicability, as enhanced solid-state photodevices (OLEDs, LLPs, and so on) [20,22,23] as well as molecular sensors [24,25]. Notwithstanding the latter, sensing carried out by MOFs is being further extended to other applications such as thermometry, given the interesting behavior observed in the PL of some CPs under variable temperatures [26,27,28].

In the search and development of materials based on MOFs with enhanced PL, the occurrence of those built from lanthanide(III) ions has significantly increased, due to their unique luminescent properties derived from the presence of a shielded 4f electron shell [29,30]. Therefore, lanthanide-based MOFs (LnMOFs) or, generally speaking, lanthanide-based coordination polymers (LnCPs), present luminescence originated from the intraionic *f-f* transitions characterized by very narrow and long-lived emissions in the near-infrared and visible regions of the electromagnetic spectrum, since the shielded f-electrons avoid the influence of the chemical environment of the lanthanide [31,32,33]. The main advantage of LnMOFs is the improvement of the emission by the well-known antenna effect, since the lanthanide coordination to ligands may provoke a highly efficient ligand-to-metal energy transfer, which, in turn, enhances the low absorption coefficients to such Laporte forbidden *f-f* transitions [34,35]. In this sense, and taking into account that metal-ligand bond strength importantly affects such effect, appropriate ligands may contain carboxylate linkers with large coordination capacity [36].

In addition, providing chiral properties to MOFs increments the versatility and robustness of such materials [37] and opens the door to novel applications based on less explored physical phenomena arising from the interplay of the electrons in chiral environments [38]. These properties are proving to be useful and applicable in non-linear optics and magnetic materials [39,40,41]. They are of particular interest in the emission of polarized luminescence, mainly in the form of circularly polarized luminescence (CPL), due to its applications in quantum computation [42,43], optical data storage [44], the Floquet topological state [45,46], as well as in fields such as chirality sensing [47] and more potent contrast agents for advanced medical imaging technologies [48]. However, the low proportion of CPL signal with respect to overall luminescence demands a high value of luminescence dissymmetry ratio (*g_lum_*), defined as *g_lum_* = 2(I*_L_* − I*_R_*)/(I*_L_* + I*_R_*), where I*_L_* and I*_R_* are the intensities of left and right circularly polarized emitted light, respectively. This is a major challenge considering that the mechanisms responsible for the chiroptical response are still unknown [49,50,51]. Although CPL studies are currently dominated by organic molecules and the number of chiral MOFs with demonstrated chiroptical properties is still scarce [52,53], the use of heavy atoms such as lanthanides(III) [54,55] may undoubtedly boost the performance of CPL emitters, as demonstrated in a previous work by our research group [56]. Therefore, it seems reasonable to think that the development of the technology will require systematic studies of more chiral Ln-based luminescent MOFs in order to achieve that goal.

Following our previous report on five pairs of isostructural 3D microporous enantiomeric MOFs based on Ln(III) ions with interesting magnetic and chiral luminescence properties [56], we are now studying the structural features to accomplish a detailed comparison of the resulting properties. In particular, compounds based on 3D and 2D structures were analyzed to explore the effect of the coordination of tartrate ligand on their photoluminescence and chiroptics. We herein report the synthesis and a complete characterization of three isostructural enantiomeric pairs of 3D MOFs, and ten isostructural enantiomeric pairs of 2D MOFs, all based on Ln(III) ions and either D- or L-tartrate ligand, with intriguing luminescent and chiroptical properties.

## 2. Materials and Methods

### 2.1. Synthesis of {[Ln_2_(μ_4_-tar)_2_(μ-tar)(H_2_O)_2_]·xH_2_O}_n_ [Where Ln(III) = Sm, Eu and Gd]

The hydrothermal procedure for the generation of single crystals of all compounds was as reported elsewhere [56,57]. Briefly, 5 mL of an aqueous solution of the corresponding Ln(III) nitrate (0.6 mmol, Merck KGaA, Darmstadt, Germany) was mixed with 5 mL of an aqueous solution of the chiral tartaric acid (L- or D-H_2_tar) (135.1 mg, 0.9 mmol, Merck KGaA, Darmstadt, Germany) and briefly sonicated. The resulting solution was heated for 48 h in a Teflon liner at 160 °C and slowly cooled down to room temperature. Elemental analyses (EA) of the products and additional details on the synthesis procedure can be found in the Supplementary Materials (Sections S5–S9).

### 2.2. Synthesis of [Ln(μ-Htart)_2_(OH)(H_2_O)_2_]_n_ [Where Ln = Y(III), Sm(III), Eu(III), Gd(III), Tb(III), Dy(III), Ho(III), Er(III), Tm(III), or Yb(III)]

The solvent evaporation procedure was used for the generation of single crystals of all compounds. Briefly, 4 mL of an aqueous solution of the corresponding Ln(III) nitrate (0.6 mmol, Merck KGaA, Darmstadt, Germany) was mixed with 4 mL of an aqueous solution of the chiral tartaric acid (L- or D-H_2_tar) (135.1 mg, 0.9 mmol, Merck KGaA, Darmstadt, Germany) and sonicated. The resulting solution was heated in a vial for 24 h at 50 °C. Elemental analyses (EA) of the products and additional details on the synthesis can be found in the Supplementary Materials (Sections S5–S9).

### 2.3. Physical Measurements

Details on the equipment and methods followed for the characterization of the materials by means of EA (Fisons-Carlo Erba analyzer model EA 1108, ThermoScientific, Waltham, MA, USA), FT-infrared spectra (Nicolet FT-IR 6700 spectrometer, Thermo Scientific, Madrid, Spain), metal content (Fisons-Carlo Erba analyzer model EA 1108), thermal analyses (TG/DTG, TGA/DSC 3+, METTLER TOLEDO, Madrid, Spain), and luminescence measurements can be found in S1, S14, S15 sections of the Supplementary Materials.

### 2.4. X-ray Diffraction Data Collection and Structure Determination

X-ray data collections and reductions were acquired on suitable single crystals of 3D and 2D Ln-L/D compounds with Agilent Technologies Super-Nova and Bruker VENTURE diffractometers, using WINGX crystallographic package [58,59] to refine the crystal structures (see Section S2 and Tables S1 and S2 of the Supplementary Materials for further details). The supplementary crystallographic data were deposited with the Cambridge Crystallographic Data Center (CCDC numbers 2157993-2157998). Details on X-ray powder diffraction (PXRD) patterns and variable-temperature PXRD acquisition are also gathered in Sections S10–13 of the Supplementary Materials.

### 2.5. Photophysical and Chiroptical Properties

Photoluminescence measurements were conducted as detailed in the Supplementary Materials (see Sections S16 and S17) and chiroptical measurements were performed as detailed in Section S20 of the Supplementary Materials (Sections S3 and S4).

### 2.6. Computational Details

All Sparkle calculations were carried out using MOPAC2016 (James Stewart, Stewart Computational Chemistry) and all RM1 model for europium calculations were carried out by a modified version of the same software [60]. Calculations were performed either at the crystallographic geometry or by fully optimizing the geometry at the particular level of theory, taking care to ensure the absence of imaginary vibrational frequencies. The Judd–Ofelt intensity parameters were calculated using the Lanthanide Luminescence Software Package (LUMPAC 1.4.1, Pople Computational Chemistry Laboratory, Federal University of Sergipe, Brazil) [61]. 

## 3. Results and Discussion

### 3.1. Comments on the Synthesis of Compounds

Following the procedures described in the Experimental Section, three pairs of enantiopure 3D MOFs of general formula {[Ln_2_(L/D-tart)_3_(H_2_O)_2_]·3H_2_O}_n_ (where Ln = Sm(III), Eu(III) or Gd(III), and L/D-tart = L- or D-tartrate), and ten pairs of enantiopure 2D MOFs of general formula [Ln(L/D-Htart)_2_(OH)(H_2_O)_2_]_n_ (where Ln = Y(III), Sm(III), Eu(III), Gd(III), Tb(III), Dy(III), Ho(III), Er(III), Tm(III), or Yb(III), and L/D-Htart = hydrogen L- or D-tartrate) were synthesized and obtained as single crystals. As all crystal structures confirm, no racemization occurred during the reactions, which is opposite to some previous reports on chiral compounds [62].

Regarding the synthetic procedures, various studies have demonstrated that, in addition to pH, solvent, and molar ratio, reaction conditions such as temperature, pressure, and time are key to determine the resulting MOF architecture [63]. In this way, solvothermal/hydrothermal synthesis, which imply closed systems with high temperatures and pressures (above 100 °C and 1 atm), give rise to thermodynamically favored products of denser, less hydrated, and higher dimensional frameworks with extended M-O-M networks as a consequence of the entropy-driven desolvation effect, by which solvent (water) molecules are hardly coordinated to the metal atom leaving coordination sites for the ligands [64,65]. Instead, conventional synthesis at low or moderate temperatures results in different kinetically favored products, usually of lower dimensionality [66,67]. Accordingly, and as detailed below, hydrothermal synthesis yielded three-dimensional MOFs, whereas solvent evaporation at moderate temperatures gave rise to two-dimensional layered CPs.

### 3.2. Structural Description of {[Ln_2_(μ_4_-tar)_2_(μ-tar)(H_2_O)_2_]·3H_2_O}_n_ [where Ln(III) = Sm, Eu and Gd]

Despite the fact that twinned crystals of these compounds were obtained, due to the high disorder found in the refinements the corresponding structures could not be resolved except for compound **3D_Sm-D**, which was partially solved (further information in the Supplementary Materials). Nevertheless, it was possible to check by PXRD that these MOFs are isostructural to a family of porous compounds with {[Ln_2_(μ_4_-tar)_2_(μ-tar)(H_2_O)_2_]·3H_2_O}_n_ (Ln = Sm, Eu, Gd) formula [56], which consists of a 3D open framework containing microchannels that crystallize in the non-enantiogenic *P*1 space group (see Table 1 and Figure S1 in the Supplementary Materials for further information). In that work, an asymmetric unit composed of two crystallographically independent Ln(III) ions [Ln(1) and Ln(2)], three tartrate ligands, two coordination water molecules, and four lattice water molecules was described. Both Ln(III) atoms are nine coordinated species, with eight oxygen donor atoms from three tartrate dianionic ligands (six carboxylate and two hydroxyl oxygen atoms) and one additional oxygen donor atom provided by a coordinated water molecule [Ln(O_carb_)_6_(O_hid_)_2_(O_w_)_1_]. In most of the structures, tartrate ligands show two different coordination modes: one tartrate displaying the bis(bidentate) *μ-κ^2^O,O’:κ^2^O’’,O’’’* mode, and two tartrate ligands displaying a hexadentate *μ_4_-κO:κ^2^O’,O’’:κ^2^O’’’,O’’’’:κO’’’’’* mode that exerts two five-member chelating rings involving a carboxylate and a hydroxyl oxygen atom. All the bridges imposed by tartrate ligands among neighboring Ln(III) atoms yields a 3D framework that may be described with the fsx topology since it possesses the (4^2^·6^4^)(4^2^·6^7^·8) point symbol, taking into account that Ln(III) and μ_4_-tar ligands act as 5- and 4-connected nodes. The growth of this structure leaves narrow microchannels along the crystallographic [001] direction that correspond to the ca. 13.5% of the unit cell volume and are occupied by crystallization water molecules.

### 3.3. Structural Description of [Ln(μ-Htart)_2_(OH)(H_2_O)_2_]_n_ [where Ln = Y(III), Sm(III), Eu(III), Gd(III), Tb(III), Dy(III), Ho(III), Er(III), Tm(III), or Yb(III)]

As confirmed by X-ray diffraction data, these isostructural compounds consist of a 2D-layered framework that crystallizes in the non-enantiogenic *P4_1_2_1_2* (L-enantiomers) and *P4_3_2_1_2* (D-enantiomers) space groups. Accordingly, both structures retain the chirality afforded by L-/D-tartrate ligands. It should be noted that, unlike the above-described three-dimensional tartrate-based compounds, these MOFs contain hydrogen tartrate ligands, hence with a single deprotonated carboxylate group capable of coordinating with metal centers. As a result, the crystal structure is limited to a two-dimensional layer.

**Figure 1:**
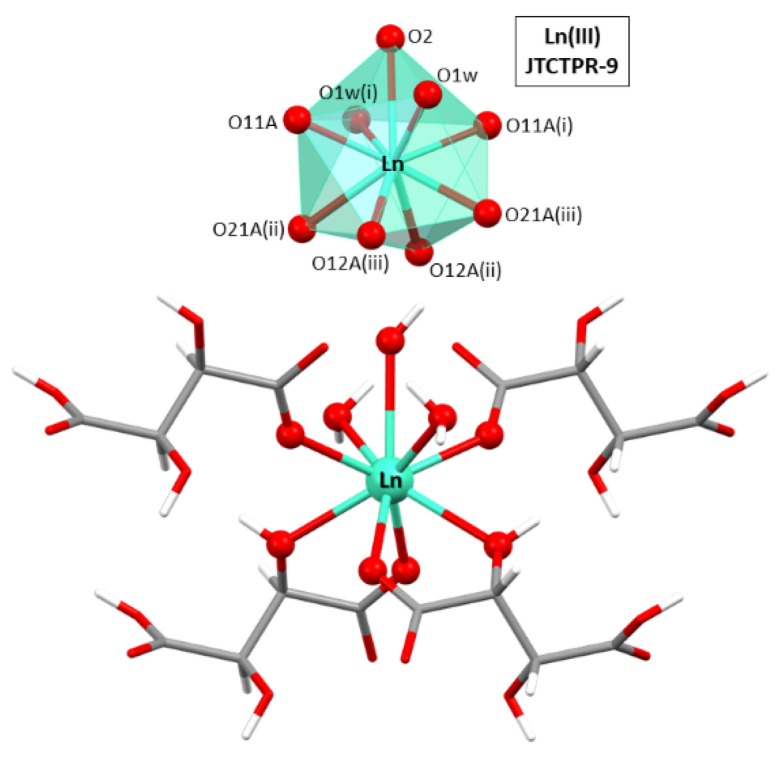
Excerpt of the crystal structure of compound **2D_Gd-L** showing the coordination polyhedron for Ln(III) atoms. Symmetries: (i) −1 + y, 1 + x, 1 − z; (ii) −1 + x, y, z; and (iii) −1 + y, x, 1 − z.

The asymmetric unit is composed by half a Ln(III) ion, one hydrogen tartrate ligand, half a hydroxide anion, and a coordination water molecule. As for the lanthanide atoms, they present a nine-fold coordinated sphere, with six oxygen atoms, four of which belong to two chelating hydrogen tartrate ligands (by means of the carboxylate and neighboring hydroxyl oxygen atoms) and the remaining two to the non-chelating carboxylate oxygen atoms, two oxygen atoms from coordination water molecules, and one additional oxygen donor atom of the hydroxide ion (see Figure 1). It is important to notice that the oxygen atom of the hydroxide group is sited on a binary axis, a special position of the lattice with half multiplicity, in such a way that the hydrogen atom is inherently disordered into two equivalent positions. Continuous shape measurements (CShMs) [68] on the [Ln(O_carb_)_3_(O_hid_)_3_(O_w_)_2_(O_OH_)_1_] chromophore revealed that Ln atoms are surrounded by a tricapped trigonal prism J51 (JTCTPR-9). The hydrogen tartrate ligands display a tridentate µ-*kO:k^2^O’,O’’* coordination mode by which they bridge Ln atoms one another through the five-member chelating ring and the non-chelating monodentate carboxylate oxygen atoms.

**Figure 2:**
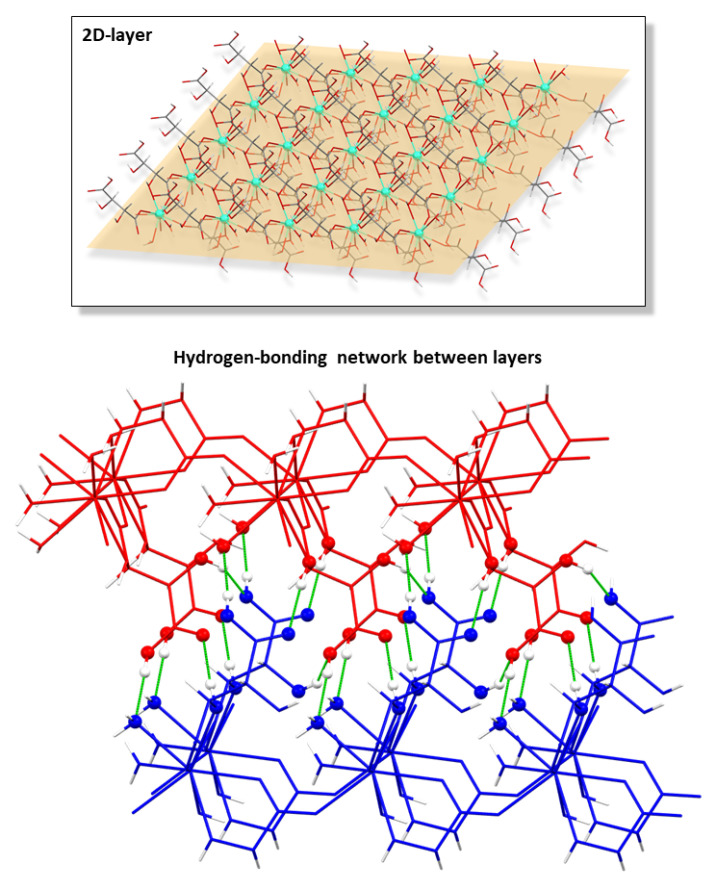
Chiral two-dimensional layer of **2D_Gd-L** MOFs and the hydrogen bonding scheme of interconnected layers.

The successive bridges established between hydrogen tartrate ligands and Ln(III) atoms yield a 2D chiral layer exhibiting a four-membered grid that may be described as a **sql** topological network with the (4^4^·6^2^) point symbol [69]. Another interesting feature of the layers is the fact that the coordinated water molecules and hydroxide anions act as hydrogen bonding donors in some remarkable intramolecular interactions that reinforce the arrangement of the grid (Table 2). From there on, the layers are interconnected with each other by means of a hydrogen-bonding network that involves the hydroxyl and carboxylate groups (Table 3 and Figure 2), which directs the piling of the layers to give rise to the overall compact three-dimensional framework.

Importantly, all our D and L-tartrate based enantiomeric pairs of MOFs have been subjected to an atomic level structural comparison by means of root mean square deviation (RMSD) analysis (see Table S9) showing that D-tartrate-based MOFs show a perfectly equivalent arrangement which results from the application of an inversion center (see Table S3 in Supplementary Materials).

**Figure 3:**
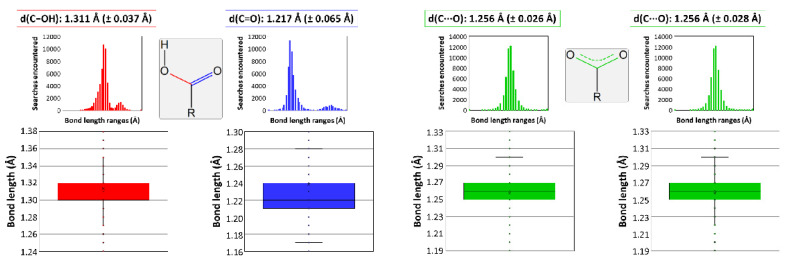
Data analysis on the C-O bond length variation depending on the functional group. Colors correspond to bonds that can be found in a carboxylic group (data in red belong to the C-OH bond and in blue to the C=O bond), and in a carboxylate group coordinated to a metal (describing with green color the data corresponding to the C···O bond).

It must be mentioned that other works gathered in the bibliography report some two-dimensional CPs which, despite being very similar, cannot be considered isostructural to those studied in this paper. The main difference lies on the protonation of tartrate because those compounds consist of a dianionic tartrate, a hydrogen tartrate, and three water molecules according to the [Ln(μ-Htart)(μ-tart)(H_2_O)_3_]_n_ formula, which excludes the presence of a hydroxide anion as in the herein described compounds [70,71,72,73,74]. Notwithstanding the fact that hydroxide ions are easily formed in the presence of oxophilic lanthanides(III) and usually incorporated to neutral and stable two-dimensional LnMOFs [75], bond distances of carboxylate groups were analyzed and compared with the results deposited in the CCDC database to support the occurrence of a unique type of tartrate ligand in the latter [76]. In essence, C-O bonds of the terminal carboxylate group are 1.282 Å, in line with single C-O bonds, and 1.228 Å, corresponding to a double C=O bond (Figure 3), whereas they are similar to each other and close to the media found for deprotonated carboxylates in those reported compounds.

### 3.4. Thermal Evolution of the 3D and 2D Compounds

As reported in our previous work [56], the thermal behavior of the 3D MOFs entails an interesting feature because it involves several crystalline transformations accompanying the progressive dehydration of the material, and three-dimensional enantiomeric pairs of Sm, Eu, and Gd are no exception (see Supplementary Materials for further information).

**Figure 4:**
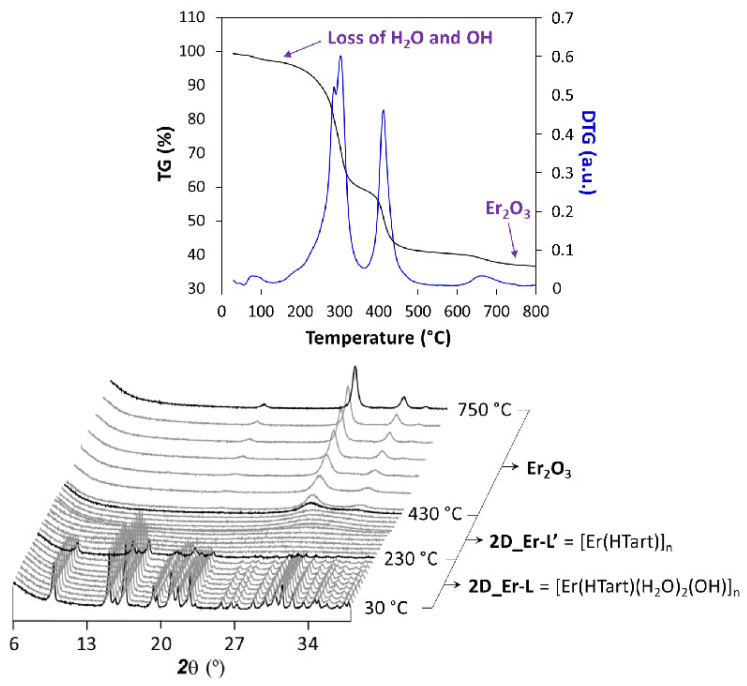
Analysis of the thermal behavior of compound **2D_Er-L**. Note: diffractograms shown in black correspond to temperatures at which phase transition takes place.

On another level, the thermal behavior of these 2D MOFs entails an interesting stability derived from the strong interlamellar hydrogen bonds occurring in the framework. TG analysis showed in Figures S6–S15 in the Supplementary Materials confirm a common temperature-dependent behavior of all compounds; therefore, **2D_Er-L** was selected as a representative sample to conduct a thermodiffractometric study. Compound **2D_Er-L** remains stable and crystalline in the 30–230 °C range (Figure 4), a temperature range where the loss of coordinated water and hydroxide molecules takes place. Upon heating, the sample loses the crystallinity, meaning that the loss of the latter involves the decomposition of the compound to eventually give Er_2_O_3_ as a residue at above 450 °C. Although no further information can be extracted from this study, it may be expected that the amorphous compound obtained in the 230–250 °C range consists of a 3D structure resulting from the junction of the layers, in line with the shortening of the lattice parameters observed during heating.

### 3.5. Luminescence Properties

**Figure 5:**
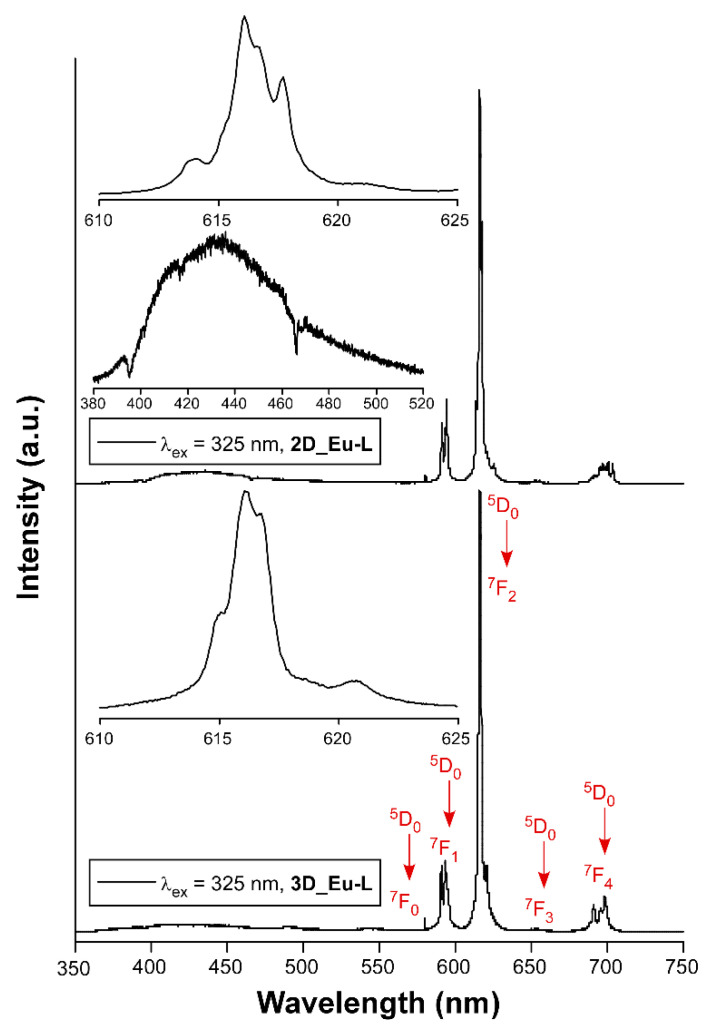
Emission spectra of Eu-based compounds recorded at room temperature showing the assignations and captures of the main intraionic transitions. Insets show augmented regions corresponding to the hypersensitive ^7^F_2_ ← ^5^D_0_ transition and the ligand-based emission.

Lanthanide-centered emissions in CPs are useful in the development of solid-state photodevices [26,77,78], particularly due to their intense emissions either in the visible or the near-infrared (NIR) region of the electromagnetic spectrum [79,80]. For this reason, a thorough analysis of the photoluminescence properties was carried out on polycrystalline samples of L-enantiomeric compounds as representative of the enantiomeric pairs of all compounds (Figures S46–S70 in Supplementary Materials). Starting with the 3D MOFs, the exposure of **3D_Sm-L** and **3D_Gd-L** to UV excitation at room temperature (λ_ex_ = 325 nm of a Xe discharge lamp) yields no characteristic Sm(III) or Gd(III)-centered emissions, but the emission spectra consist of a weak wideband, featured with some intense peaks, covering the 400–650 nm range (see Figures S48 and S49). This band can be attributed to the n ← π* emissions of the tartrate ligands coordinated to lanthanide(III) ions, in good agreement with previous TD-DFT calculations performed over the ligand [56]. On another level, tartrate ligands are able to sensitize europium(III) ions because both 3D and 2D compounds (**3D_Eu-L** and **2D_Eu-L**) show the characteristic emissions of the lanthanide when they are excited under the same previously mentioned experimental setup. Both compounds present emission spectra composed of a first wideband peaking at ca. 400 nm that resembles that shown by compounds **3D_Sm-L** and **3D_Gd-L**, thus assigned to the ligand fluorescence, in addition to intraionic transitions associated with the Eu(III) ion. In particular, a shoulder at 580 nm (^7^F_0_ ← ^5^D_0_), three main bands at 590 nm (^7^F_1_ ← ^5^D_0_), 615 nm (^7^F_2_ ← ^5^D_0_), and 698 nm (^7^F_4_ ← ^5^D_0_), in addition to a minor band sited at 653 nm (^7^F_3_ ← ^5^D_0_), are observed in both cases (Figures S46 and S51). These figures also gather the excitation spectra recorded at the main emission line (λ_em_ = 615 nm), which reveal the absence of any significant wideband and, hence, weak ligand-centered excitation. Instead, the excitation spectra are characterized by narrow bands associated with the intraionic *f-f* transitions of Eu(III), among which ^7^F_0_ → ^5^L_6_ (λ_ex_ = 397 nm) is the most intense one [81]. As a consequence, these compounds present no excitation wavelength-dependent emission as corroborated for **3D_Eu-L** (Figure S47). In view of the good emissive characteristics of the Eu-based MOFs, the samples were then exposed to monochromatic laser excitation (λ_ex_ = 325 nm) under vacuum and at variable temperature. As observed in Figure 5, the spectra are characterized by intense multiplets presenting substantial structure (see the captures of the hypersensitive ^7^F_2_ ← ^5^D_0_ transition centered at 616 nm), which are significantly stronger than the band corresponding to the ligand (λ_em,max_ = 430 nm).

It is known that the ligand scaffold is crucial to modulate the luminescence of a CP since the rigidity of the crystal structure reduces molecular vibrations, rotations, and torsions in the ligand, preventing the non-radiative excitation decay [82]. In this way, changes in the dimensionality of MOFs could also influence the luminescence properties since the structure’s rigidity may affect luminescent processes. For comparative purposes, the relative intensity of the latter is more pronounced for **2D_Eu-L** vs. **3D_Eu-L**, a fact that may indicate a weaker energy transfer from tartrate ligand to Eu(III) for the former. To further analyze the emissive properties, the decay curves were recorded by monitoring the band of the hypersensitive transition using pulsed UV light (λ_ex_ = 325 nm). The curves show a linear exponential shape that suggests the emission of a unique radiative component, so they were fitted with the [I_t_ = A_0_ + A_1_exp(t/*τ*_1_)] equation giving lifetimes of 342.0(6) and 409.1(2) µs, respectively, for **2D_Eu-L** and **3D_Eu-L**. These results, which are similar to other previously reported CPs based on nine-coordinated Eu environments [83,84], are in line with the occurrence of a symmetrically unique Eu(III) ion in **2D_Eu-L** and the fact that the two Eu1 and Eu2 independent ions are practically undistinguishable regarding the coordination environment. Moreover, the shorter lifetime of **2D_Eu-L** compared with **3D_Eu-L** may be attributed to the presence of more coordination water molecules and/or hydroxide anions (3 vs. 1 per Eu(III) ion), which are known to act as effective vibrational quenchers by means of the coupling of O–H oscillators with the energy gaps between the intraionic emissive levels of Eu(III) [85,86]. Moreover, the decay curve collected λ_em_ = 430 nm supports that the process occurring in the ligand is, as expected, short fluorescence of only ca. 1.6 ns thus associated with the S_0_ ← S_1_ (Sections S18 and S19, Figure S67).

**Figure 6:**
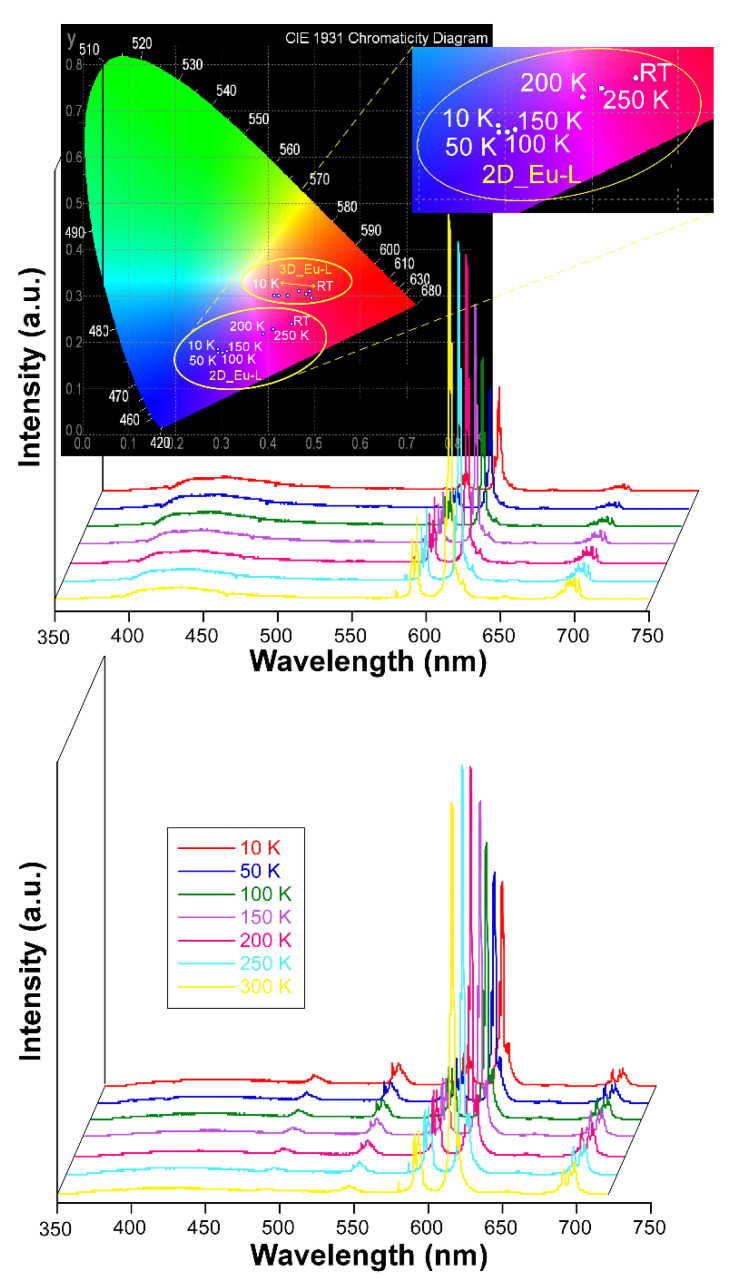
Variable temperature emission spectra of compounds **2D_Eu-L** and **3D_Eu-L** recorded under λ_ex_ = 325 nm.

On another level, when the solid samples are cooled down to 10 K they exhibit an unusual behavior because they do not follow the usual trend by which lanthanide-centered emissions are progressively strengthened when the molecular vibrations are frozen, i.e., vibrational energy of the bonds (Figure S52) [87]. Instead, in this case it was observed that the europium(III) characteristic emissions become less and less intense as the temperature decreases while the ligand-centered emission (band at λ_em,max_ = 430 nm) is maintained all over the inspected temperature range (300—10 K, Figure 6). As a consequence of the progressive change in the relative intensity of the bands, the emitted color of the samples shifts accordingly from red (at RT) towards blue as the ligand fluorescence gains importance. In fact, as inferred from the colorimetric diagrams, the color change is more pronounced for **2D_Eu-L** since it shifts from pinkish red (0.45236, 0.24047) to reddish-purple (0.29445,0.18077) in CIE1931 scale. This interesting behavior seems to be derived from the changing excitation scenario found for the compound around 325 nm with the temperature. In this line, as observed in Figure S52, at 10 K the sample exhibits a unique multiplet centered at 320 nm, whereas at RT it exhibits additional bands covering the 315–330 nm range, which explains the better Eu-centered emission at high temperature. To better characterize the potential performance of **2D_Eu-L** as a luminescent thermometer, the generalized relative sensitivity (*S_r_*) was estimated by means of a ratiometric analysis of the thermal evolution of the relative intensity of the bands attributed to the ^7^F_2_ ← ^5^D_0_ and S_0_ ← S_1_ transitions (see Figure S53). The maximum sensitivity was observed at 50 K (*S_m_* = 1.42 %K^−1^), which is a value that falls within the range found for other lanthanide(III)-based CPs [26,88]. Therefore, although **2D_Eu-L** presents sizeable thermometric luminescence, the changes with temperature are not so large as to further consider this compound as a luminescent sensor of the temperature. Although no further studies have been conducted in this regard, it is worth highlighting that the region of 380–390 nm could be even more adequate as to explore the luminescent thermometry caused by the mentioned temperature-dependent excitation. Interestingly, these variations in the emission hardly impact on the lifetimes as the radiative features of the ^5^D_0_ state remain almost unchanged with a τ of 349.1(1) µs (vs 342.0(6) µs at RT). In contrast, the lifetime is more significantly enlarged for **3D_Eu-L** (from 409.1(2) up to 434.3(2) µs, see Figure S68 in the Supplementary Materials). Moreover, the absolute quantum yields (QY) were measured in solid polycrystalline samples at room temperature by means of an integrating sphere, using the same excitation and emission conditions as for the estimation of lifetimes. Among them, the QY was much higher for compound **3D_Eu-L** (Φ = 32.1%) than for **2D_Eu-L** (Φ = 4.8%). Based on these results, and considering that Φ_Ln_ = τ_obs_/τ_R_, the experimental radiative (*k_r_*) and non-radiative (*k_nr_*) constants can be easily calculated: *k_r_* = 785 s^−1^ and *k_nr_* = 1660 s^−1^ for **3D_Eu-L** and *k_r_* = 140 s^−1^ and *k_nr_* = 2784 s^−1^ for **2D_Eu-L** (Table 4).

In order to better understand the luminescence properties of these two related compounds, the most relevant theoretical parameters were calculated on the basis of the experimentally recorded spectra by means of the LUMPAC program [61]. In this way, we followed a largely contrasted procedure to calculate the intensity parameters and quantum efficiencies as previously discussed for other works [89,90,91,92]. First, appropriate models of the compounds (models 2D-Eu and 3D-Eu hereafter) based on the spherical atomic coordinates of the coordination polyhedra were optimized by the Sparkle/RM1 model, after which charge factor (*g*) and polarizability (*α*) were adjusted according to the experimental emission spectra (Table 5). Fitting of the data by LUMPAC gave 1.51 10^−20^ cm^−1^ and 6.14 10^−20^ cm^−1^ (Ω_2_), 0.18 10^−20^ cm^−1^ and 2.19 10^−20^ cm^−1^ (Ω_4_), and 0.01 10^−20^ cm^−1^ and 0.07 10^−20^ cm^−1^ (Ω_2_), respectively, for **2D_Eu-L** and **3D_Eu-L**. From these values, the intensity parameters were estimated as follows: *A_rad_* equals 149.7 s^−1^ for **2D_Eu-L**, with a contribution of the magnetic transition (^7^F_1_ ← ^5^D_0_) being 90.2 s^−1^, whereas the value of *A_rad_* increases up to 827.8 s^−1^ with a magnetic contribution of only 16.3 s^−1^ for **3D_Eu-L**. Taking into account the experimental lifetimes recorded for both compounds at RT, the non-radiative rates (*A_nrad_*) may be estimated to be of 2774.2 and 1616.6 s^−1^, which corroborates the better performance shown by **3D_Eu-L**. These values are slightly smaller than those experimentally estimated; however they are in the range of the results commonly observed for other luminescent complexes using the same computational methodology (see Table 4) [90,93].

**Figure 7:**
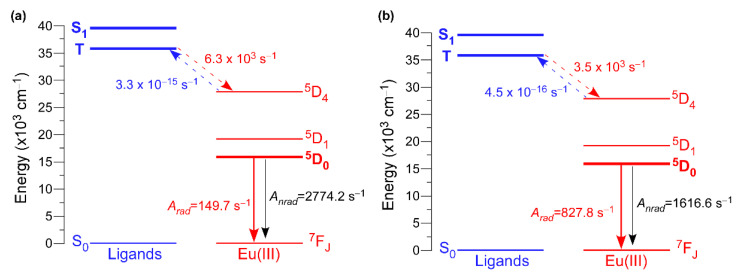
Schematic diagram of the main states and transfer rates involved in the luminescence of compounds: (**a**) **2D_Eu-L** and (**b**) **3D_Eu-L**.

Another relevant parameter to be determined to gain deeper insight into the energy transfers occurring in these compounds is the energy of the ligand’s excited states. To that end, the configuration interaction simple (CIS) of INDO/S implemented into ORCA program was employed [94,95]. These calculations set the singlet (S) and triplet (T) excited states around 39,000 (39304 and 38,997 cm^−1^ for **2D_Eu-L** and **3D_Eu-L**) and 36,500 cm^−1^ (36,360 and 36,653 cm^−1^ for **2D_Eu-L** and **3D_Eu-L**). The non-radiative energy transfer rates between the ligands’ and Eu(III) excited states were also calculated by means of Malta’s models [96], which consider the occurrence of three mechanisms for the excitation of metal ions during the antenna effect: dipole-2^λ^pole, dipole–dipole, and exchange. A comparative analysis for both compounds brings, once again, another important difference between them because both singlet (S) → ^5^D_4_ and triplet (T) → ^5^D_4_ multipolar transfers (*W_ET_* being 4.44 10^2^ and 3.45 10^3^ s^−1^, respectively) are dominant for **3D_Eu-L**, whereas only the T → ^5^D_4_ is significant (*W_ET_* = 6.26 10^3^ s^−1^) for **2D_Eu-L** (Figure 7 and Table 6). The lower values found for the T → ^5^D_1,0_ transitions suggest the lesser importance of the exchange mechanism in both compounds. Similarly, the back-transfer rates are slightly greater for **2D_Eu-L** than for **3D_Eu-L**, among which the triplet ← ^5^D_4_ is the dominant with values of *W^B^_ET_* = 3.32 10^−15^ and 4.49 10^−16^ s^−1^. Using all these data, the quantum efficiency is determined as 5.12 and 33.87% for **2D_Eu-L** and **3D_Eu-L**, respectively, which are comparatively higher than the experimental values (see Table 6). All these data are in line with the previously mentioned ratio of H_2_O/OH per Eu(III), which is 3 for **2D_Eu-L** but only 1 for **3D_Eu-L**, in such a way that the probability of the O–H oscillator-driven quenching [97], mediated through a vibronic coupling with the Eu-centered excited states, is simply higher. Therefore, this fact could be responsible for the large non-radiative contribution and, hence, low emission efficiency present in compound **2D_Eu-L**.

**Figure 8:**
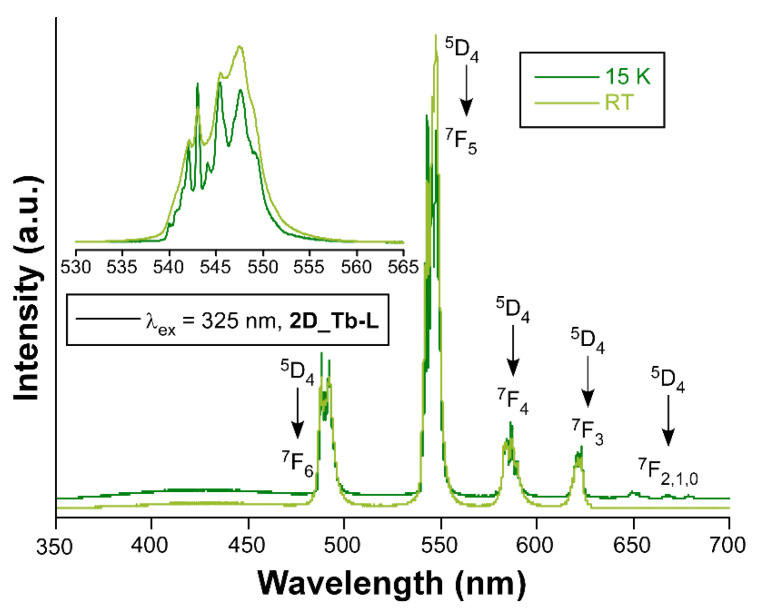
Emission spectra of compounds **2D_Tb-L** recorded at RT and 15K.

When Tb(III) ion occupies the crystallographically independent metal site of the two-dimensional network, the solid sample of compound **2D_Tb-L** displays bright green emission upon irradiation with UV light. Although it is true that the emission spectrum at RT with a Xe discharge lamp (λ_ex_ = 325 nm) presents both the characteristic intraionic bands and the band assigned to ligand’s fluorescence, the latter is not the dominating one in contrast with previous compounds. In fact, the spectrum collected under monochromatic laser beam at the same wavelength shows only the four groups of signals sited at 490 nm (^7^F_6_ ← ^5^D_4_), 544 nm (^7^F_5_ ← ^5^D_4_), 585 nm (^7^F_4_ ← ^5^D_4_), and 622 nm (^7^F_3_ ← ^5^D_4_) arising from being centered on Tb(III) ions. Under variable temperature, this compound also exhibits sizeable color change as depicted in Figure 8, which is thought to come from the relative increase in the bands assigned to the intraionic excitations of the lanthanide ion (see Figure S54). The analysis of the decay curves reveals that the radiative emission of Tb(III) in this structure rises up to 870.1(4) µs at RT, while it is 894.9(5) µs at 10 K (see Figure S69 in the Supplementary Materials). The QY for this sample at RT was also experimentally measured (Φ = 28%).

The analysis of the luminescent properties of **2D_Dy-L** shows that this compound presents a similar behavior with respect to its counterparts. The emission spectrum under monochromated laser excitation (λ_ex_ = 325 nm) shows two characteristic bands at 481 nm (^6^H_15/2_ ← ^4^F_9/2_) and 574 nm (^6^H_13/2_ ← ^4^F_9/2_, Figure S56) [81]. It is remarkable that the band assigned to the tartrate ligand (λ_em_ = 413 nm) remains comparatively weak with the latter bands despite the fact that tartrate lacks strong absorbing chromophores, a fact that derives from the matching of the employed excitation wavelength with the dysprosium’s intraionic transitions (see Figure S55). Given that these spectra do not change with the temperature, **2D_Dy-L** hardly changes the emission with regard to the temperature, except for the usual increase in emission intensity with the drop in temperature that implies no remarkable color change (Figure S57). However, further analysis of the radiative signal by means of the emission lifetimes on the solid indicates the sensitization gets worse when lowering the temperature, since the ligand’s fluorescence doubles its lifetime (λ_em_ = 413 nm is 1.4 and 2.4 ns at RT and 10 K, respectively, see Figure S70) whereas Dy(III)’s emission is slightly shortened (λ_em_ = 574 nm is 23.2 and 21.2 µs at RT and 10 K, respectively, see Figure S71). A very low QY was also estimated from the experimental measurement (Φ = 1.6%).

At last, compounds **2D_Y-L, Sm-L, 2D_Gd-L, 2D_Ho-L, 2D_Er-L, 2D_Tm-L,** and **2D_Yb-L** yielded no characteristic lanthanide(III)-centered emissions upon exposition to UV excitation but only an almost identical band in the 400–650 nm range (see Figures S57–S63) corresponding to the n ← π* emissions of the metal-coordinated hydrogen tartrate ligands.

### 3.6. Circular Dichroism (CD) Experiments

**Figure 9:**
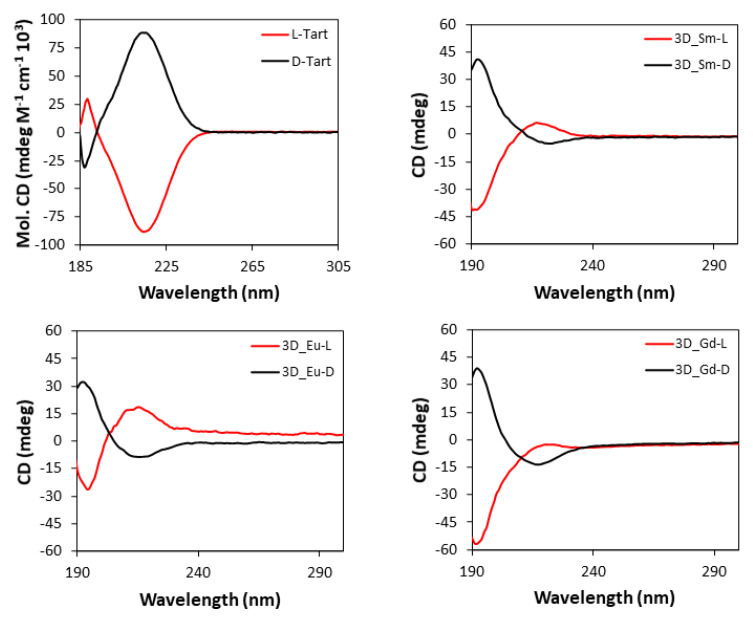
CD spectra of pure L- and D-tartaric acid and **3D_Ln** compounds.

Based on the chiral character of the prepared MOFs, their differential capacity to absorb circularly polarized light was explored acquiring CD (and the corresponding UV-Vis, Section S21) spectra for water suspensions of each pair of enantiomeric MOFs, as well as for the water solutions of L- and D- tartaric acid samples. Figure 9 compiles CD spectra of the 3D MOFs, which are characterized by an intense band centered at 195 nm and a less intense one at 221 nm, which are also present in the UV-Vis spectra. As observed, each enantiomeric pair describes mirror curves to each other with opposite Cotton effects, which interestingly present the opposed Cotton effect compared with the free ligand. There are various examples in the literature reporting that optical absorptions of enantiopure materials containing metal-coordinated tartrate ligands show Cotton effects which are opposite in sign to those of the free tartrate ligand spectrum, both in solution and solid state [98,99,100]. For instance, Zhou et al. [101] reported very recently that tartrate ligand coordination to molybdenum generates conformational differences in the ligand that are potentially responsible for the inversion of signal signs.

**Figure 10:**
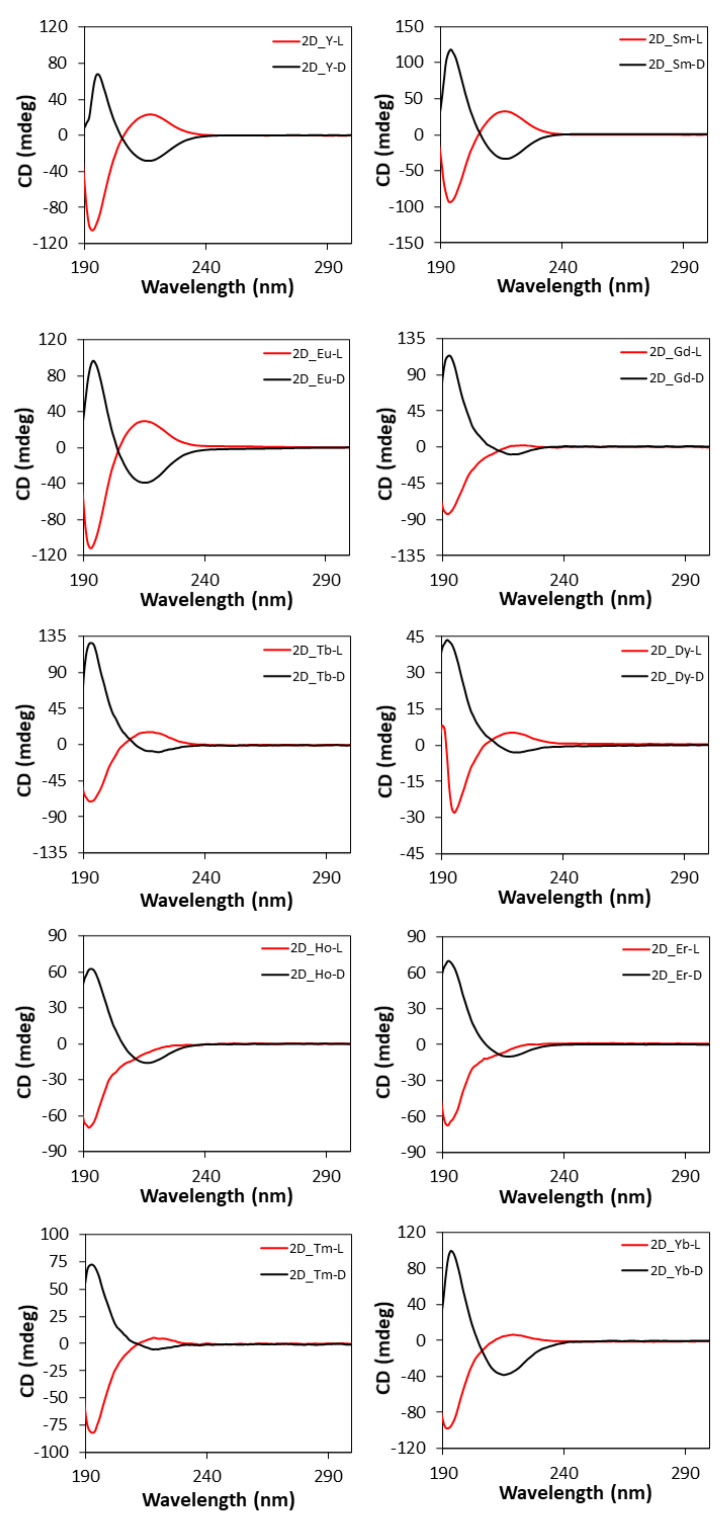
CD spectra of 2D compounds.

The CD spectra of two-dimensional MOFs (Figure 10) show a similar shape to those of 3D compounds since both the intense band at 195 nm and the weak band at 221 nm are present, whereas they retain the same Cotton effects shown by the free ligand, and thus, the opposite behavior to the 3D MOFs [102].

### 3.7. Polarized Luminescence Experiments

**Figure 11:**
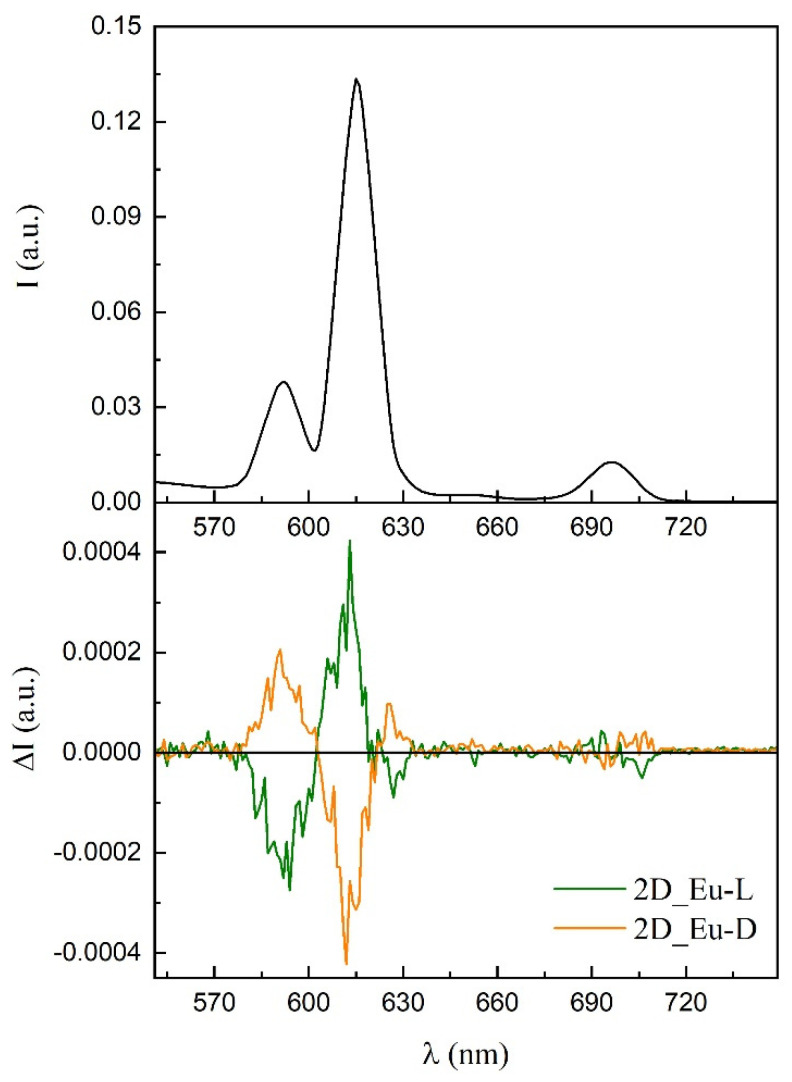
CPL emission plot of the enantiomeric pair of compounds **2D_Eu**.

Persuaded by the capacity of these chiral compounds to both interact with polarized light and emit luminescence in the visible spectra, we decided to study their ability to generate polarized light. As a first approach, samples were evaluated for their potential generation of CPL by analyzing their luminescence in dispersed aqueous solutions, as it was already measured for the terbium(III) counterpart (compound **3D_Tb-L**) [56]. Although several measurements were attempted, the global luminescence was so weak that CPL signal was not reliable. Fortunately, better results were obtained using **2D_Eu-L** and **2D_Eu-D** dispersed in a potassium bromide pill. Both solid dispersions showed mirror data through the whole emission spectra (Figure S74). Different signs can be observed in different bands and even poorly resolved transitions in the same band owing to non-degenerated energetically closed final states. The following $\left|g_{lum}\right|$ values were estimated at 592 (4.5 × 10^−3^), 613 (2.6 × 10^−3^), and 627 (2.9 × 10^−3^) nm. An additional and less intense CPL emission band at 700 nm in the range of the detection limit of the equipment is also observed, although their $g_{lum}$ values cannot be calculated (Figure 11). **2D_Tb-L** and **2D_Tb-D** could be also measured in a similar way.

**Figure 12:**
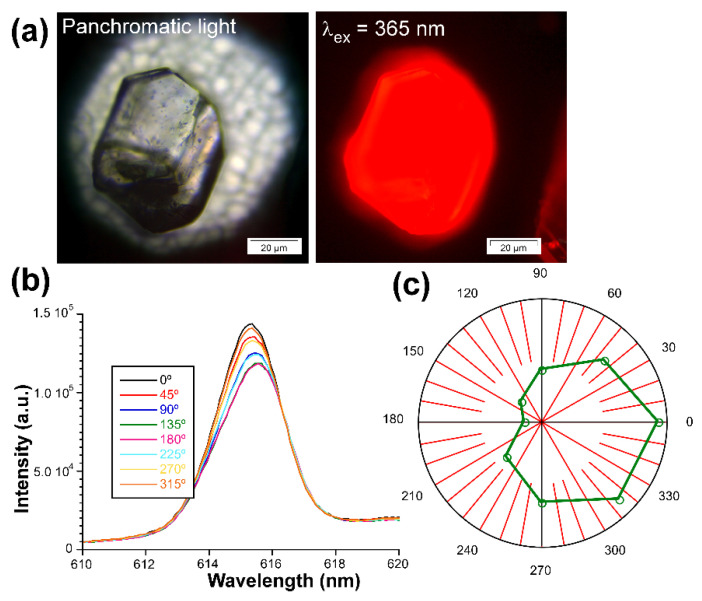
Measurements of the activity of **3D_Eu-L** with polarized light: (**a**) optical image of the crystal illuminated with UV light, (**b**) emission spectra of the ^7^F_2_ ← ^5^D_0_ transition, and (**c**) plot showing the intensity vs. *θ*.

On the other hand, motivated by the good overall emissive properties of **3D_Eu** enantiomers, even in the form of big crystals (see Figure 12), and the ordered disposition of the Eu(III) ions in their chiral structure, the light polarization capacity of these solids was analyzed by measuring the emission spectra acquired on a single crystal according to the variable polarization angle. In a similar experiment carried out by Zhao, Yan, and coauthors [103], a crystal of **3D_Eu-L** was excited on the microscope with polarized UV light (λ_ex_ = 365 nm) and the emitted light of the dominant band (λ_em_ = 616 nm, ^7^F_2_ ← ^5^D_0_ transition) was analyzed on a polarizer by rotating the polarization angle (*θ*). In this setup, *θ* stands for the angle between two polarizers sited along the light path in the microscope before detector. The samples start from the maximum photoemission intensity at 0° (where both incident and emitted light match with each other) so it is slightly decreased as *θ* increases, reaching the minimum emission at 180°. Raising the *θ* further brings a specular behavior by which the intensity is slowly increased to 360°. The emission dichroic ratio calculated as *R_d_* = *I*_0_*/I*_180_ gives 1.24, a value in line with a weak CPL signal and similar to other examples described in the bibliography. In any case, these measurements corroborate the existence of a non-negligible capacity of light polarization by these compounds.

## 4. Conclusions

Two families of enantiomerically pure MOFs, based on Ln(III) cations and either L- or D-tartrate ligands, were synthesized, structurally determined, and characterized. In this system, the temperature of aqueous reaction mixtures is adequately set in order to obtain compounds with either a 2D or a 3D arrangement. In this sense, under mild conditions (below 100 °C), layered **2D_Ln** structures ([Ln(μ-Htart)_2_(OH)(H_2_O)_2_]_n_) are obtained, whereas a hydrothermal procedure yields microporous **3D_Ln** MOFs ({[Ln_2_(μ_4_-tar)_2_(μ-tar)(H_2_O)_2_]·3H_2_O}_n_). RMSD calculations confirm the perfect mirror-like character of the enantiomers of both compounds. Although both compounds share nine-fold LnO_9_ polyhedra, the occurrence of a partially protonated tartrate ligand (associated to the coordination of a hydroxide ion as confirmed by both structural analysis and FTIR spectroscopy) in the former limits its coordination capacity neglecting the formation of carboxylate/hydroxyl chelating rings and, subsequently, decreases the dimensionality of the coordination polymer. An analysis by means of thermodiffractometry reveals that no 2D → 3D transformation in solid state may occur by thermal treatment of the former despite their related structure. All compounds show sizeable photoluminescence under UV excitation, whereas only those compounds containing Eu(III), Tb(III), and Dy(III) ions provide characteristic emissions assigned to intraionic transitions. Eu-based compounds display nice red-colored emissions with relatively long-lived signals. The larger lifetime achieved for **3D_Eu** than **2D_Eu** is attributed to the presence of less O–H oscillators (related to the vibrational quenching) in the former. A detailed study by means of LUMPAC software on the experimental data confirms the hypothesis on the basis of the estimated non-radiative ratios, which are substantially larger for compound **2D_Eu**. Interestingly, the less common evolution of the excitation lines (which are shifted with the temperature) and weak energy transfers occurring in the latter are responsible for a variable-color luminescence thermometry that oscillates between the red emission at RT to the blue glance shown at low temperature according to the CIE1931 pattern. This behavior, comparatively negligible for **3D_Eu**, points to the structural flexibility (characteristic of the layered structure of **2D_Eu**) as the main responsible parameter.

On the other hand, CD experiments measured for solids dispersed in aqueous media confirm the enantiomeric purity of all compounds. In particular, it is worth noting the opposed Cotton effects present for **2D_Eu** and **3D_Eu** because they show converse positive and negative patterns for left- and right-handed enantiomeric frameworks, thus indicating that the Cotton effect resulting from a structure cannot be estimated from isolated molecules. Despite the weak CPL signals observed for most compounds, **2D_Eu** is shown to present $\left|g_{lum}\right|$ value estimates at 592 (4.5 × 10^−3^), 613 (2.6 × 10^−3^), and 627 (2.9 × 10^−3^) nm. Moreover, a detailed study of the polarized luminescence intensity emitted by a single crystal along different orientations confirms the occurrence of an interference between the absorbed light and the chiral structure.

## Figures and Tables

**Table 1:** Crystallographic data of 3D compounds.

Parameters	3D_Sm-L	3D_Sm-D	3D_Eu-L	3D_Eu-D	3D_Gd-L	3D_Gd-D
Crystal System	Triclinic	Triclinic	Triclinic	Triclinic	Triclinic	Triclinic
Space group	*P*1	*P*1	*P*1	*P*1	*P*1	*P*1
*a* (Å)	6.03715	6.03242	6.09457	6.02532	6.01264	6.01300
b (Å)	7.48102	7.47384	7.46433	7.45733	7.44523	7.44541
C (Å)	13.27383	13.25745	13.28264	13.25675	13.23188	13.23119
α (°)	102.69998	102.65131	102.86695	102.73313	102.75539	102.78816
β (°)	101.36651	101.38302	101.99059	101.43393	101.42113	101.42812
γ (°)	90.77908	90.84119	89.85748	90.81119	90.84535	90.84964
V (Å^3^)	572.345	570.685	575.620	568.439	565.214	565.136

**Table 2:** Intramolecular hydrogen bonding interactions (Å, °) of 2D layers ^1^.

D-H⋯A ^2^	D-H	H⋯A	D⋯A	D–H⋯A
O1W-H11W⋯O12A	0.87	1.87	2.694 (4)	156.7
O1W-H12W⋯O31A (i)	0.86	1.91	2.751	166.1
O2-H2⋯O31A (ii)	0.88	2.08	2.913 (6)	158.5

^1^ Symmetry codes: (i) y, x, −z + 1; (ii) x, y + 1, z. ^2^ D: donor. A: acceptor.

**Table 3:** Hydrogen bonding interactions (Å, °) of interconnected 2D layers ^1^.

D-H⋯A ^2^	D-H	H⋯A	D⋯A	D–H⋯A
O21A-H21A⋯O42A (i)	0.85	1.81	2.652(4)	175.1
O31A-H31A⋯O41A (ii)	0.90	2.01	2.828(5)	149.9
O41A-H41A⋯O1W (iii)	0.85	1.80	2.627(5)	163.5

^1^ Symmetry codes: (i) 3/2 − x, 1/2 + y, 5/4 − z; (ii) 1/2 − x, −1/2 + y, 5/4 − z; and (iii) 3/2 − y, 1/2 + x, 1/4 + z. ^2^ D: donor. A: acceptor.

**Table 4:** Summary of experimental and calculated emission parameters for compounds **2D_Eu-L** and **3D_Eu-L**.

Compound	System	Rate Constants		τ (µs)	Φ (%)
		*k_r_* (s^−1^)	*k_nr_* (s^−1^)		
**2D_Eu-L**	Experimental	140	2784	342.0	4.8
	Calculated	150	2774	–	5.1
**3D_Eu-L**	Experimental	785	1660	409.1	32.1
	Calculated	828	1617	–	33.9

**Table 5:** Spherical atomic coordinates, charge factors (*g*), and polarizabilities (*α*) for compounds **2D_Eu-L** and **3D_Eu-L** ^1^.

Compound/Atoms	Spherical Coordinates			g	α (Å^3^)
	R (Å)	Θ (°)	Φ (°)		
**2D_Eu-L**					
O12A (i)	2.3972	92.64	234.63	0.6097	2.4255
O12A (ii)	2.3911	44.67	160.49	0.6122	2.4149
O11A	2.4089	116.68	121.66	0.5934	2.4341
O11A (iii)	2.3386	53.78	344.68	0.6083	2.4241
O1w (iii)	2.4901	65.92	75.39	0.6149	2.4089
O1w	2.5427	113.97	306.89	0.5977	2.4304
O21A (i)	2.6929	149.85	220.23	0.6024	2.4220
O21A (ii)	2.6926	41.70	255.66	0.6031	2.4171
O2	2.2698	133.80	21.33	0.3425	2.4327
**3D_Eu-L**					
O11A	2.5109	50.05	14.63	0.5857	2.4355
O12A	2.4973	5.55	121.17	0.7211	2.4229
O12B	2.3260	82.08	278.45	0.5334	2.4581
O11C	2.5598	91.54	70.52	0.5849	2.4321
O21C	2.3645	123.36	15.53	0.6825	2.4199
O1w	2.3876	82.55	135.81	0.4388	2.4504
O31B (i)	2.4962	147.78	131.28	0.6025	2.4014
O41B (i)	2.5185	145.03	254.77	0.3587	2.4264
O42B (ii)	2.4264	75.41	204.54	0.6267	2.4189

^1^ Symmetry codes: (i) 3/2 − x, 1/2 + y, 5/4 − z; (ii) 1/2 − x, −1/2 + y, 5/4 − z; and (iii) 3/2 − y, 1/2 + x, 1/4 + z.

**Table 6:** Transfer rates calculated for the transitions of compounds **2D_Eu-L** and **3D_Eu-L**.

Compound 2D_Eu-L				Compound 3D_Eu-L			
Transition	*W_ET_* (s^−1^)	Transition	*W^B^_ET_* (s^−1^)	Transition	*W_ET_* (s^−1^)	Transition	*W^B^_ET_* (s^−1^)
S_0_ ← S_1_	10^−6^	S_0_ → S_1_	-	S_0_ ← S_1_	10^−6^	S_0_ → S_1_	-
T ← S_1_	10^−5^	T → S_1_	-	T ← S_1_	10^−5^	T → S_1_	-
S_0_ ← T	10^−5^	S_0_ → T	-	S_0_ ← T	10^−5^	S_0_ → T	-
^5^D_4_ ← T	6.3 × 10^3^	^5^D_4_ → T	3.3 × 10^−15^	^5^D_4_ ← T	3.5 × 10^3^	^5^D_4_ → T	4.5 × 10^−16^
^5^D_4_ ← S_1_	1.3 × 10^1^	^5^D_4_ → S_1_	5.1 × 10^−24^	^5^D_4_ ← S_1_	4.4 × 10^2^	^5^D_4_ → S_1_	7.6 × 10^−22^
^5^D_1_ ← T	1.3	^5^D_1_ → T	3.4 × 10^−35^	^5^D_1_ ← T	3.1	^5^D_1_ → T	6.0 × 10^−37^
^5^D_0_ ← T	4.3 × 10^1^	^5^D_0_ → T	2.6 × 10^−50^	^5^D_0_ ← T	8.9 × 10^2^	^5^D_0_ → T	4.2 × 10^−42^
^5^D_1_ ← ^5^D_4_	10^−6^	^5^D_1_ → ^5^D_4_	-	^5^D_1_ ← ^5^D_4_	10^−6^	^5^D_1_ → ^5^D_4_	-
^5^D_0_ ← ^5^D_1_	10^−6^	^5^D_0_ → ^5^D_1_	-	^5^D_0_ ← ^5^D_1_	10^−6^	^5^D_0_ → ^5^D_1_	-

## Data Availability

Not applicable.

## Supplementary Materials

The following are available online at https://www.mdpi.com/article/10.3390/nano12223999/s1, Figure S1: Fragment of the structure of compound 3D_Sm-D showing the coordination of tartrate ligands around the coordination polyhedra, Figure S2: Fragment of the structure of compound 2D_Gd-D showing the ligands forming the coordination polyhedra, Figures S3–S15: TG/DTG analysis for all compounds, Figures S16–S41: Pattern-matching analysis of polycrystalline sample of all compounds, Figure S42: Pattern-matching analysis of the polycrystalline sample of compound 2D_Er-L at 130 °C, Figure S43: Pattern-matching analysis of the polycrystalline sample of compound 2D_Er-L at 230 °C, Figures S44 and S45: FTIR spectra of all compounds, Figures S46–S50: Photoluminescence measurements of chiral 3D MOFs, Figures S51–S65: Photoluminescence measurements of chiral 2D MOFs, Figures S66–S71: Decay curves and lifetime measurements of all compounds, Figures S72–S78: Circular Dichroism and Circularly Polarized Luminescence measurements, Figures S79–S80: UV-Vis measurements, Table S1: Crystallographic data and structure refinement details of compounds 2D_Sm-L, 2D_Sm-D and 2D_Eu-L, Table S2: Crystallographic data and structure refinement details of compounds 2D_Gd-L, 2D_Gd-D and 2D_Yb-L, Table S3: Selected bond lengths (Å) for Sm, Eu and Gd compounds, Table S4: Hydrogen bonding interactions (Å, °) of Sm compounds, Table S5: Hydrogen bonding interactions (Å, °) of compound 2D_Eu-L, Table S6: Hydrogen bonding interactions (Å, °) of Gd compounds, Table S7: Hydrogen bonding interactions (Å, °) of compound 2D_Yb-L, Table S8: RMSD values (Å) between D and L-based MOF pairs (Sm and Gd), and between L-enantiomers of Eu-L, Yb-L and Gd-L, using positions of all atoms as derived from single crystal X-ray diffraction data. Below, the overlapped structures of the four L enantiomers is shown, Table S9: Continuous Shape Measurements for the LnO_9_ coordination environment for 2D compounds. The lowest SHAPE values for each ion are shown in bold blue, indicating best fits, Table S10: Crystallographic data of 2D compounds. References [104,105,106,107,108,109] are cited in the Supplementary Materials.
