# Supplementary material for: Influence of Tartrate Ligand Coordination over Luminescence Properties of Chiral Lanthanide-Based Metal–Organic Frameworks
Source: Nanomaterials (Basel). 2022 Nov 13;12(22):3999. doi: 10.3390/nano12223999 (PMC9692916; doi:10.3390/nano12223999)
Supplement: Supplementary file 1 [file nanomaterials-12-03999-s001.zip › nanomaterials-2002875-supplementary.pdf]

Supplementary Materials

Influence of Tartrate Ligand Coordination over Luminescence Properties of Chiral Lanthanide-Based Metal–Organic Frameworks

Uxua Huizi-Rayo [1], Xuban Gastearena [2], Ana M. Ortuño [3], Juan M. Cuerva [3], Antonio Rodríguez-Diéguez [4], Jose Angel García [5], Jesus Ugalde [1], Jose Manuel Seco [2], Eider San Sebastian [2,]* and Javier Cepeda [2,]*

1 Donostia International Physics Center, Paseo Manuel de Lardizabal 4, 20018 Donostia, Spain
2 Departament of Applied Chemistry, Faculty of Chemistry, University of the Basque Country (UPV/EHU), 20018 Donostia, Spain
3 Department of Organic Chemistry, UEQ, C/Severo Ochoa s/n, University of Granada, 18071 Granada, Spain
4 Department of Inorganic Chemistry, UEQ, C/Severo Ochoa s/n, University of Granada, 18071 Granada, Spain
5 Departament of Physics, Faculty of Science and Technology, University of the Basque Country (UPV/EHU), 48940 Leioa, Spain
* Correspondence: eider.sansebastian@ehu.eus (E.S.S.); javier.cepeda@ehu.es (J.C.)

Contents:

S1. Materials and Physical Measurements. 2

S2. X-ray Data Collection and Structure Determination 2

S3. Photophysical properties. 5

S4. Chiroptical properties. 5

S5. Structural details of a chiral 3D MOF 6

S6. Structural details of chiral 2D MOFs. 6

S7. Synthesis of chiral 3D MOFs 11

S8. Synthesis of chiral 2D MOFs 11

S9. Continuous Shape Measurements (CShMs). 13

S10. Thermogravimetric Analysis of chiral 3D MOFs. 15

S11. Thermogravimetric Analysis of chiral 2D MOFs. 16

S12. Powder X-ray Diffraction Analysis of chiral 3D MOFs. 19

S13. Powder X-ray Diffraction Analysis of chiral 2D MOFs. 22

S14. FT-IR spectroscopy of chiral 3D MOFs. 34

S15. FT-IR spectroscopy of chiral 2D MOFs. 35

S16. Photoluminescence measurements of chiral 3D MOFs. 36

S17. Photoluminescence measurements of chiral 2D MOFs. 39

S18. Comparative luminescence of Eu(III)-based compounds. 45

S19. Lifetime measurements. 45

S20. Circular Dichroism and Circularly Polarized Luminescence measurements. 48

S21. UV-Vis measurements. 51

References 53

S1. Materials and Physical Measurements.

All chemicals were of reagent grade and were used as commercially obtained. Elemental analysis (C, H, N) were performed on an Euro EA Elemental Analyzer, whereas the metal content, determined by inductively coupled plasma (ICP-AES) was performed on a Horiba Yobin Yvon Activa spectrometer. Infrared (IR) spectra (400-4000 cm^{-1}) were recorded on a Nicolet FT-IR 6700 spectrometer in KBr pellets. Thermogravimetric analysis (TGA) was performed using a thermobalance TA Instruments, model TGAQ500 under a synthetic air (80% N_2, 20 %O_2) flux of 50 $cm^3{\cdot}min^{-1}$, from room temperature to 800 °C with heating rate of 10 °C min^{-1} and a sample size of about 10–20 mg per run.

Fluorescence excitation and emission spectra were recorded on a Varian Cary-Eclipse Fluorescence Spectrofluorimeter (at RT) and Edinburgh Instruments FLS920 spectrometer (10 K), whereas an Olympus optical microscope was employed acquire the photographs. The spectra recorded for chiral **3D_Eu-L** were acquired by connecting an optical fibre to the microscope and an Horiba iHR550 spectrophotometer equipped with UV-Vis Photocool PMT. Emission spectra were corrected for source intensity (lamp and grating) and emission spectral response (detector and grating) by standard correction curves. The time-resolved measurements were performed using a μF900 microsecond pulsed lamp (λ_{ex} = 325-345 nm) for the lanthanide(III)-based emissions or a pulsed laser diode LDH-P-C-375 (λ_{ex} = 370 nm) for the ligand-based emissions. Moreover, the quantum yield was measured in the solid state by means of a Horiba Quanta-f integrating sphere using an Oriel Instruments MS257 lamp as the excitation source and an iHR550 spectrometer from Horiba to analyse the emission. Five measurements were accomplished to properly estimate the mean and standard deviation values for each compound.

CD spectra were acquired on a Jasco J-815 or on an Olis DSM172 equipped with a Hamamatsu 150 W xenon arc lamp as light source. Circularly polarized luminescence (CPL) measurements were performed on a JASCO CPL-300 spectrophotometer.

S2. X-ray Data Collection and Structure Determination.

X-ray data collection was performed on suitable single crystals of all compounds at 100(2) K. Diffraction intensities of compound **2D_Eu-L** were collected on an Agilent Technologies Super-Nova diffractometer, which was equipped with monochromated Cu Kα radiation (λ = 1.54184 Å) and an Atlas CCD detector. The intensity data for compounds **2D_Sm-D** and **2D_Gd-D** were collected on an Agilent Technologies Super-Nova diffractometer but equipped with monochromated Mo-Kα radiation (λ = 0.71073 Å) and an Eos CCD detector. Instead, data collection of compounds **3D_Sm-D**, **2D_Sm-L**, **2D_Gd-L** and **2D_Yb-L** was done on a Bruker VENTURE area detector equipped with graphite-monochromated Mo-Kα radiation (λ = 0.71073 Å) by applying the ω-scan method. The data reduction was performed with the CrysAlis Pro software package [1] or APEX2 [2] software and corrected for absorption using SADABS [3], depending on the diffractometer used. Crystal structures were solved (and partially solved in the case of **3D_Sm-L**) by direct methods using the SIR97 program [4] and refined by full-matrix least squares on F^2 including all reflections using anisotropic displacement parameters by means of the WINGX crystallographic package [5]. During the data integration of compound **3D_Sm-D**, it was observed that unit cell only indexed about half of the reflections, suggesting the occurrence of twinned specimens as observed for other previously reported counterparts. Unusually large ADPs observed for many atoms as well as large R_1 and wR^2 agreement factors during the refinement stage further confirmed the latter. Accordingly, all structures were refined with the general twin law [(-1 0 0 / -0.02 0.96 -0.07 / 0.01 -0.98 -0.96) including the two crystallographic domains (all reflections) in such a way that final percentage for the minor domain was estimated to be of 8%. However, some strong unassigned electron density maxima remained close to samarium(III) atoms, which precluded further refinement of this compound. Hydrogen atoms belonging to the tartrate ligands were located in the difference Fourier map and included with riding models as fixed contributions with isotropic thermal displacement parameters 1.2 and 1.5 times those of their parent carbon (C–H) and oxygen (O–H) atoms, respectively. Note that those hydrogen

atoms belonging to O–H groups (hydroxyl or water molecules) were refined according to the optimized hydrogen bonding interaction. The structure refinement converges with Flack parameters equal to 0.04(1), -0.005(8), -0.03(1), -0.07(1), 0.006(3), -0.02(1) and 0.007(4) respectively for **3D_Sm-L**, **2D_Sm-L**, **2D_Sm-D**, **2D_Eu-L**, **2D_Gd-L**, **2D_Gd-D** and **2D_Yb-L**, which confirms the chiral purity of all specimens. CCDC 2157993-2157998 contains the supplementary crystallographic data for this communication. These data can be obtained free of charge via http://www.ccdc.cam.ac.uk/conts/retrieving.html (or from the Cambridge Crystallographic Data Centre, 12, Union Road, Cambridge CB2 1EZ, UK; fax: +44 1223 336033). Crystallographic and refinement data are summarized in Tables S1–2. The X-ray powder diffraction (XRPD) patterns were collected at 25 °C on a Phillips X'PERT powder diffractometer with Cu-K_α radiation (λ = 1.5418 Å) over the range $5 < 2\theta < 50^{\circ}$ with a step size of 0.02° and an acquisition time of 2.5 s per step. Indexation of the diffraction profiles were made by means of the FULLPROF program (pattern-matching analysis) [6] based on the space group and the cell parameters found by single crystal X-ray diffraction. Variable-temperature powder X-ray diffraction measurements were conducted on polycrystalline sample of compound **2D_Er-L** under ambient atmosphere with heating rate of 5 °C·min^{-1} and measuring a complete diffractogram every 20 °C up to 450 °C, and every 50 °C from 500 °C up to 800 °C.

Table S1. Crystallographic data and structure refinement details of compounds **2D_Sm-L**, **2D_Sm-D** and **2D_Eu-L**.

	**3D_Sm-D**	**2D_Sm-L**	**2D_Sm-D**	**2D_Eu-L**
Empirical formula	$C_{12}H_{22}O_{23}Sm_2$	$C_8H_{15}O_{15}Sm$	$C_8H_{15}O_{15}Sm$	$C_8H_{15}EuO_{15}$
Formula weight	834.99	501.55	501.55	503.16
Crystal system	Triclinic	Tetragonal	Tetragonal	Tetragonal
Space group	$P1$	$P4_12_12$	$P4_32_12$	$P4_12_12$
a (Å)	6.0560(8)	6.0849(2)	6.0704(1)	6.06290(10)
b (Å)	7.4446(8)	6.0849(2)	6.0704(1)	6.06290(10)
c (Å)	13.3099(17)	36.5456(14)	36.4666(7)	36.4986(6)
α (°)	102.229(4)	90	90	90
β (°)	101.431(5)	90	90	90
γ (°)	90.485(4)	90	90	90
V (Å^3)	574.02(12)	1353.14(10)	1343.79(5)	1341.64(5)
Z	1	4	4	4
μ (mm^{-1})	5.174	4.431	4.462	34.372
ρ (g cm^{-3})	2.415	2.462	2.479	2.491
Crystal size (mm^3)	0.20 × 0.09 × 0.06	0.11 × 0.08 × 0.06	0.08 × 0.06 × 0.05	0.10 x 0.07 × 0.05
F(000)	402	980	980	984
Unique reflections/total	4411/11685	1773/10789	1270/9120	1373/10412
GOF [a]	1.169	1.129	1.176	1.200
R_{int}	0.0608	0.0296	0.0351	0.0486

Final R indices				
R_1 [b] / wR_2 [c] [I>2σ(I)]	0.1165 / 0.3232	0.0224 / 0.0531	0.0206 / 0.0547	0.0337 / 0.0809
R_1 / wR_2 (all data)	0.1265 / 0.3278	0.0257 / 0.0539	0.0216 / 0.0551	0.0349 / 0.0813

[a]S = $[\sum w(F_0^2 - F_c^2)^2 / (N_{obs} - N_{param})]^{1/2}$. [b] $R_1 = \sum||F_0|-|F_c|| / \sum|F_0|$. [c] $wR_2 = [\sum w(F_0^2 - F_c^2)^2 / \sum wF_0^2]^{1/2}$; $w = 1/[\sigma^2(F_0^2) + (aP)^2 + bP]$ where $P = (max(F_0^2,0) + 2F_c^2)/3$.

Table S2. Crystallographic data and structure refinement details of compounds **2D_Gd-L**, **2D_Gd-D** and **2D_Yb-L**.

	**2D_Gd-L**	**2D_Gd-D**	**2D_Yb-L**
**Empirical formula**	$C_8H_{15}GdO_{15}$	$C_8H_{15}GdO_{15}$	$C_8H_{15}O_{15}Yb$
**Formula weight**	508.44	508.44	524.24
**Crystal system**	Tetragonal	Tetragonal	Tetragonal
**Space group**	$P4_12_12$	$P4_32_12$	$P4_12_12$
a **(Å)**	6.0454(5)	6.05110(10)	5.9661(3)
b **(Å)**	6.0454(5)	6.05110(10)	5.9661(3)
c **(Å)**	36.403(3)	36.4168(7)	36.218(3)
α (°)	90	90	90
β (°)	90	90	90
γ (°)	90	90	90
**V (Å^3)**	1330.4(2)	1333.43(5)	1289.16(16)
**Z**	4	4	4
**μ (mm^{-1})**	5.078	5.066	7.350
**ρ (g cm^{-3})**	2.538	2.533	2.701
**Crystal size (mm^3)**	0.10 × 0.08 × 0.06	0.08 × 0.07 × 0.06	0.09 × 0.08 × 0.06
**F(000)**	988	988	1012
**Unique reflections/total**	1742/45613	1228/8456	1675/40972
**GOF [a]**	1.240	1.114	1.312
**R_{int}**	0.0237	0.0523	0.0249
**Final R indices**			
**R_1 [b] / wR_2[c] [I>2 (I)]**	0.0178 / 0.0505	0.0237 / 0.0586	0.0168 / 0.0474
**R_1 / wR_2 (all data)**	0.0180 / 0.0507	0.0265 / 0.0593	0.0174 / 0.0475

[a] $S = [\sum w(F_0^2 - F_c^2)^2 / (N_{obs} - N_{param})]^{1/2}$. [b] $R_1 = \sum||F_0|-|F_c|| / \sum|F_0|$. [c] $wR_2 = [\sum w(F_0^2 - F_c^2)^2 / \sum wF_0^2]^{1/2}$; $w = 1/[\sigma^2(F_0^2) + (aP)^2 + bP]$ where $P = (max(F_0^2,0) + 2F_c^2)/3$.

S3. Photophysical properties.

Fluorescence excitation and emission spectra recorded at room temperature were performed on a Varian Cary-Eclipse Fluorescence Spectrofluorimeter equipped with a xenon discharge lamp (peak power equivalent to 75 kW), Czerny–Turner monochromators, and an R-928 photomultiplier tube. For the fluorescence measurements, the photomultiplier detector voltage was fixed at 600 V while the excitation and emission slits were set at 5 and 2.5 nm.

PL measurements at 10 K were done using a close cycle helium cryostat enclosed in an Edinburgh Instruments FLS920 spectrometer, in such a way that the applied vacuum (10^{-7} bar) protected the samples from spontaneous rehydration. For steady state measurements in the UV-Vis range an IK3552R-G HeCd continuous laser (325 nm) was used as excitation source, whereas a Müller-Elektronik-Optik SVX1450 Xe lamp was employed to collect the excitation spectra. Photographs of crystalline samples were obtained in a micro-PL system included in an Olympus optical microscope illuminated with a HeCd laser or a Hg lamp.

S4. Chiroptical properties.

CD spectra were recorded at 25 °C on a Jasco J-815 or on an Olis DSM172 equipped with a Hamamatsu 150 W xenon arc lamp as light source. 1 cm path-length cuvettes and the following parameters were used for data acquisition on Jasco J-815: data pitch 0.5 nm; digital integration time (D.I.T.) 1 sec; scanning speed 200 nm/min; bandwidth 1 nm; accumulations 4. HT voltage was controlled (HT ≤ 800) to give reliable ellipticity values over the investigated wavelength range. CD raw data were processed with Jasco Spectra Manager 2 (Jasco) and Origin 9.5 (OriginLab Corp.).

Circularly polarized luminescence (CPL) measurements were performed on an Olis DSM172 spectrophotometer equipped with fixed wavelengths (355, 365 and 370 nm) LEDs as light source. A Hamamatsu photomultiplier tube was used as detector for ECD and a Hamamatsu photon counting detector for CPL measurements. Different settings and data processing were selected and carried out using the Olis GlobalWorks software. A fixed bandwidth of 1.0 nm and an integration time of 0.10 s were selected for ECD measurements, whilst for CPL experiments 1.00 s was selected as integration time.

S5. Structural details of a chiral 3D MOF.

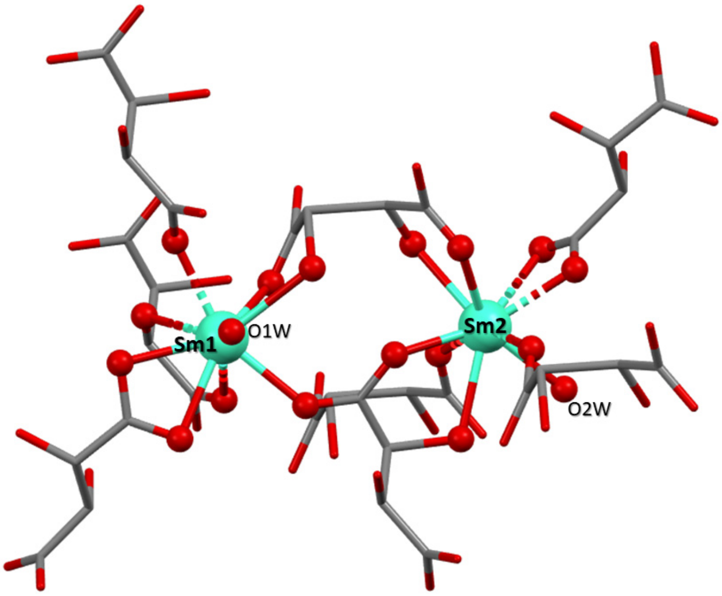

Figure S1. Fragment of the structure of compound 3D_Sm-D showing the coordination of tartrate ligands around the coordination polyhedra.

S6. Structural details of chiral 2D MOFs.

Table S3. Selected bond lengths (Å) for Sm, Eu and Gd compounds.[a].

Compound **2D_Sm-L**		Compound **2D_Sm-D**	
Sm1–O12A(i)	2.370(3)	Sm1–O12A(iv)	2.363(4)
Sm1–O12A(ii)	2.370(3)	Sm1–O12A(v)	2.363(4)
Sm1–O11A	2.376(3)	Sm1–O11A(vi)	2.371(4)
Sm1–O11A(iii)	2.376(3)	Sm1–O11A	2.371(4)
Sm1–O1W(iii)	2.457(4)	Sm1–O1W	2.450(5)
Sm1–O1W	2.457(4)	Sm1–O1W(vi)	2.450(5)
Sm1–O21A(i)	2.542(3)	Sm1–O21A(v)	2.536(4)
Sm1–O21A(ii)	2.542(3)	Sm1–O21A(iv)	2.536(4)
Sm1–O2	2.707(9)	Sm1–O2	2.698(11)
Compound **2D_Eu-L**		Compound **2D_Gd-L**	
Eu1–O12A(i)	2.350(5)	Gd1–O12A(i)	2.342(3)
Eu1–O12A(ii)	2.350(5)	Gd1–O12A(ii)	2.342(3)
Eu1–O11A(iii)	2.362(5)	Gd1–O11A	2.354(3)
Eu1–O11A	2.362(5)	Gd1–O11A(iii)	2.354(3)
Eu1–O1W(iii)	2.442(6)	Gd1–O1W	2.423(4)
Eu1–O1W	2.442(6)	Gd1–O1W(iii)	2.423(4)

Eu1–O21A(i)	2.530(5)	Gd1–O21A(i)	2.512(3)
Eu1–O21A(ii)	2.530(5)	Gd1–O21A(ii)	2.512(3)
Eu1–O2	2.693(14)	Gd1–O2	2.670(8)
Compound **2D_Gd-D**		Compound **2D_Yb-L**	
Gd1–O12A(iv)	2.348(4)	Yb1–O12A(i)	2.277(3)
Gd1–O12A(v)	2.348(4)	Yb1–O12A(ii)	2.277(3)
Gd1–O11A(vi)	2.353(5)	Yb1–O11A	2.289(3)
Gd1–O11A	2.353(5)	Yb1–O11A(iii)	2.289(3)
Gd1–O1W	2.423(5)	Yb1–O1W(iii)	2.341(4)
Gd1–O1W(vi)	2.423(5)	Yb1–O1W	2.341(4)
Gd1–O21A(v)	2.517(4)	Yb1–O21A(i)	2.456(3)
Gd1–O21A(iv)	2.517(4)	Yb1–O21A(ii)	2.456(3)
Gd1–O2	2.690(13)	Yb1–O2	2.627(10)

[a] Symmetries: (i) y-1, x, -z+1; (ii) x-1, y, z; (iii) y-1, x+1, -z+1; (iv) x+1, y, z; (v) y+1, x, -z+1; (vi) y+1, x-1, -z+1.

Table S4. Hydrogen bonding interactions (Å, °) of Sm compounds.[a].

D–H···A[b]	D–H	H···A	D···A	D–H···A
**Compound 2D_Sm-L**				
O21A–H21A···O42A(i)	0.84	1.83	2.653(5)	166.4
O31A–H31A···O41A(ii)	0.91	2.02	2.829(6)	148.1
O41A–H41A···O1W(iii)	0.86	1.81	2.637(6)	163.1
O1W–H11W···O12A	0.87	1.89	2.706(5)	156.2
O1W–H12W···O31A(iv)	0.84	1.94	2.759(6)	165.1
O2–H2···O31A(v)	0.87	2.11	2.934(7)	158.9
**Compound 2D_Sm-D**				
O21A–H21A···O42A(vi)	0.84	1.83	2.653(5)	166.4
O31A–H31A···O41A(vii)	0.91	2.02	2.829(6)	148.1
O41A–H41A···O1W(viii)	0.86	1.81	2.637(6)	163.1
O1W–H11W···O12A	0.87	1.89	2.706(5)	156.2
O1W–H12W···O31A(iv)	0.84	1.94	2.759(6)	165.1
O2–H2···O31A(ix)	0.87	2.11	2.934(7)	158.9

[a] Symmetry codes: (i) -x+3/2, y+1/2, -z+5/4; (ii) -x+1/2, y-1/2, -z+5/4; (iii) -y+3/2, x+1/2, z+1/4; (iv) y, x, -z+1; (v) x, y+1, z; (vi) -x+1/2, y-1/2, -z+3/4; (vii) -x+3/2, y+1/2, -z+3/4; (viii) -x+3/2, y+1/2, z-1/4; (ix) y, x-1, -z+1. [b] D: donor. A: acceptor.

Table S5. Hydrogen bonding interactions (Å, °) of compound **2D_Eu-L**.[a].

**D–H···A[b]**	**D–H**	**H···A**	**D···A**	**D–H···A**
O21A–H21A···O42A(i)	0.85	1.81	2.660(7)	175.2
O31A–H31A···O41A(ii)	0.89	2.01	2.826(9)	151.7
O41A–H41A···O1W(iii)	0.86	1.79	2.636(8)	168.8
O1W–H11W···O12A	0.87	1.88	2.711(7)	159.9
O1W–H12W···O31A(iv)	0.86	1.91	2.745(10)	164.9
O2–H2···O31A(v)	0.86	2.07	2.916(10)	167.2

[a] Symmetry codes: (i) -x+3/2, y+1/2, -z+5/4; (ii) -x+1/2, y-1/2, -z+5/4; (iii) -y+3/2, x+1/2, z+1/4; (iv) y, x, -z+1; (v) x, y+1, z. [b] D: donor. A: acceptor.

Table S6. Hydrogen bonding interactions (Å, °) of Gd compounds.[a]

D–H···A[b]	D–H	H···A	D···A	D–H···A
**Compound 2D_Gd-L**				
O21A–H21A···O42A(i)	0.85	1.81	2.652(4)	175.1
O31A–H31A···O41A(ii)	0.90	2.01	2.828(5)	149.9
O41A–H41A···O1W(iii)	0.85	1.80	2.627(5)	163.5
O1W–H11W···O12A	0.87	1.87	2.694(4)	156.7
O1W–H12W···O31A(iv)	0.86	1.91	2.751(6)	166.1
O2–H2···O31A(v)	0.88	2.08	2.913(6)	158.5
**Compound 2D_Gd-D**				
O21A–H21A···O42A(vi)	0.86	1.81	2.655(6)	170.9
O31A–H31A···O41A(vii)	0.88	2.03	2.829(8)	150.9
O41A–H41A···O1W(viii)	0.86	1.79	2.624(7)	164.5
O1W–H11W···O12A	0.87	1.86	2.693(6)	162.5
O1W–H12W···O31A(iv)	0.88	1.90	2.757(8)	165.6
O2–H2···O31A(ix)	0.85	2.06	2.908(10)	168.9

[a] Symmetry codes: (i) -x+3/2, y+1/2, -z+5/4; (ii) -x+1/2, y-1/2, -z+5/4; (iii) -y+3/2, x+1/2, z+1/4; (iv) y, x, -z+1; (v) x, y+1, z; (vi) -x+1/2, y-1/2, -z+3/4; (vii) -x+3/2, y+1/2, -z+3/4; (viii) -y+1/2, x-1/2, z-1/4; (ix) y, x-1, -z+1. [b] D: donor. A: acceptor.

Table S7. Hydrogen bonding interactions (Å, °) of compound **2D_Yb-L**.[a]

D–H···A[b]	D–H	H···A	D···A	D–H···A
O21A–H21A···O42A(i)	0.84	1.81	2.653(5)	177.1
O31A–H31A···O41A(ii)	0.85	2.07	2.815(5)	146.8
O41A–H41A···O1W(iii)	0.85	1.78	2.623(5)	170.3
O1W–H11W···O12A	0.87	1.85	2.682(5)	158.2
O1W–H12W···O31A(iv)	0.87	1.89	2.753(6)	169.1
O2–H2···O31A(v)	0.87	2.04	2.862(7)	157.9

[a] Symmetry codes: (i) -x+3/2, y+1/2, -z+5/4; (ii) -x+1/2, y-1/2, -z+5/4; (iii) -y+3/2, x+1/2, z+1/4; (iv) y, x, -z+1; (v) x, y+1, z. [b] D: donor. A: acceptor.

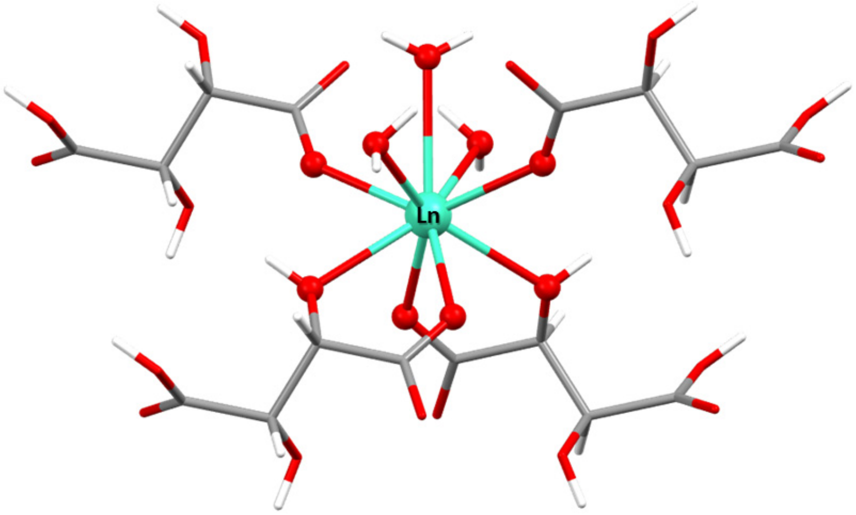

Figure S2. Fragment of the structure of compound 2D_Gd-D showing the ligands forming the coordination polyhedra.

Table S8. RMSD values (Å) between D and L-based MOF pairs (Sm and Gd), and between L-enantiomers of Eu-L, Yb-L and Gd-L, using positions of all atoms as derived from single crystal X-ray diffraction data. Below, the overlapped structures of the four L enantiomers is shown.

	**Sm-L/Sm-D**	**Gd-L/Gd-D**	**Eu-L/Gd-L**	**Yb-L/Gd-L**
**RMSD (Å)**	**0.007**	**0.0097**	**0.008**	**0.00117**

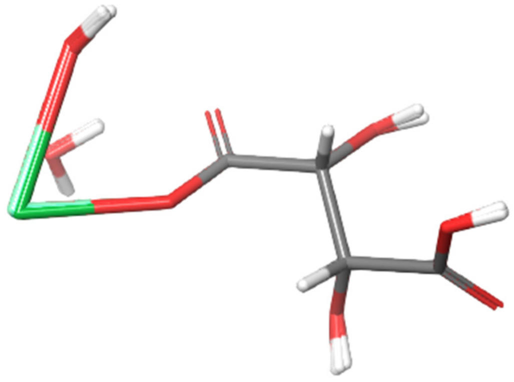

S7. Synthesis of chiral 3D MOFs

Compound 3D_Sm-L: 5 mL of an aqueous solution containing 0.6 mmol of $Sm(NO_3)_3·6H_2O$ (266.7 mg) were mixed with an aqueous solution of L-tartaric acid (L-H_2Tart) (135.1 mg, 0.9 mmol) in 5 mL and sonicated. The resulting solution was heated in a Teflon liner at 160 °C for 48 h. After slow cooling to room temperature, colourless prismatic single crystals **3D_Sm-L** were obtained, which were collected by filtration and washed several times with water. Elemental analysis for $C_{12}H_{24}O_{24}Sm_2$ (853.08 g mol^{-1}). Calcd.: C, 16.89; H, 2.84; Sm, 35.25. Found: C, 17.02; H, 2.95; Sm, 35.19.

Compound 3D_Sm-D: Compound **3D_Sm-D** was synthesized by the same method used with compound **3D_Sm-L** using 0.9 mmol (135.1 mg) of D-tartaric acid (D-H_2Tart), obtaining colourless prismatic single crystals. Elemental analysis for $C_{12}H_{24}O_{24}Sm_2$ (853.08 g mol^{-1}). Calcd.: C, 16.89; H, 2.84; Sm, 35.25. Found: C, 16.72; H, 2.69; Sm, 35.33.

Compound 3D_Eu-L: Compound **3D_Eu-L** was synthesized by the same method used with compound **3D_Sm-L** using 0.6 mmol (267.6 mg) of $Eu(NO_3)_3·6H_2O$, obtaining colourless prismatic single crystals. Elemental analysis for $C_{12}H_{24}Eu_2O_{24}$ (856.28 g mol^{-1}). Calcd.: C, 16.83; H, 2.83; Eu, 35.49. Found: C, 16.98; H, 2.55; Eu, 35.66.

Compound 3D_Eu-D: Compound **3D_Eu-D** was synthesized by the same method used with compound **3D_Eu-L** using 0.9 mmol (135.1 mg) of D-tartaric acid (D-H_2Tart), obtaining colourless prismatic single crystals. Elemental analysis for $C_{12}H_{24}Eu_2O_{24}$ (856.28 g mol^{-1}). Calcd.: C, 16.83; H, 2.83; Eu, 35.49. Found: C, 16.74; H, 2.98; Eu, 35.37.

Compound 3D_Gd-L: Compound **3D_Gd-L** was synthesized by the same method used with compound **3D_Sm-L** using 0.6 mmol (270.8 mg) of $Gd(NO_3)_3·6H_2O$, obtaining colourless prismatic single crystals. Elemental analysis for $C_{12}H_{24}Gd_2O_{24}$ (866.86 g mol^{-1}). Calcd.: C, 16.63; H, 2.80; Gd, 36.28. Found: C, 16.54; H, 2.75; Gd, 36.22.

Compound 3D_Gd-D: Compound **3D_Gd-D** was synthesized by the same method used with compound **3D_Gd-L** using 0.9 mmol (135.1 mg) of D-tartaric acid (D-H_2Tart), obtaining pinkish prismatic single crystals. Elemental analysis for $C_{12}H_{24}Gd_2O_{24}$ (866.86 g mol^{-1}). Calcd.: C, 16.63; H, 2.80; Gd, 36.28. Found: C, 16.69; H, 2.75; Gd, 36.40.

S8. Synthesis of chiral 2D MOFs

Compound 2D_Y-L: 4 mL of an aqueous solution containing 0.4 mmol of $Y(NO_3)_3·6H_2O$ (153.2 mg) were mixed with an aqueous solution of L-tartaric acid (L-H_2Tart) (120.07 mg, 0.8 mmol) in 4 mL and sonicated. The resulting solution was evaporated in a vial heating at 50 °C. After 24 h, colourless prismatic single crystals **2D_Y-L** were obtained, which were collected by filtration and washed several times with water. Elemental analysis for $C_8H_{15}O_{15}Y$ (440.12 g mol^{-1}). Calcd.: C, 21.83; H, 3.44; Y, 20.20. Found: C, 22.07; H, 3.19; Y, 20.36.

Compound 2D_Y-D: Compound **2D_Y-D** was synthesized by the same method used with compound **2D_Y-L** using 0.8 mmol (120.1 mg) of D-tartaric acid (D-H_2Tart), obtaining colourless prismatic single crystals. Elemental analysis for $C_8H_{15}O_{15}Y$ (440.12 g mol^{-1}). Calcd.: C, 21.83; H, 3.44; Y, 20.20. Found: C, 21.88; H, 3.34; Y, 20.40.

Compound 2D_Sm-L: Compound **2D_Sm-L** was synthesized by the same method used with compound **2D_Y-L** using 0.4 mmol (177.8 mg) of $Sm(NO_3)_3·6H_2O$, obtaining colourless prismatic single crystals. Elemental analysis for $C_8H_{15}O_{15}Sm$ (501.57 g mol^{-1}). Calcd.: C, 19.16; H, 3.02; Sm, 29.98. Found: C, 19.30; H, 2.94; Sm, 30.05.

Compound 2D_Sm-D: Compound **2D_Sm-D** was synthesized by the same method used with compound **2D_Sm-L** using 0.8 mmol (120.1 mg) of D-tartaric acid (D-H_2Tart), obtaining colourless prismatic single crystals. Elemental analysis for $C_8H_{15}O_{15}Sm$ (501.57 g mol^{-1}). Calcd.: C, 19.16; H, 3.02; Sm, 29.98. Found: C, 19.60; H, 2.83; Sm, 29.83.

Compound 2D_Eu-L: Compound **2D_Eu-L** was synthesized by the same method used with compound **2D_Y-L** using 0.4 mmol (178.4 mg) of $Eu(NO_3)_3·6H_2O$, obtaining colourless prismatic single crystals. Elemental analysis for $C_8H_{15}EuO_{15}$ (503.17 g mol^{-1}). Calcd.: C, 19.09; H, 3.01; Eu, 30.20. Found: C, 19.11; H, 2.90; Eu, 30.35.

Compound 2D_Eu-D: Compound **2D_Eu-D** was synthesized by the same method used with compound **2D_Eu-L** using 0.8 mmol (120.1 mg) of D-tartaric acid (D-H_2Tart),

obtaining colourless prismatic single crystals. Elemental analysis for $C_8H_{15}EuO_{15}$ (503.17 g mol^{-1}). Calcd.: C, 19.09; H, 3.01; Eu, 30.20. Found: C, 19.23; H, 2.75; Eu, 30.08.

Compound 2D_Gd-L: Compound **2D_Gd-L** was synthesized by the same method used with compound **2D_Y-L** using 0.4 mmol (180.5 mg) of $Gd(NO_3)_3{\cdot}6H_2O$, obtaining colourless prismatic single crystals. Elemental analysis for $C_8H_{15}GdO_{15}$ (508.44 g mol^{-1}). Calcd.: C, 18.87; H, 2.96; Gd, 30.93. Found: C, 18.76; H, 2.83; Gd, 31.08.

Compound 2D_Gd-D: Compound **2D_Gd-D** was synthesized by the same method used with compound **2D_Gd-L** using 0.8 mmol (120.1 mg) of D-tartaric acid (D-H_2Tart), obtaining colourless prismatic single crystals. Elemental analysis for $C_8H_{15}GdO_{15}$ (508.44 g mol^{-1}). Calcd.: C, 18.87; H, 2.96; Gd, 30.93. Found: C, 18.75; H, 2.84; Gd, 30.85.

Compound 2D_Tb-L: Compound **2D_Tb-L** was synthesized by the same method used with compound **2D_Y-L** using 0.4 mmol (181.2 mg) of $Tb(NO_3)_3{\cdot}6H_2O$, obtaining colourless prismatic single crystals. Elemental analysis for $C_8H_{15}O_{15}Tb$ (510.12 g mol^{-1}). Calcd.: C, 18.83; H, 2.97; Tb, 31.16. Found: C, 18.75; H, 2.83; Tb, 31.07.

Compound 2D_Tb-D: Compound **2D_Tb-D** was synthesized by the same method used with compound **2D_Tb-L** using 0.8 mmol (120.1 mg) of D-tartaric acid (D-H_2Tart), obtaining colourless prismatic single crystals. Elemental analysis for $C_8H_{15}O_{15}Tb$ (510.12 g mol^{-1}). Calcd.: C, 18.83; H, 2.97; Tb, 31.16. Found: C, 19.02; H, 3.08; Tb, 31.05.

Compound 2D_Dy-L: Compound **2D_Dy-L** was synthesized by the same method used with compound **2D_Y-L** using 0.4 mmol (175.4 mg) of $Dy(NO_3)_3{\cdot}5H_2O$, obtaining colourless prismatic single crystals. Elemental analysis for $C_8H_{15}DyO_{15}$ (513.71 g mol^{-1}). Calcd.: C, 18.70; H, 2.95; Dy, 31.63. Found: C, 18.78; H, 2.81; Dy, 31.59.

Compound 2D_Dy-D: Compound **2D_Dy-D** was synthesized by the same method used with compound **2D_Dy-L** using 0.8 mmol (120.1 mg) of D-tartaric acid (D-H_2Tart), obtaining colourless prismatic single crystals. Elemental analysis for $C_8H_{15}DyO_{15}$ (513.71 g mol^{-1}). Calcd.: C, 18.70; H, 2.95; Dy, 31.63. Found: C, 18.88; H, 3.05; Dy, 31.53.

Compound 2D_Ho-L: Compound **2D_Ho-L** was synthesized by the same method used with compound **2D_Sm-L** using 0.4 mmol (183.6 mg) of $Ho(NO_3)_3{\cdot}6H_2O$, obtaining pinkish prismatic single crystals. Elemental analysis for $C_8H_{15}HoO_{15}$ (516.14 g mol^{-1}). Calcd.: C, 18.62; H, 2.93; Ho, 31.95. Found: C, 18.53; H, 2.72; Ho, 31.83.

Compound 2D_Ho-D: Compound **2D_Ho-D** was synthesized by the same method used with compound **2D_Ho-L** using 0.8 mmol (120.1 mg) of D-tartaric acid (D-H_2Tart), obtaining pinkish prismatic single crystals. Elemental analysis for $C_8H_{15}HoO_{15}$ (516.14 g mol^{-1}). Calcd.: C, 18.62; H, 2.93; Ho, 31.95. Found: C, 18.76; H, 2.84; Ho, 31.87.

Compound 2D_Er-L: Compound **2D_Er-L** was synthesized by the same method used with compound **2D_Y-L** using 0.4 mmol (177.3 mg) of $Er(NO_3)_3{\cdot}5H_2O$, obtaining pinkish prismatic single crystals. Elemental analysis for $C_8H_{15}ErO_{15}$ (518.47 g mol^{-1}). Calcd.: C, 18.53; H, 2.92; Er, 32.26. Found: C, 18.42; H, 2.77; Er, 32.01.

Compound 2D_Er-D: Compound **2D_Er-D** was synthesized by the same method used with compound **2D_Er-L** using 0.8 mmol (120.1 mg) of D-tartaric acid (D-H_2Tart), obtaining pinkish prismatic single crystals. Elemental analysis for $C_8H_{15}ErO_{15}$ (518.47 g mol^{-1}). Calcd.: C, 18.53; H, 2.92; Er, 32.26. Found: C, 18.45; H, 2.84; Er, 32.19.

Compound 2D_Tm-L: Compound **2D_Tm-L** was synthesized by the same method used with compound **2D_Y-L** using 0.4 mmol (185.2 mg) of $Tm(NO_3)_3{\cdot}6H_2O$, obtaining colourless prismatic single crystals. Elemental analysis for $C_8H_{15}O_{15}Tm$ (520.14 g mol^{-1}). Calcd.: C, 18.47; H, 2.91; Tm, 32.48. Found: C, 18.26; H, 2.78; Tm, 32.45.

Compound 2D_Tm-D: Compound **2D_Tm-D** was synthesized by the same method used with compound **2D_Tm-L** using 0.8 mmol (120.1 mg) of D-tartaric acid (D-H_2Tart), obtaining colourless prismatic single crystals. Elemental analysis for $C_8H_{15}O_{15}Tm$ (520.14 g mol^{-1}). Calcd.: C, 18.47; H, 2.91; Tm, 32.48. Found: C, 18.29; H, 2.82; Tm, 32.56.

Compound 2D_Yb-L: Compound **2D_Yb-L** was synthesized by the same method used with compound **2D_Y-L** using 0.4 mmol (179.6 mg) of $Yb(NO_3)_3{\cdot}5H_2O$, obtaining colourless prismatic single crystals. Elemental analysis for $C_8H_{15}O_{15}Yb$ (524.25 g mol^{-1}). Calcd.: C, 18.33; H, 2.89; Yb, 33.01. Found: C, 18.37; H, 2.82; Yb, 33.09.

Compound 2D_Yb-D: Compound **2D_Yb-D** was synthesized by the same method used with compound **2D_Yb-L** using 0.8 mmol (120.1 mg) of D-tartaric acid (D-H_2Tart), obtaining colourless prismatic single crystals. Elemental analysis for $C_8H_{15}O_{15}Yb$ (524.25 g mol^{-1}). Calcd.: C, 18.33; H, 2.89; Yb, 33.01. Found: C, 18.12; H, 2.71; Yb, 32.89.

S9. Continuous Shape Measurements (CShMs).

Table S9. Continuous Shape Measurements for the LnO_9 coordination environment for 2D compounds. The lowest SHAPE values for each ion are shown in bold blue, indicating best fits.

Codes:

EP-9	1 D9h	Enneagon
OPY-9	2 C8v	Octagonal pyramid
HBPY-9	3 D7h	Heptagonal bipyramid
JTC-9	4 C3v	Johnson triangular cupola J3
JCCU-9	5 C4v	Capped cube J8
CCU-9	6 C4v	Spherical-relaxed capped cube
JCSAPR-9	7 C4v	Capped square antiprism J10
CSAPR-9	8 C4v	Spherical capped square antiprism
JTCTPR-9	9 D3h	Tricapped trigonal prism J51
TCTPR-9	10 D3h	Spherical tricapped trigonal prism
JTDIC-9	11 C3v	Tridiminished icosahedron J63
HH-9	12 C2v	Hula-hoop
MFF-9	13 Cs	Muffin

Structure [ML_9]	2D_Sm-L	2D_Sm-D	2D_Eu-L	2D_Gd-L	2D_Gd-D	2D_Yb-L
**EP-9**	31.958	31.930	31.957	27.469	31.747	31.405
**OPY-9**	23.480	23.484	23.477	20.554	23.648	24.024
**HBPY-9**	17.762	17.783	17.847	16.604	17.989	18.458
**JTC-9**	13.209	13.191	13.231	**8.869**	13.220	13.306
**JCCU-9**	9.92	9.921	9.941	13.843	9.973	10.044
**CCU-9**	8.944	8.953	8.992	11.717	8.992	9.097
**JCSAPR-9**	2.128	2.132	2.110	12.202	2.009	1.877
**CSAPR-9**	1.800	1.791	1.775	10.425	1.742	1.695
**JTCTPR-9**	**1.235**	**1.228**	**1.190**	11.397	**1.210**	**1.129**
**TCTPR-9**	1.561	1.554	1.539	10.782	1.582	1.584
**JTDIC-9**	13.176	13.151	13.242	13.243	13.282	13.460

**HH-9**	11.294	11.330	11.308	11.615	11.258	11.300
**MFF-9**	2.359	2.348	2.340	9.579	2.328	2.347

S10. Thermogravimetric Analysis of chiral 3D MOFs.

The thermogravimetric behaviour of all compounds is very similar given their isostructural nature. The general behaviour accounting for the most common $\{[Ln_2(\mu_4\text{-}tar)_2(\mu\text{-}tar)(H_2O)_2]\cdot 4H_2O\}_n$ formula is described. Heating of the sample starts with a short plateau in the TG curve from room temperature up to ca. 70 ºC, after which three lattice water molecules present in the channels as well as one coordination water molecule are lost. This first dehydration finishes at ca. 120 ºC, which yields a new compounds of $\{[Ln_2(\mu_4\text{-}tart)_2(\mu\text{-}tart)(H_2O)]\cdot H_2O\}_n$ formula. Then, after a small plateau of ca. 30 ºC, materials experiment the loss of one lattice and one coordination water molecules, which finishes at ca. 210 ºC and yields a dehydrated phase of the compound that shows no mass loss up to ca. 290 ºC. Above the latter temperature, all compounds present two main exothermic processes which involve to the decomposition of tartrate ligands, leading to the corresponding Ln_2O_3 as a final residue.

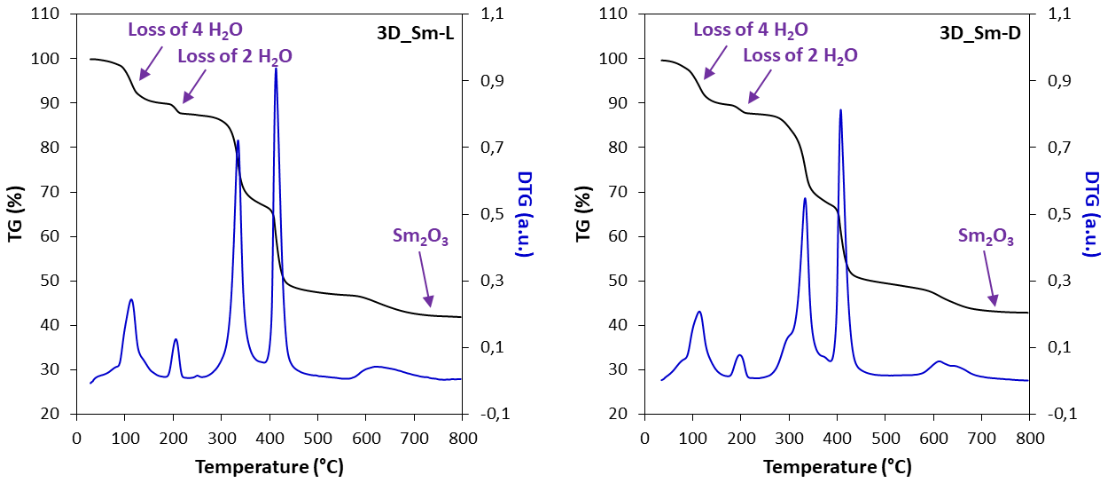

Figure S3. TG/DTG analysis for compounds 3D_Sm-L and 3D_Sm-D.

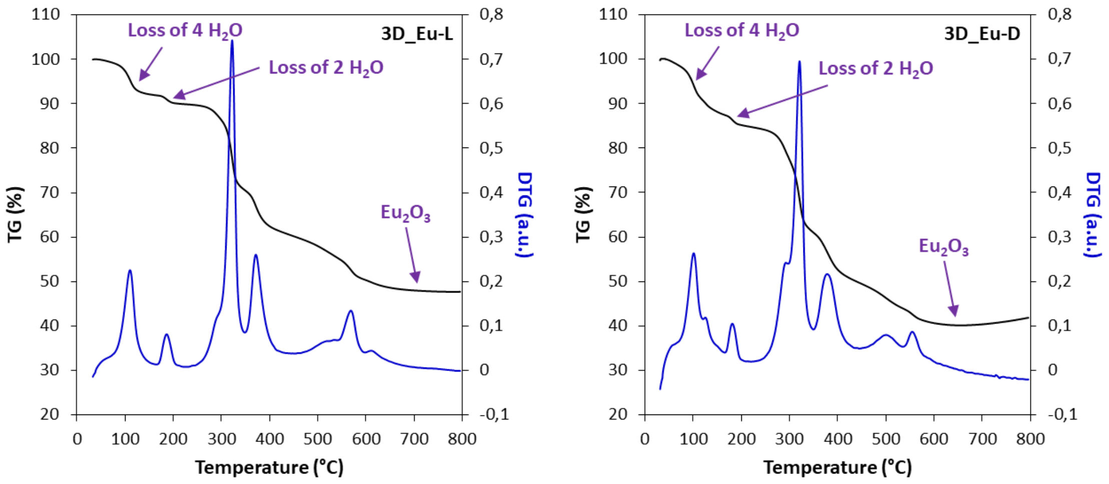

Figure S4. TG/DTG analysis for compounds 3D_Eu-L and 3D_Eu-D.

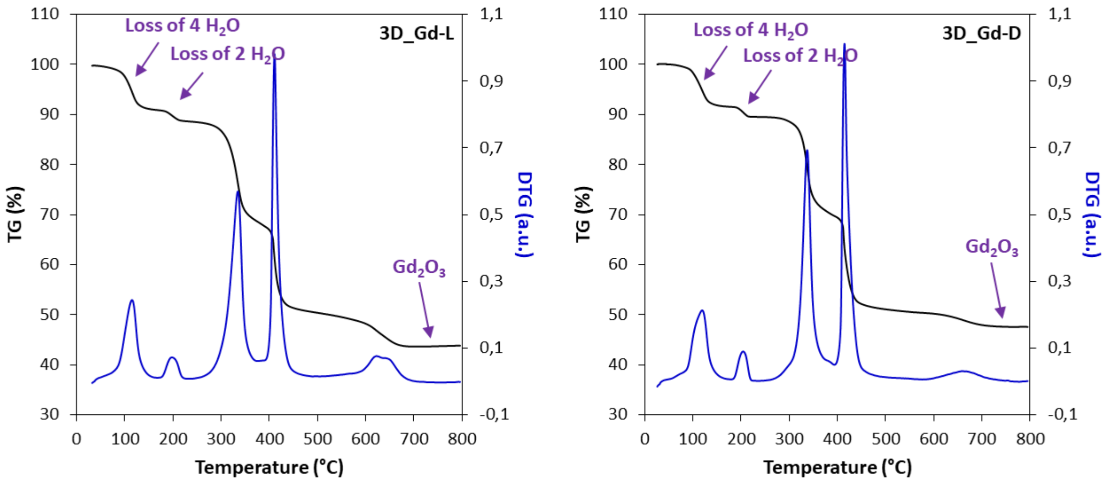

Figure S5. TG/DTG analysis for compounds 3D_Gd-L and 3D_Gd-D.

S11. Thermogravimetric Analysis of chiral 2D MOFs.

The thermogravimetric behaviour of all compounds is very similar given their isostructural nature. The general behaviour accounting for the partially dehydrated $[Ln(\mu\text{-}Htart)_2(H_2O)(OH)]_n$ formula is described. Heating of the sample starts with a short plateau in the TG curve from room temperature up to ca. 70 ºC, after which one coordination water molecule is lost. This dehydration finishes at ca. 190 ºC. Afterward, materials experiment the loss of the coordinated hydroxide, which finishes at ca. 230 ºC. The dehydration process of these compounds yields an unstable phase that is collapsed by two main exothermic processes which involve to the decomposition of tartrate ligands, leading to the corresponding Ln_2O_3 as a final residue.

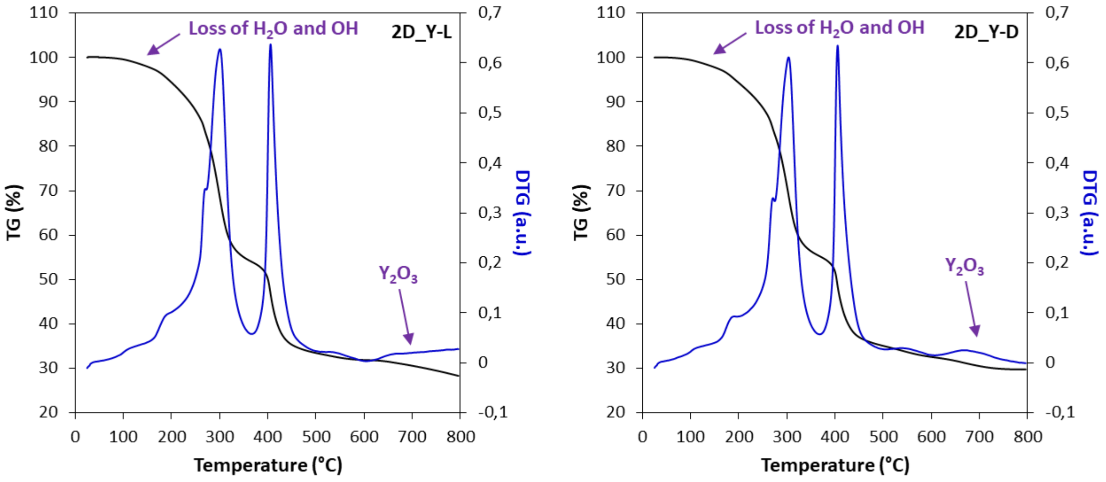

Figure S6. TG/DTG analysis for compounds 2D_Y-L and 2D_Y-D.

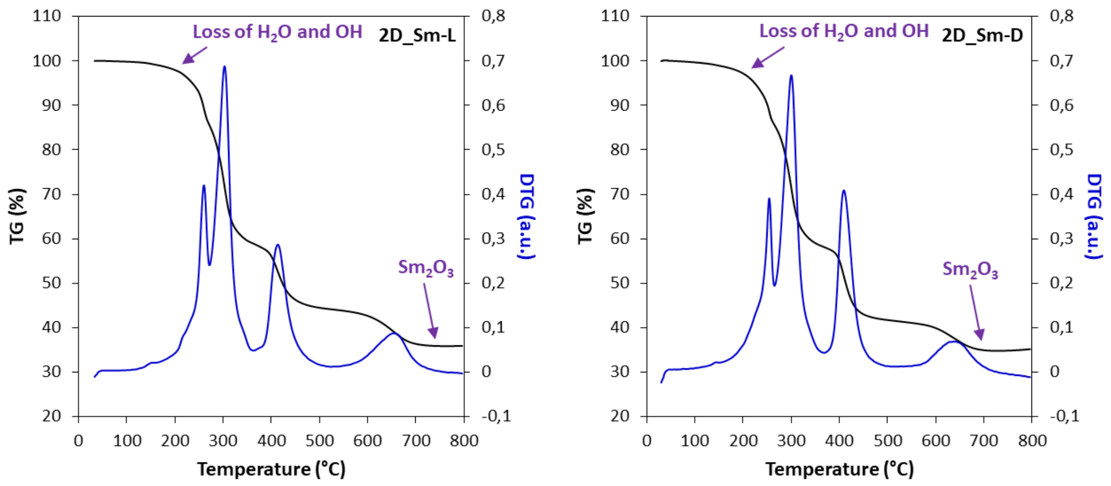

Figure S7. TG/DTG analysis for compounds 2D_Sm-L and 2D_Sm-D.

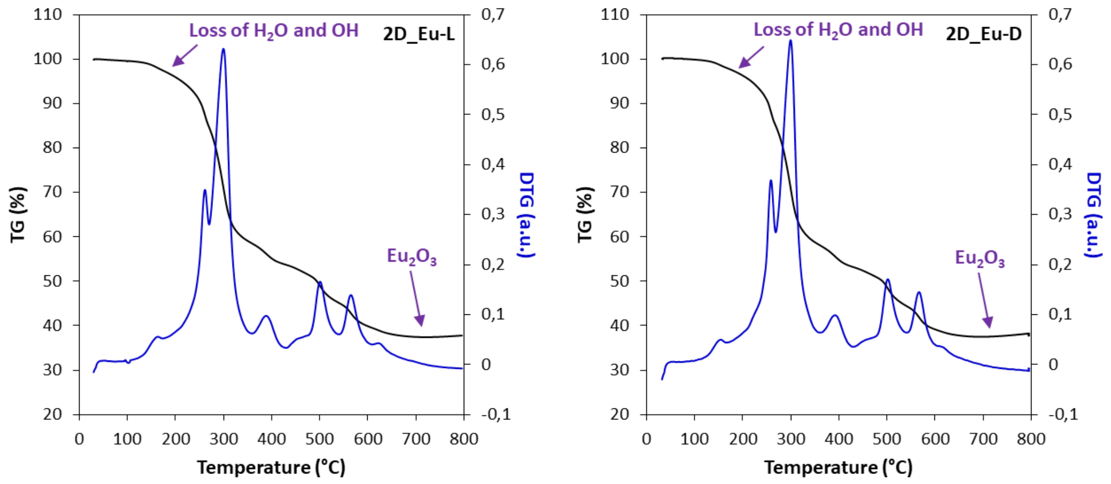

Figure S8. TG/DTG analysis for compounds 2D_Eu-L and 2D_Eu-D.

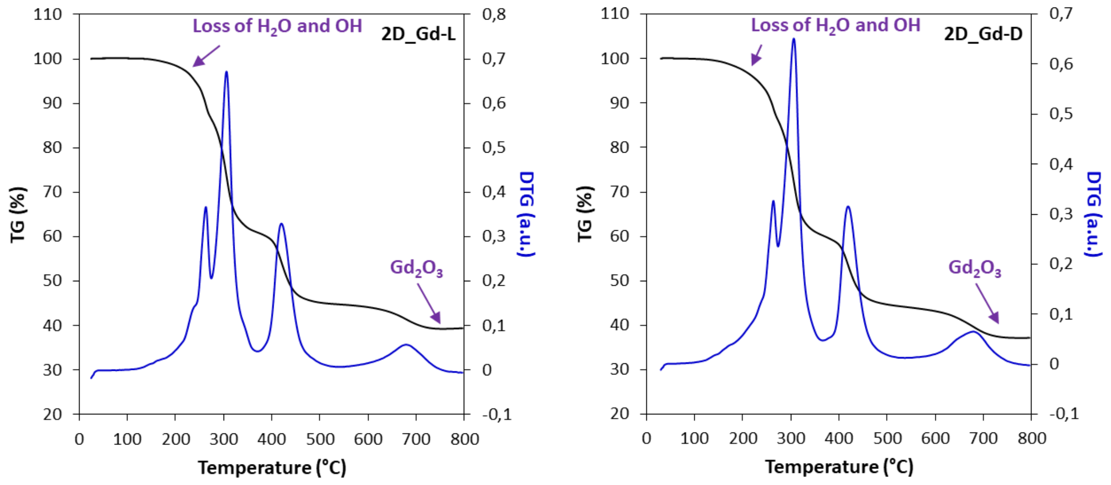

Figure S9. TG/DTG analysis for compounds 2D_Gd-L and 2D_Gd-D.

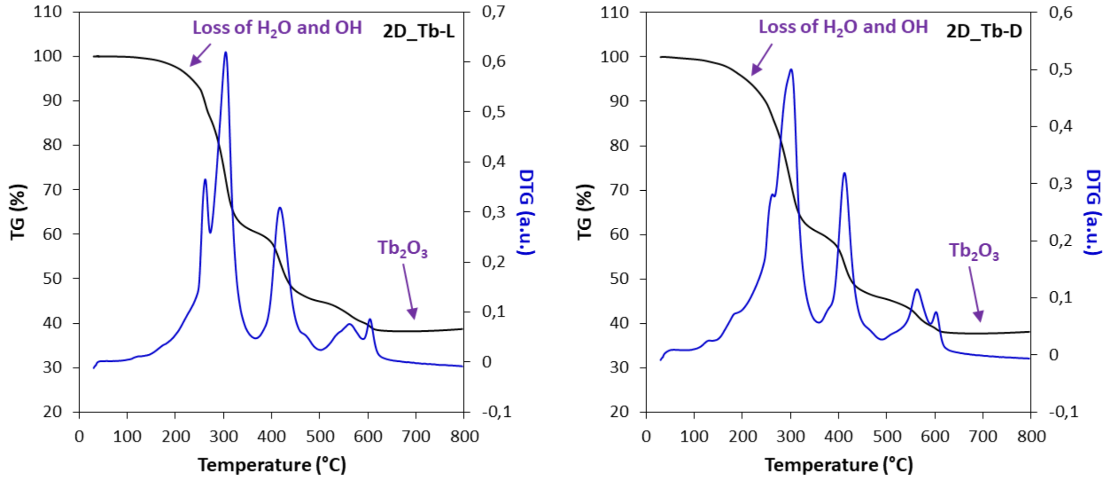

Figure S10. TG/DTG analysis for compounds 2D_Tb-L and 2D_Tb-D.

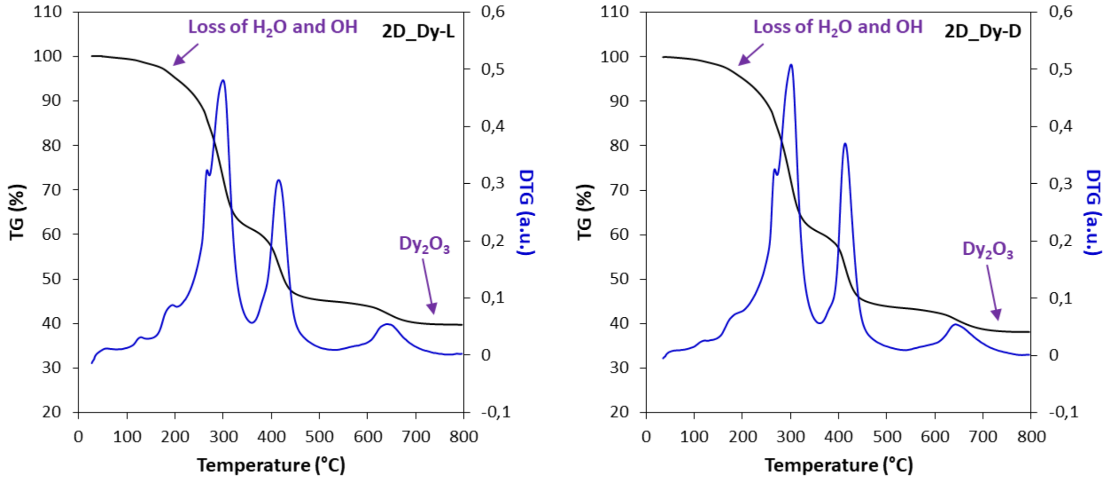

Figure S11. TG/DTG analysis for compounds 2D_Dy-L and 2D_Dy-D.

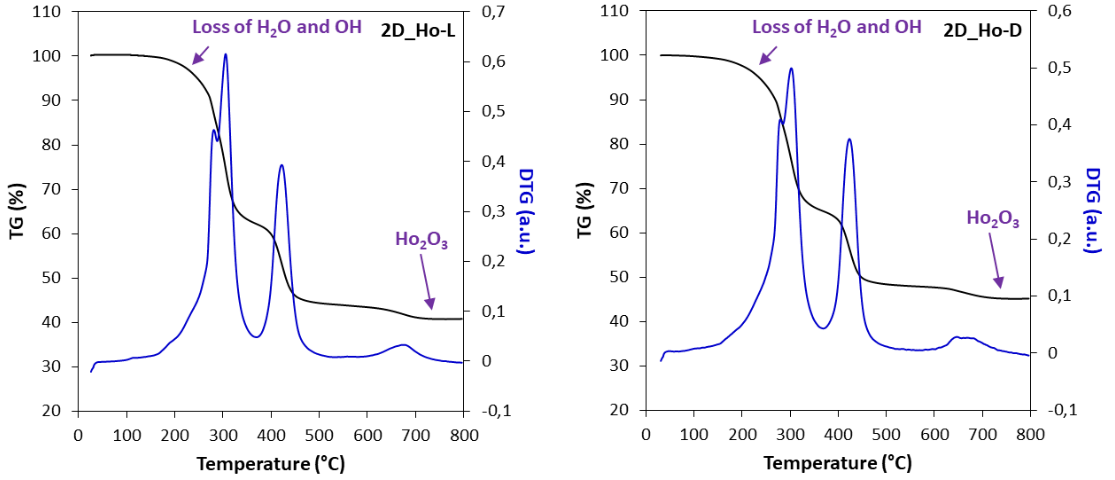

Figure S12. TG/DTG analysis for compounds 2D_Ho-L and 2D_Ho-D.

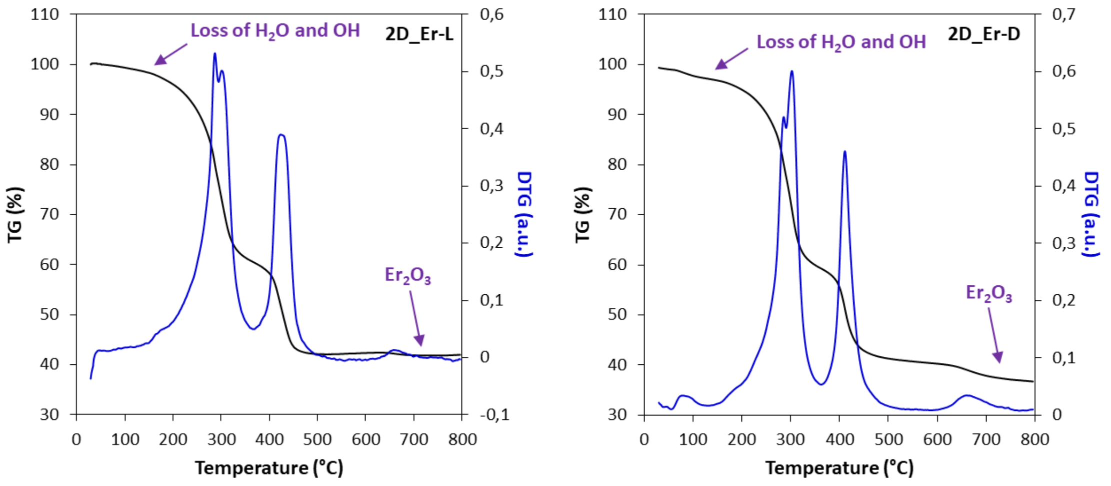

Figure S13. TG/DTG analysis for compounds 2D_Er-L and 2D_Er-D.

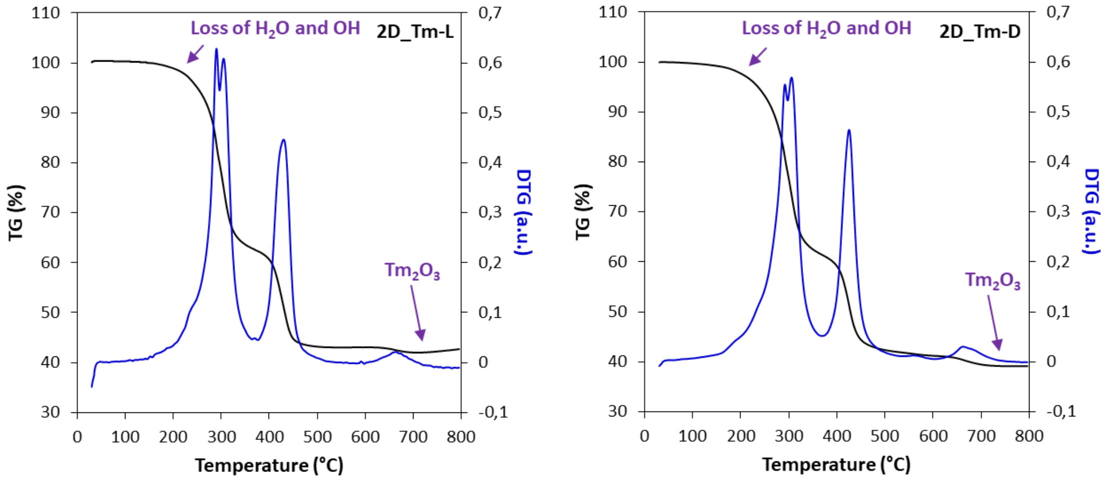

Figure S14. TG/DTG analysis for compounds 2D_Tm-L and 2D_Tm-D.

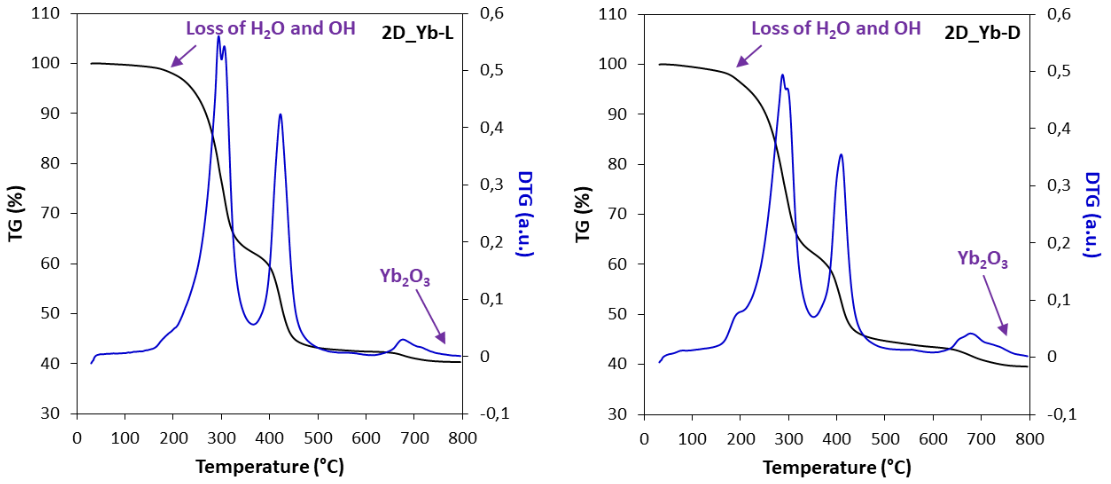

Figure S15. TG/DTG analysis for compounds 2D_Yb-L and 2D_Yb-D.

S12. Powder X-ray Diffraction Analysis of chiral 3D MOFs.

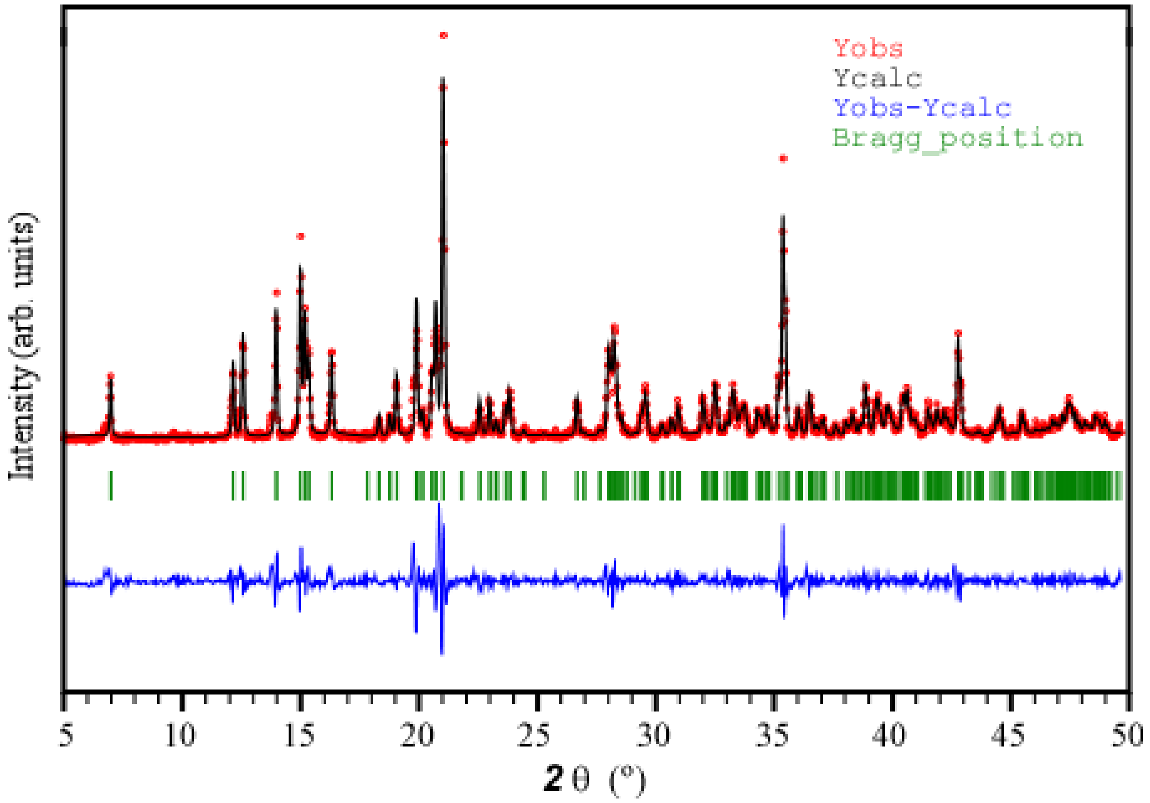

Figure S16. Pattern-matching analysis of polycrystalline sample of compound 3D_Sm-L.

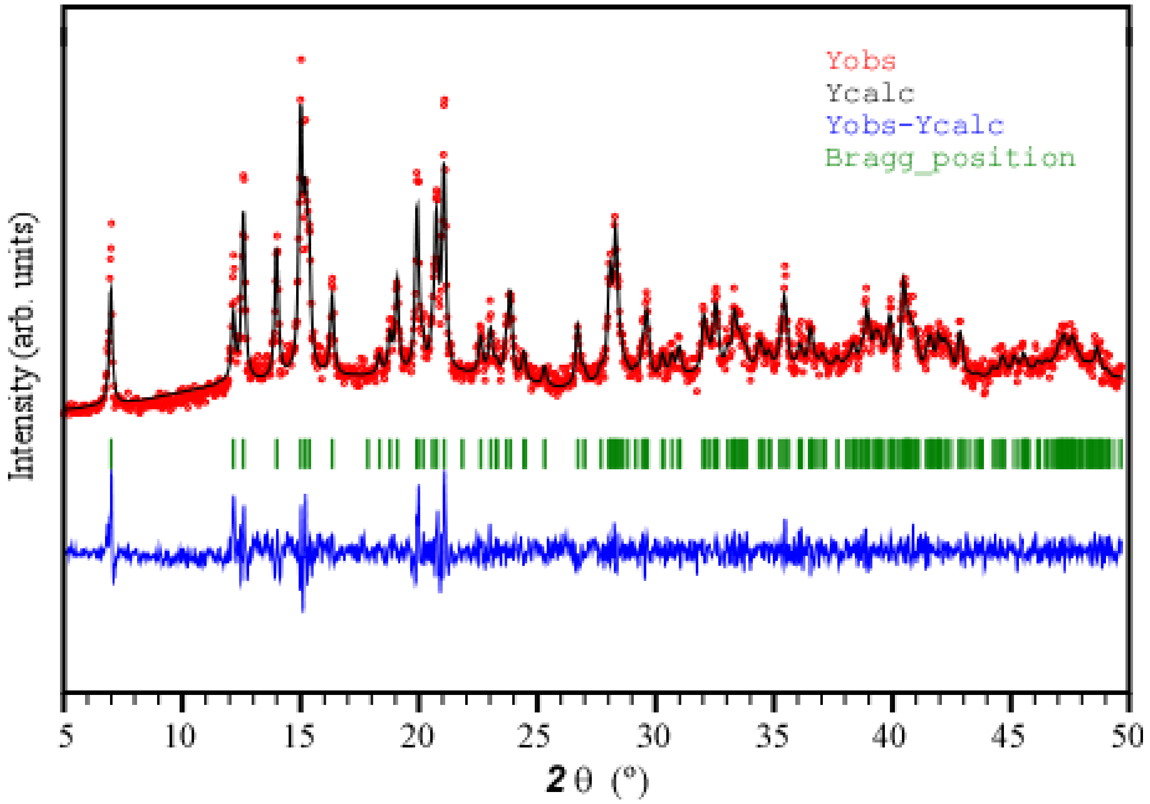

Figure S17. Pattern-matching analysis of polycrystalline sample of compound 3D_Sm-D.

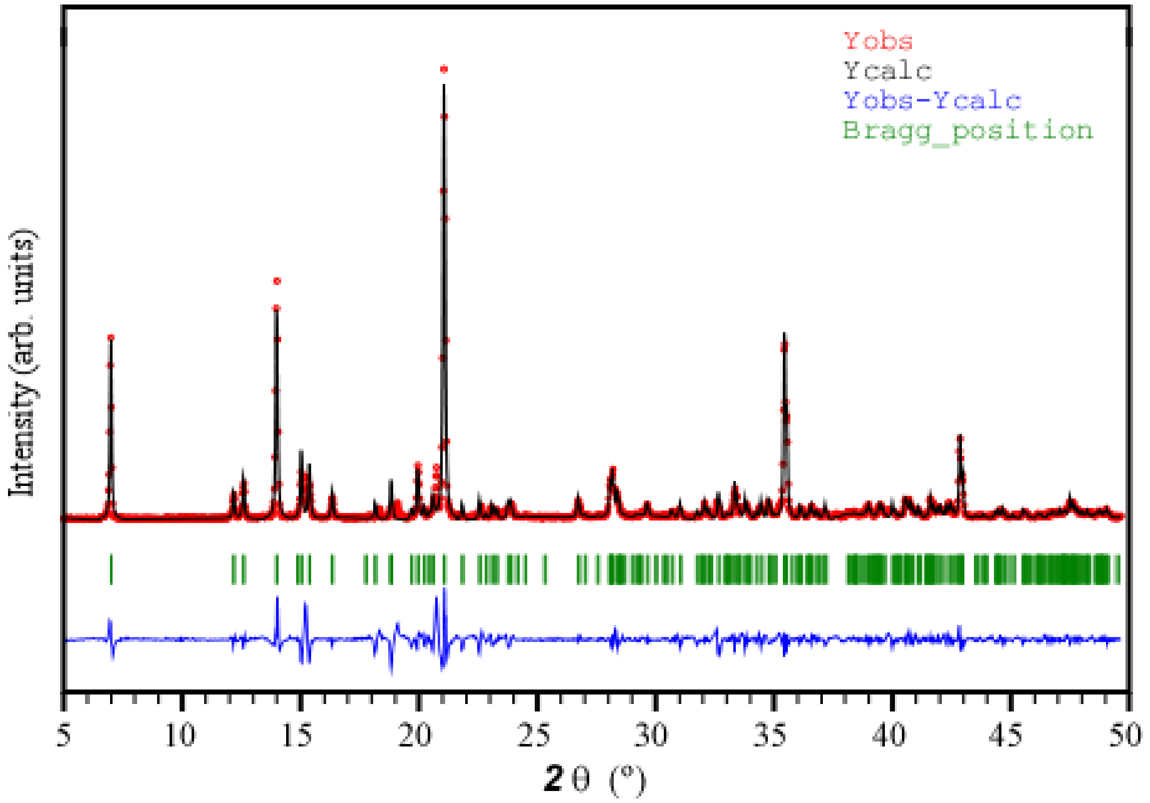

Figure S18. Pattern-matching analysis of polycrystalline sample of compound 3D_Eu-L.

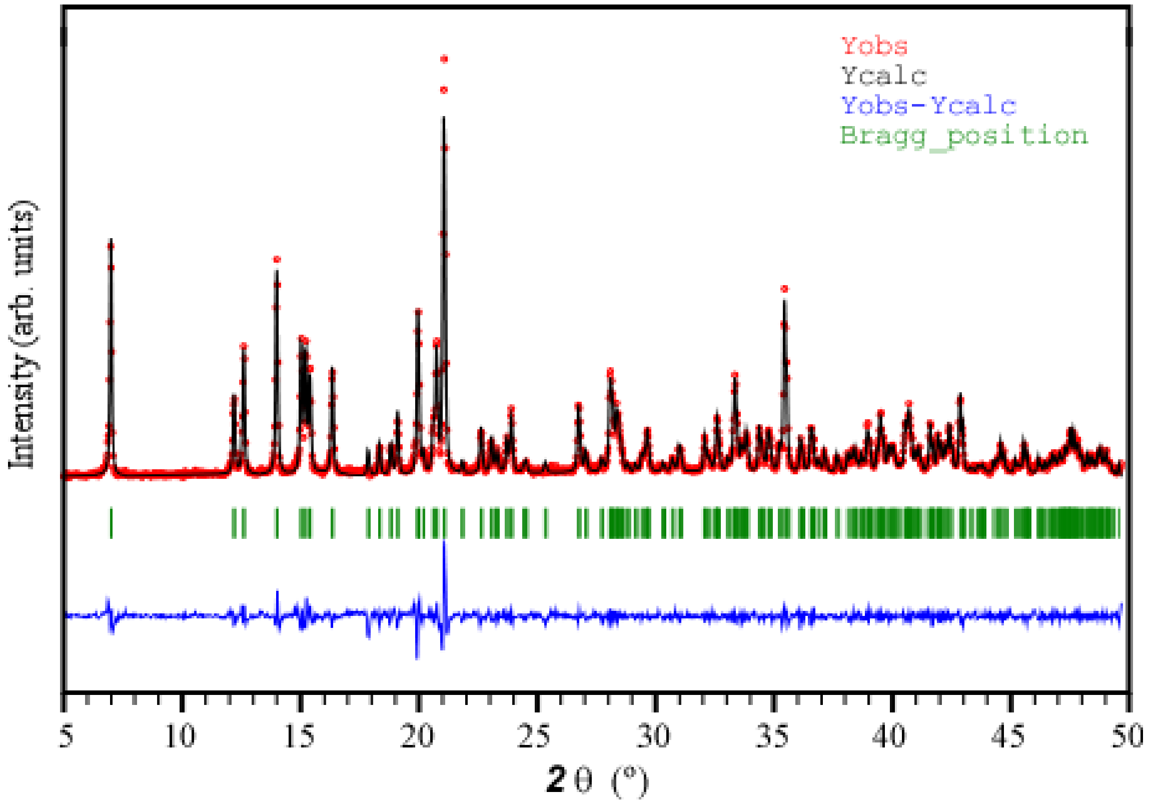

Figure S19. Pattern-matching analysis of polycrystalline sample of compound 3D_Eu-D.

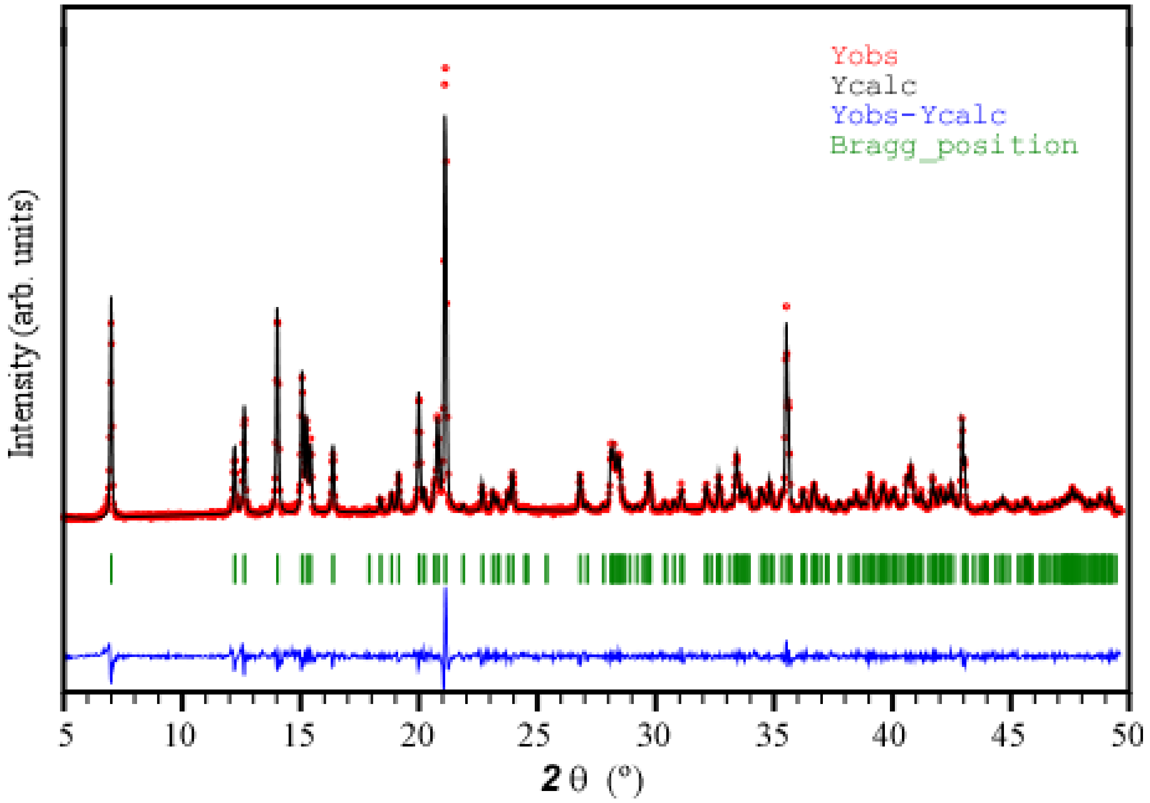

Figure S20. Pattern-matching analysis of polycrystalline sample of compound 3D_Gd-L.

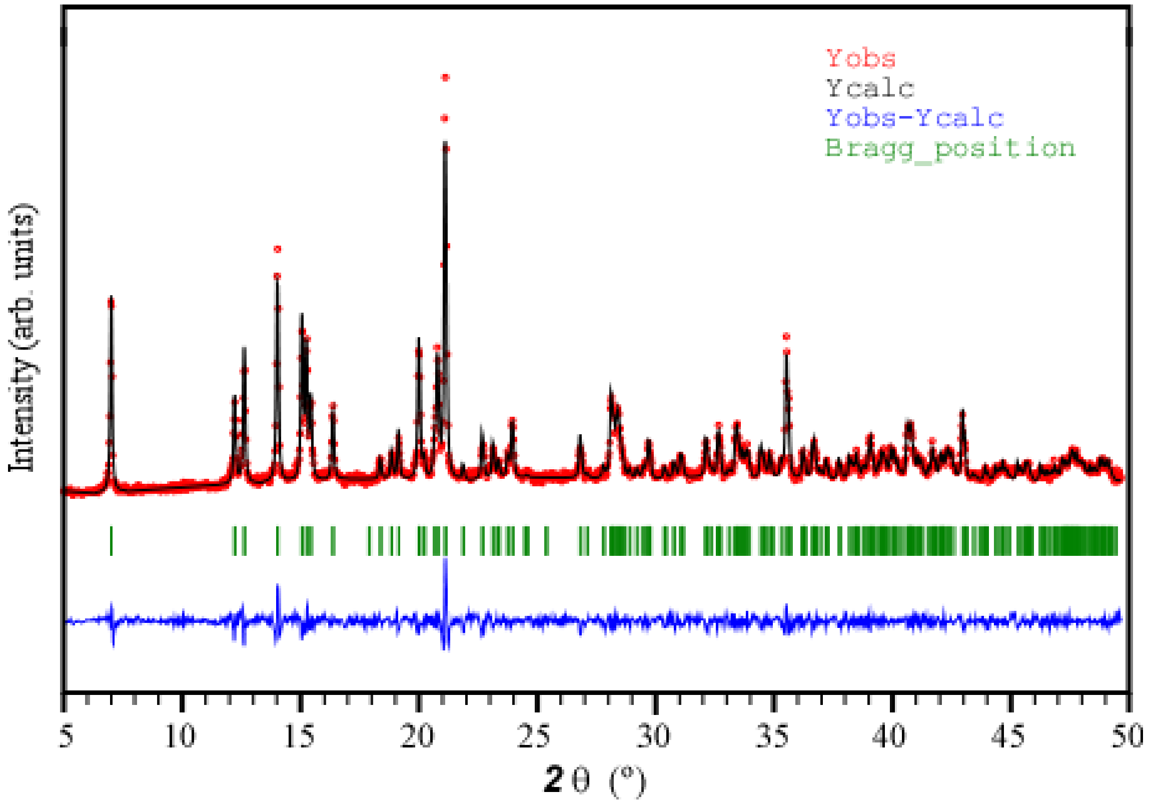

Figure S21. Pattern-matching analysis of polycrystalline sample of compound 3D_Gd-D.

S13. Powder X-ray Diffraction Analysis of chiral 2D MOFs.

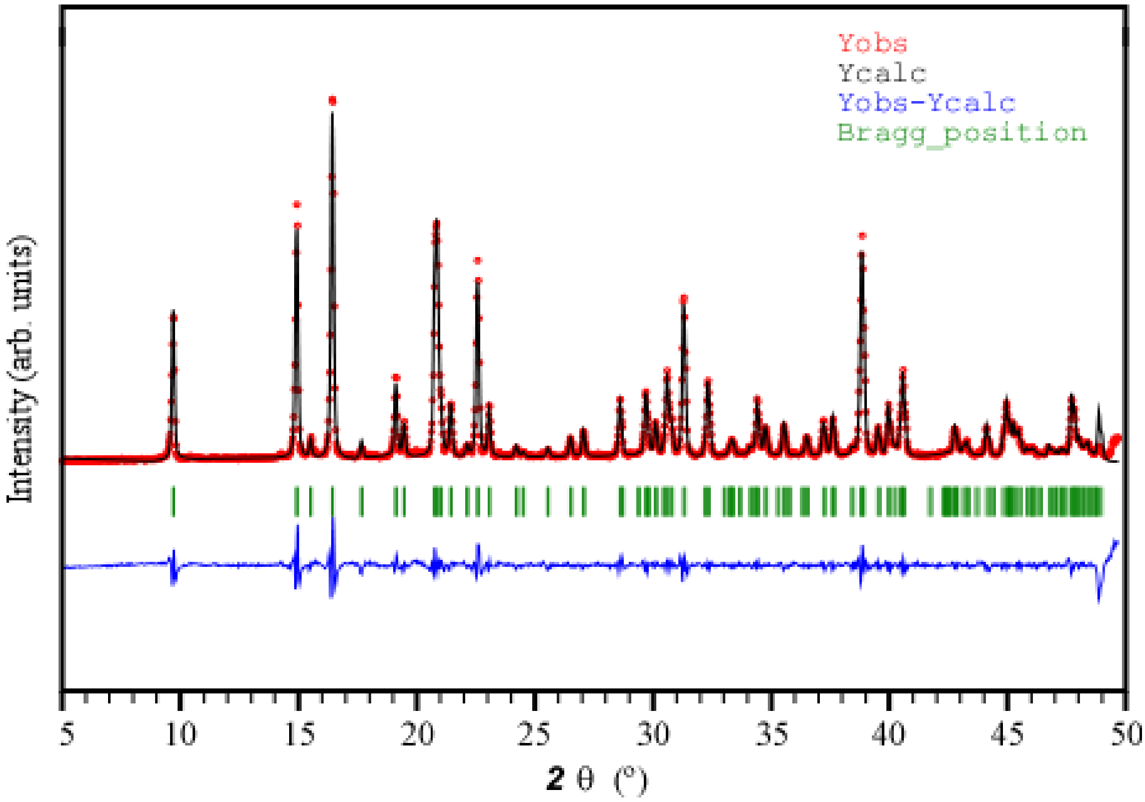

Figure S22. Pattern-matching analysis of polycrystalline sample of compound 2D_Y-L.

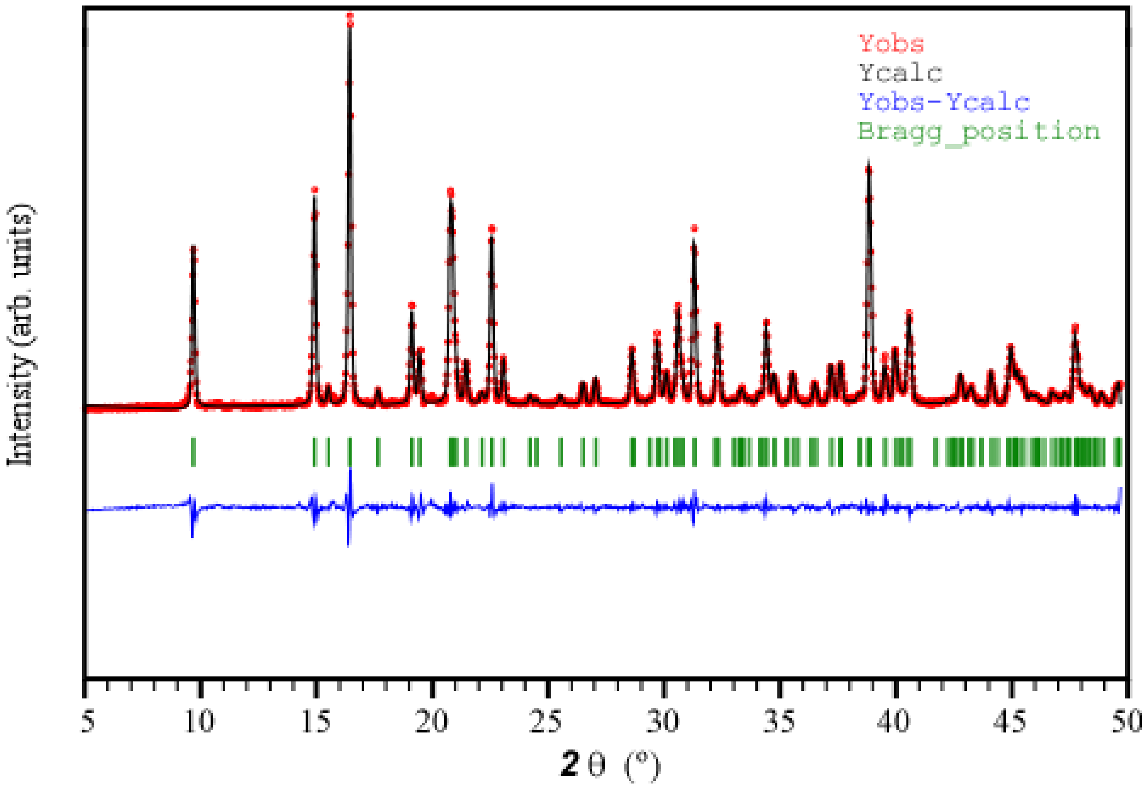

Figure S23. Pattern-matching analysis of polycrystalline sample of compound 2D_Y-D.

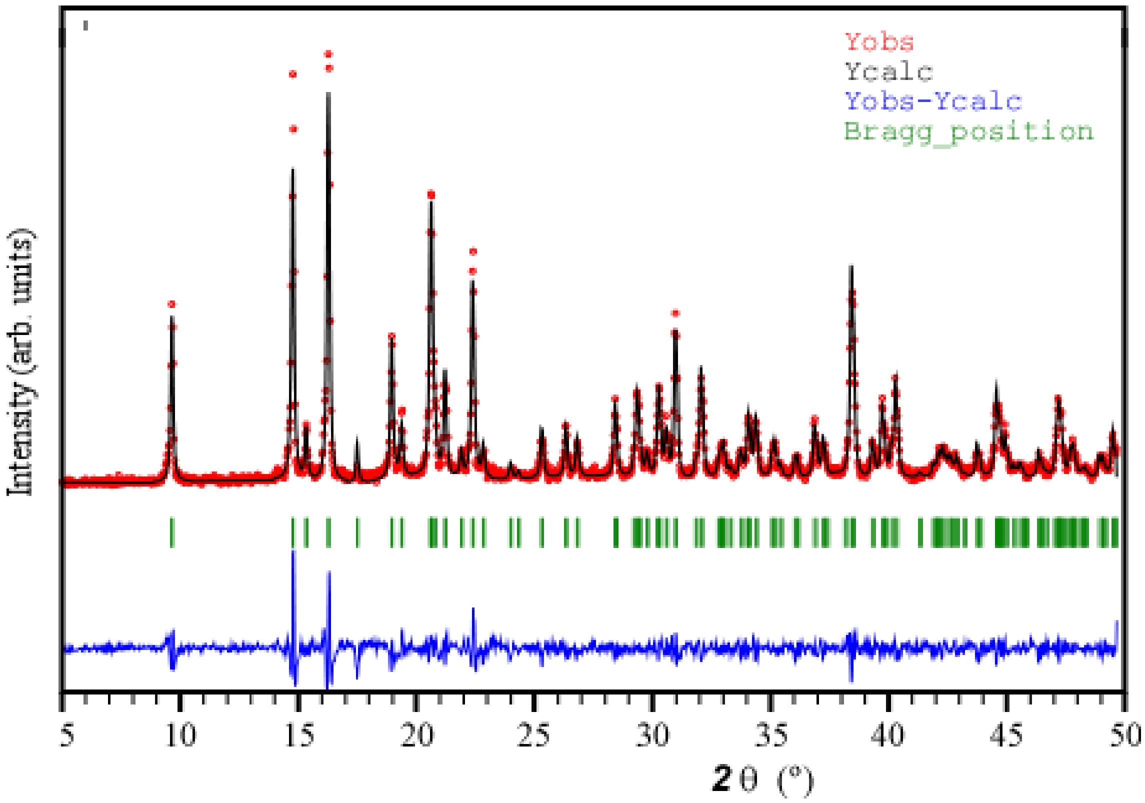

Figure S24. Pattern-matching analysis of polycrystalline sample of compound 2D_Sm-L.

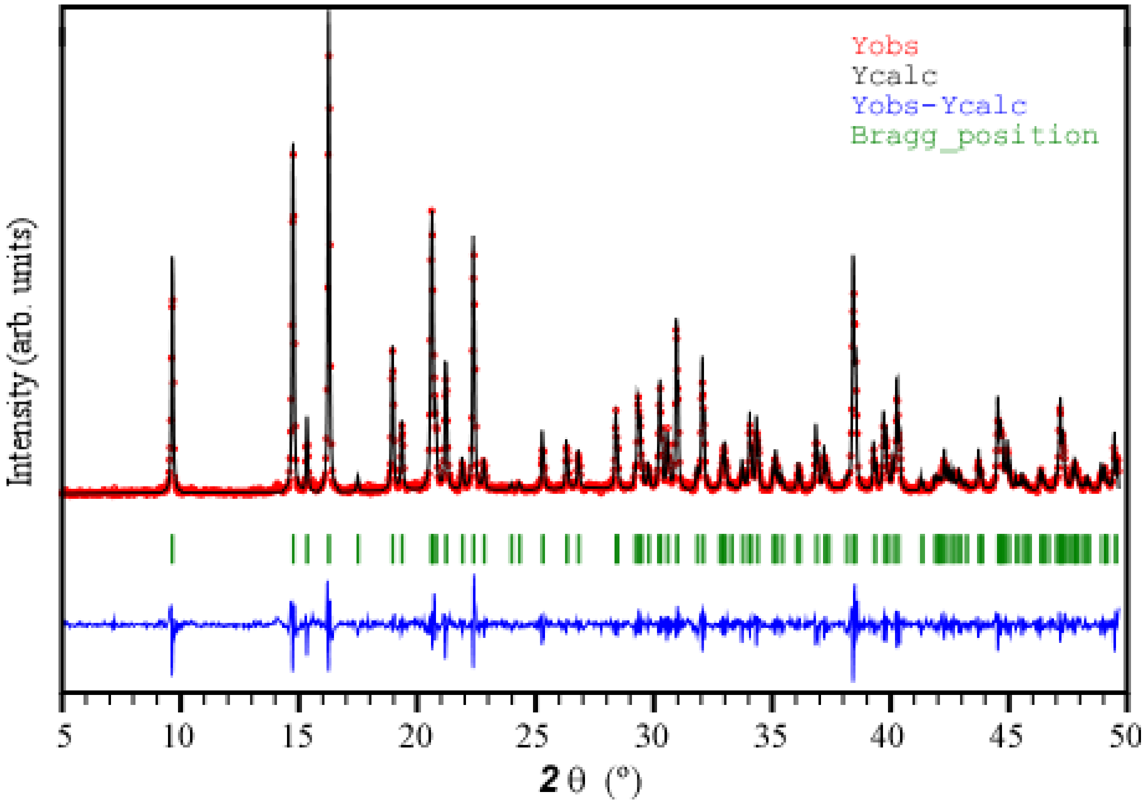

Figure S25. Pattern-matching analysis of polycrystalline sample of compound 2D_Sm-D.

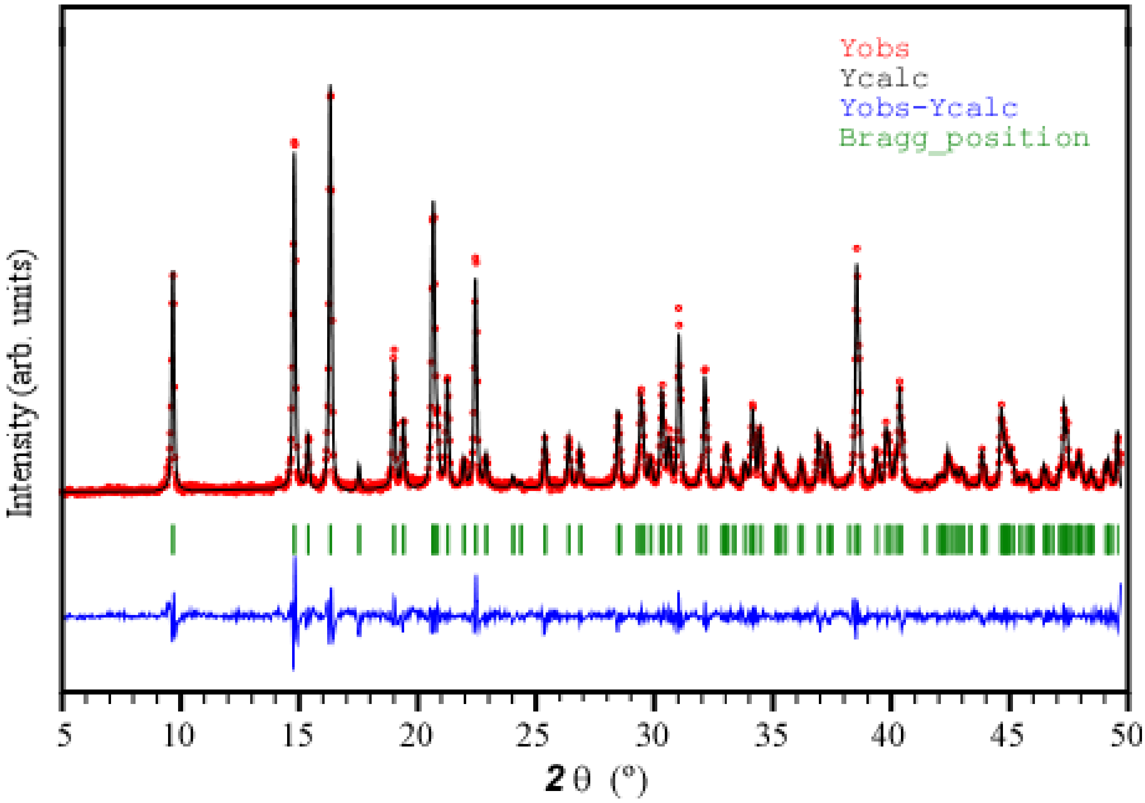

Figure S26. Pattern-matching analysis of polycrystalline sample of compound 2D_Eu-L.

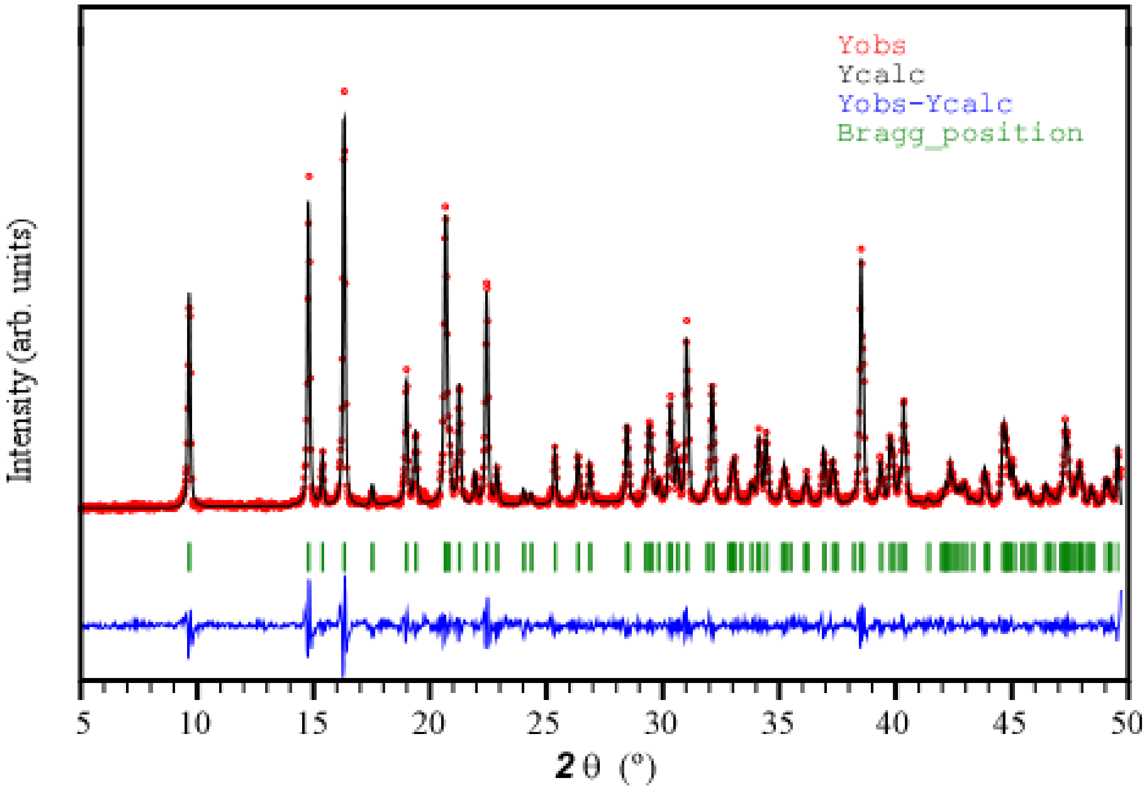

Figure S27. Pattern-matching analysis of polycrystalline sample of compound 2D_Eu-D.

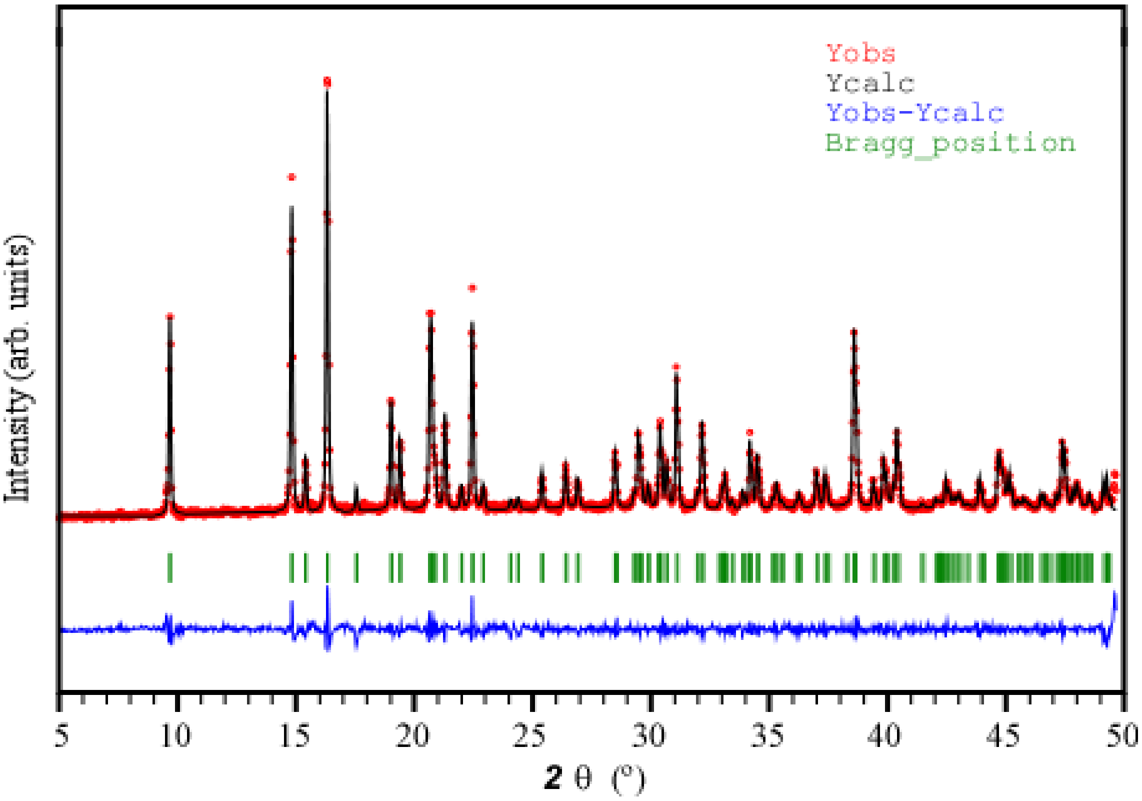

Figure S28. Pattern-matching analysis of polycrystalline sample of compound **2D_Gd-L**.

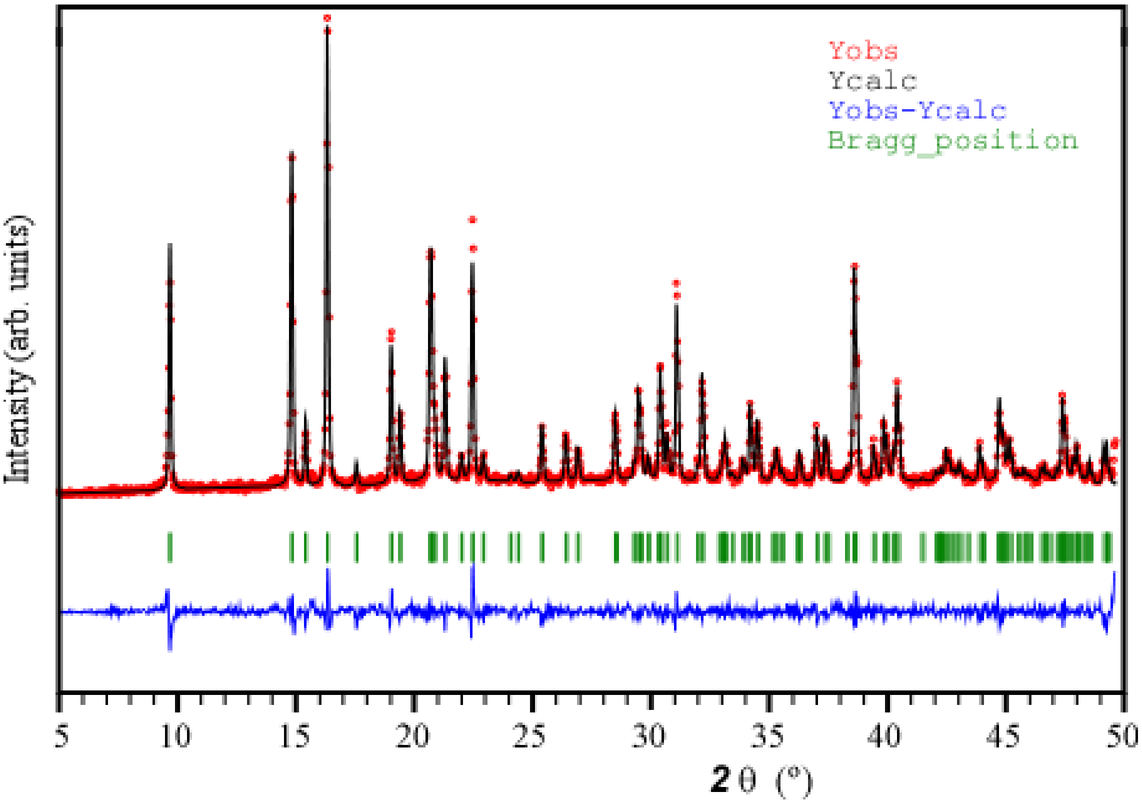

Figure S29. Pattern-matching analysis of polycrystalline sample of compound 2D_Gd-D.

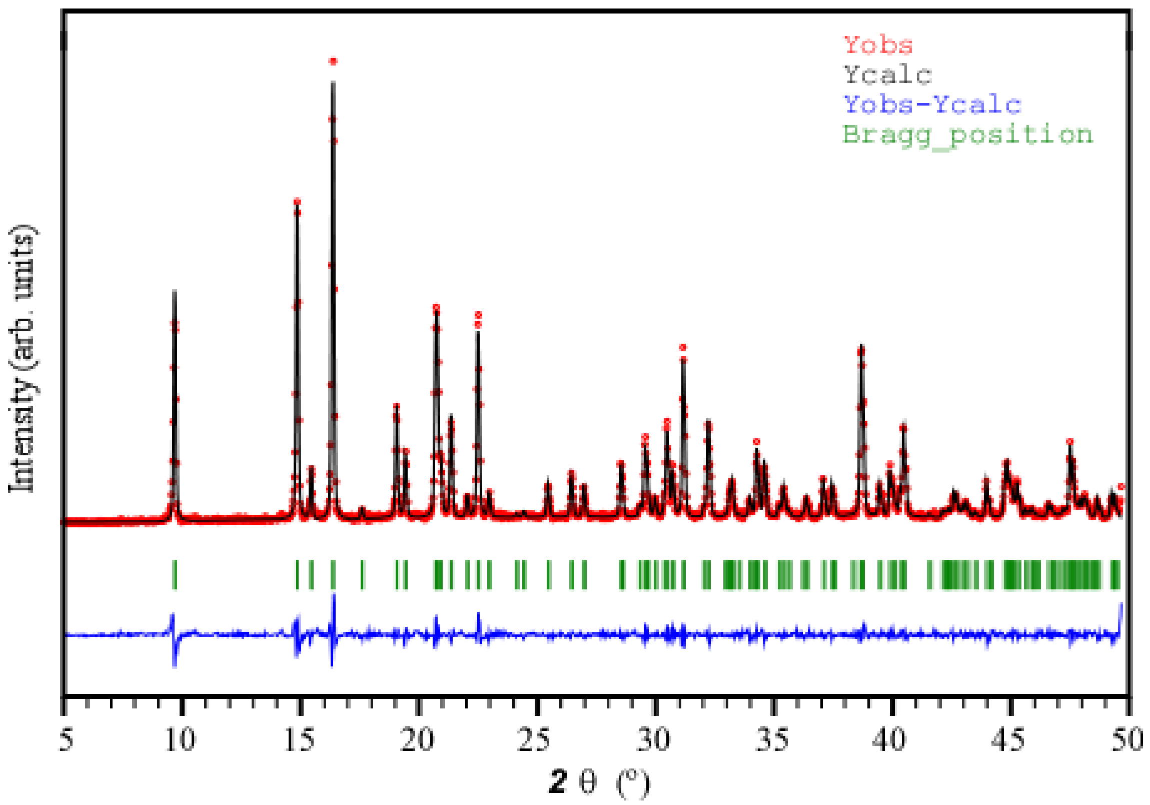

Figure S30. Pattern-matching analysis of polycrystalline sample of compound 2D_Tb-L.

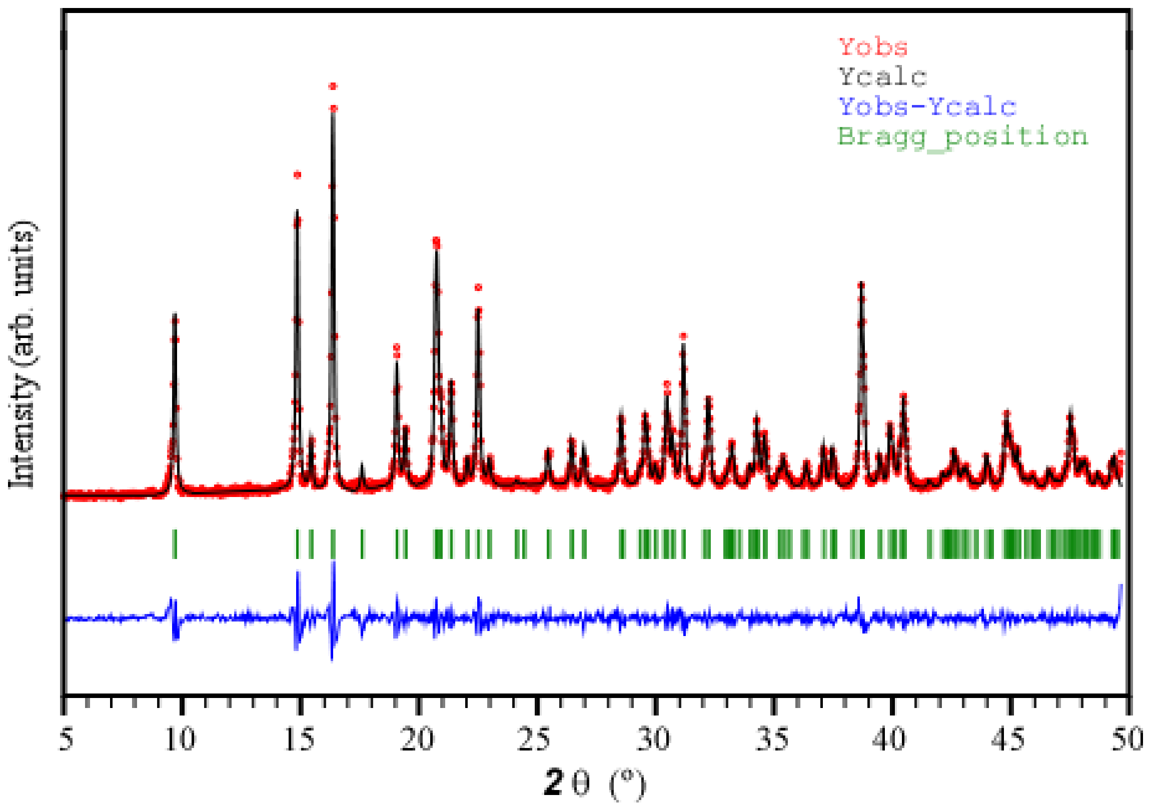

Figure S31. Pattern-matching analysis of polycrystalline sample of compound 2D_Tb-D.

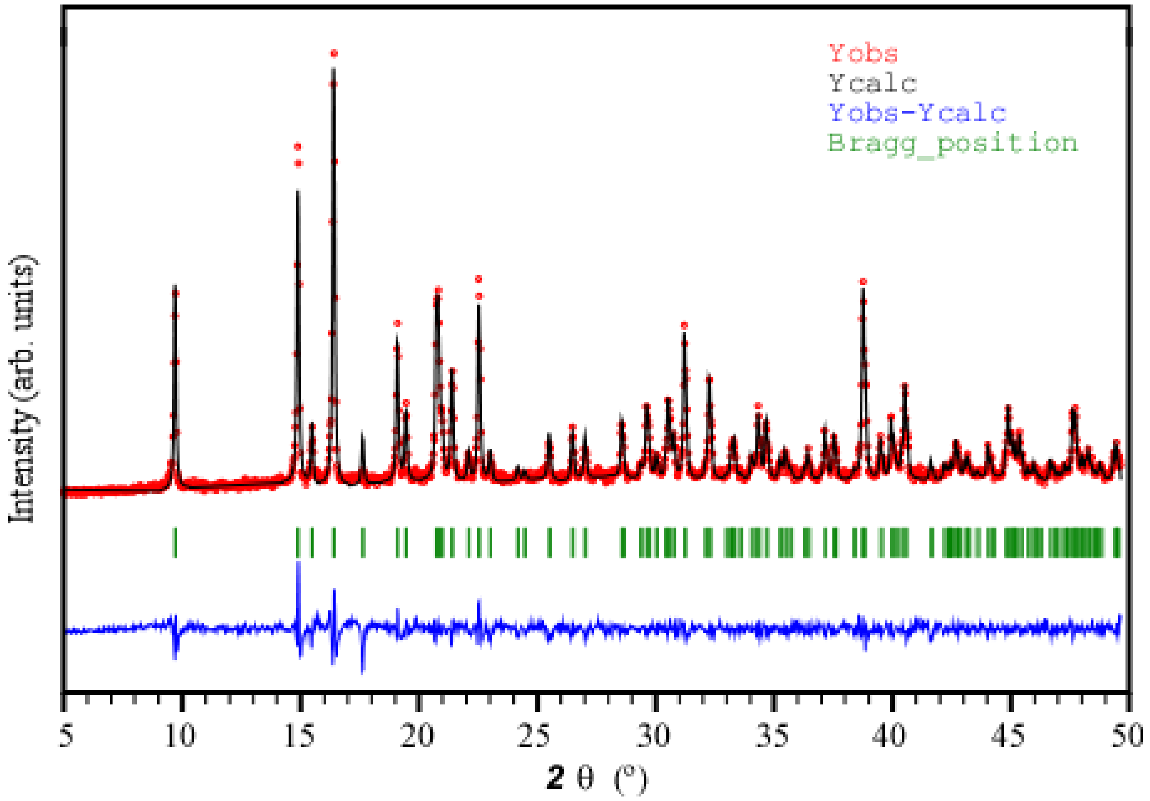

Figure S32. Pattern-matching analysis of polycrystalline sample of compound 2D_Dy-L.

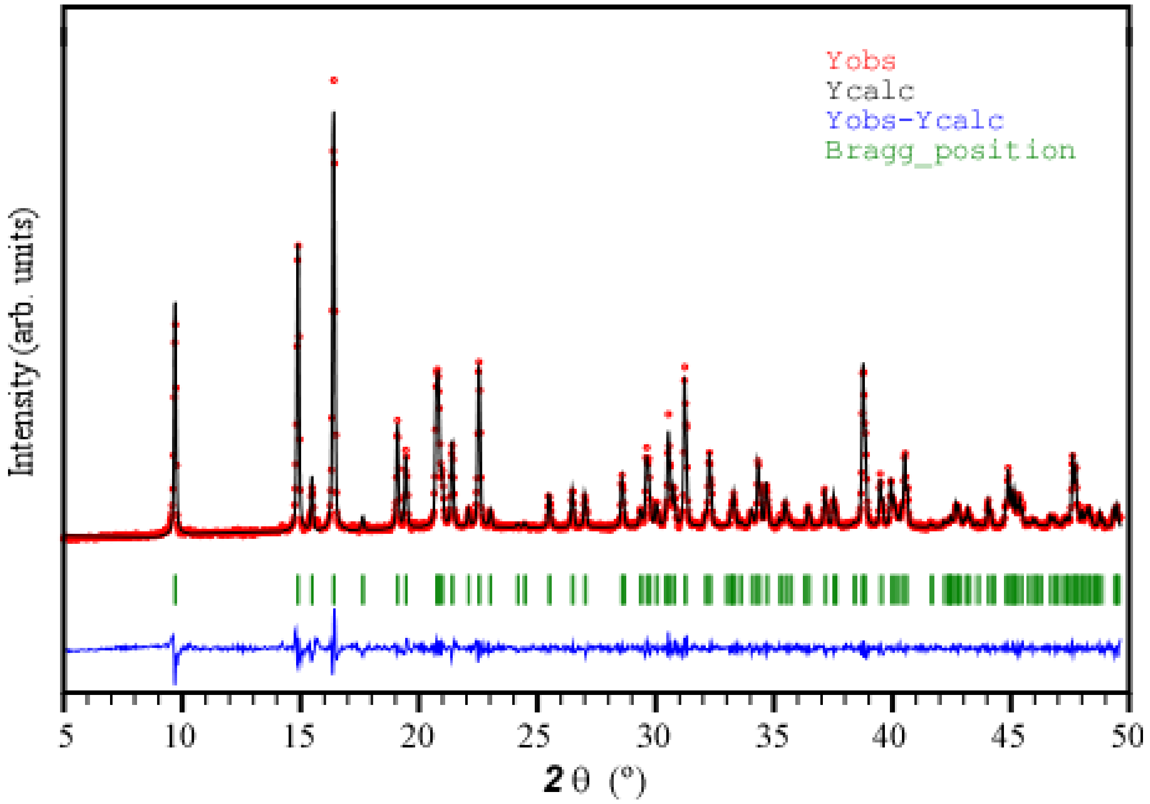

Figure S33. Pattern-matching analysis of polycrystalline sample of compound **2D_Dy-D**.

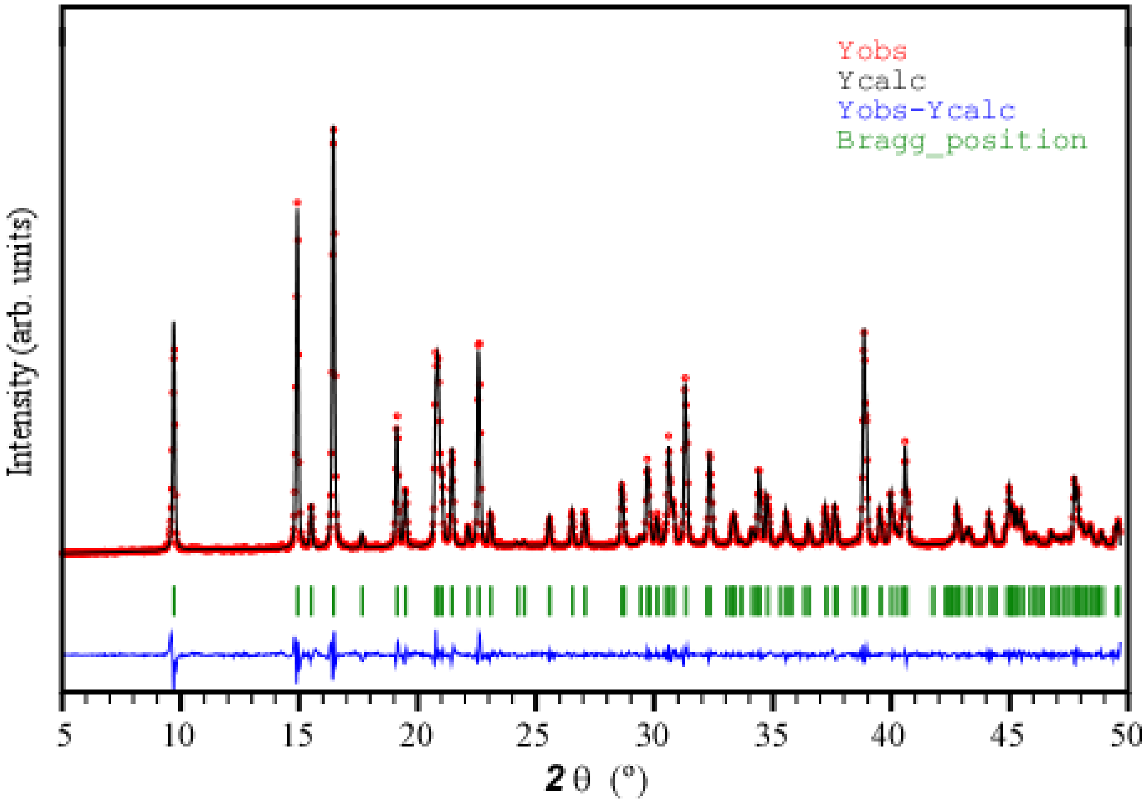

Figure S34. Pattern-matching analysis of polycrystalline sample of compound **2D_Ho-L**.

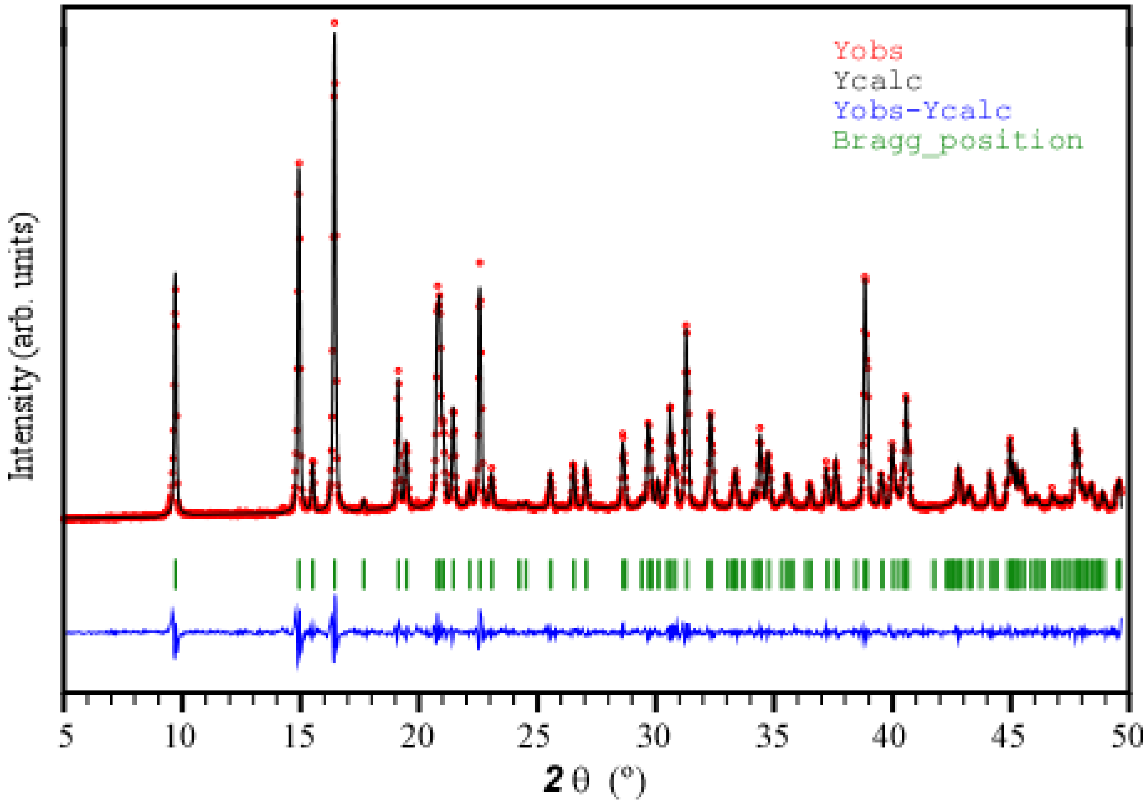

Figure S35. Pattern-matching analysis of polycrystalline sample of compound 2D_Ho-D.

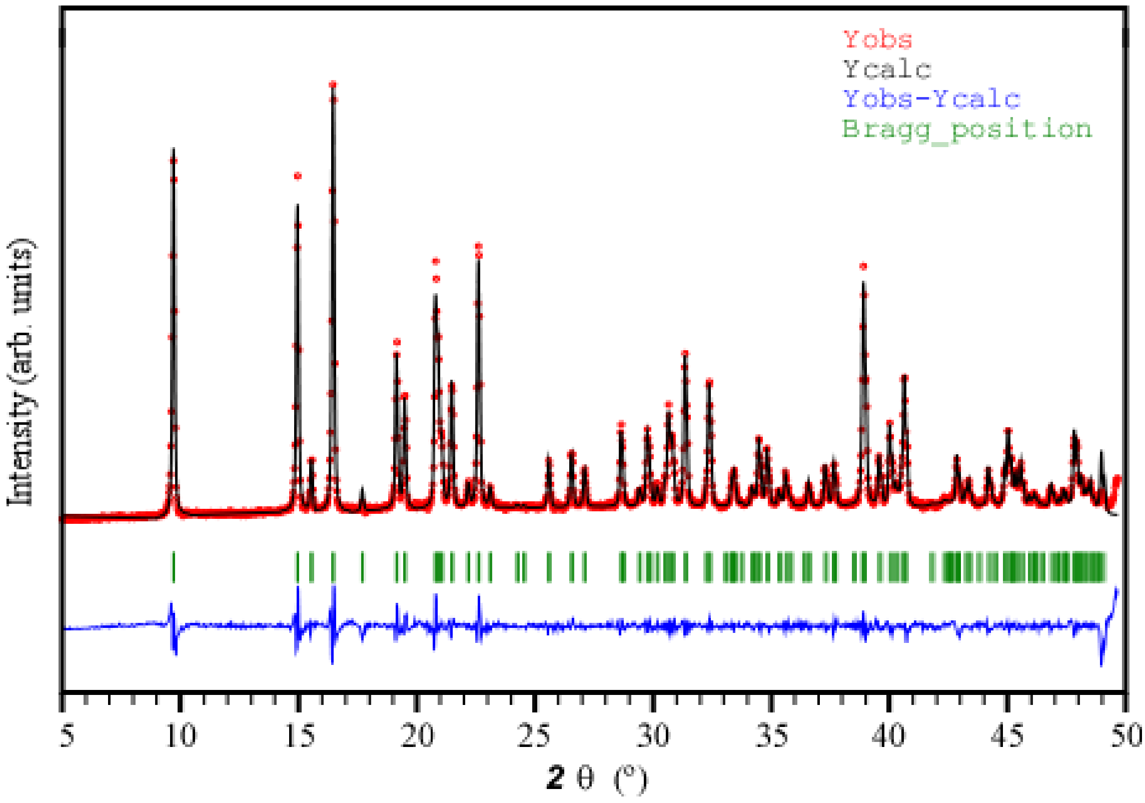

Figure S36. Pattern-matching analysis of polycrystalline sample of compound **2D_Er-L**.

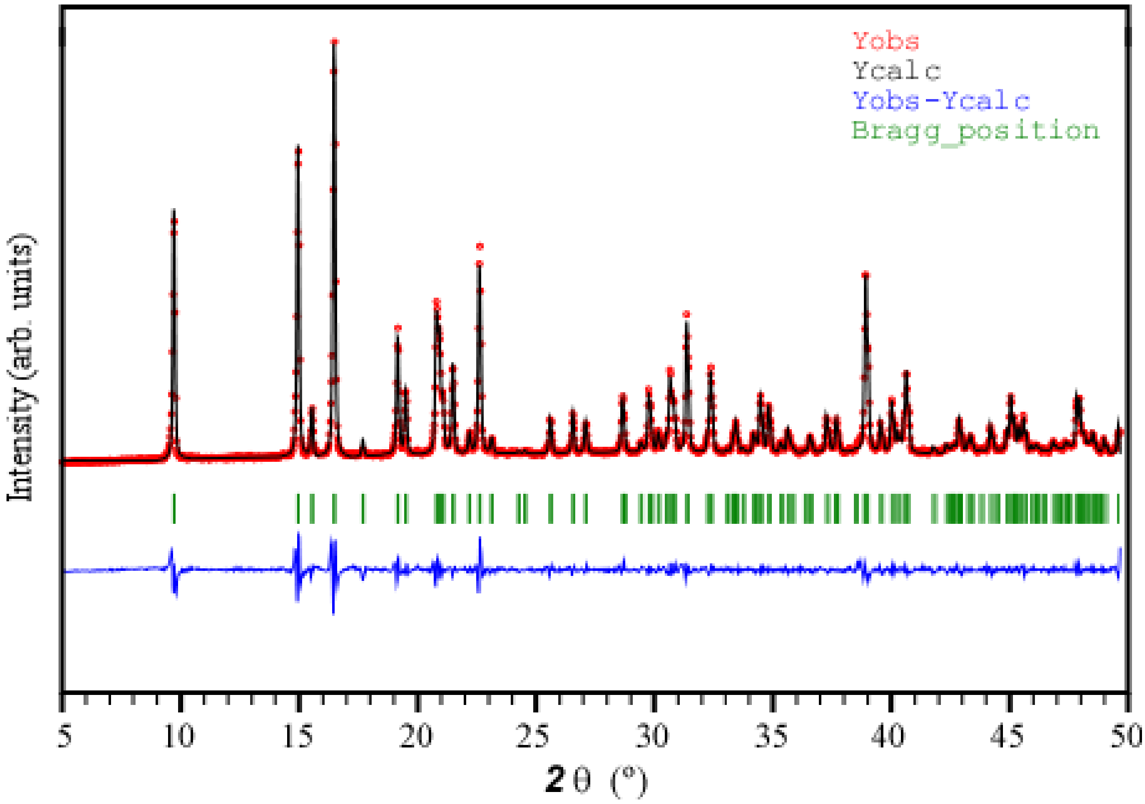

Figure S37. Pattern-matching analysis of polycrystalline sample of compound 2D_Er-D.

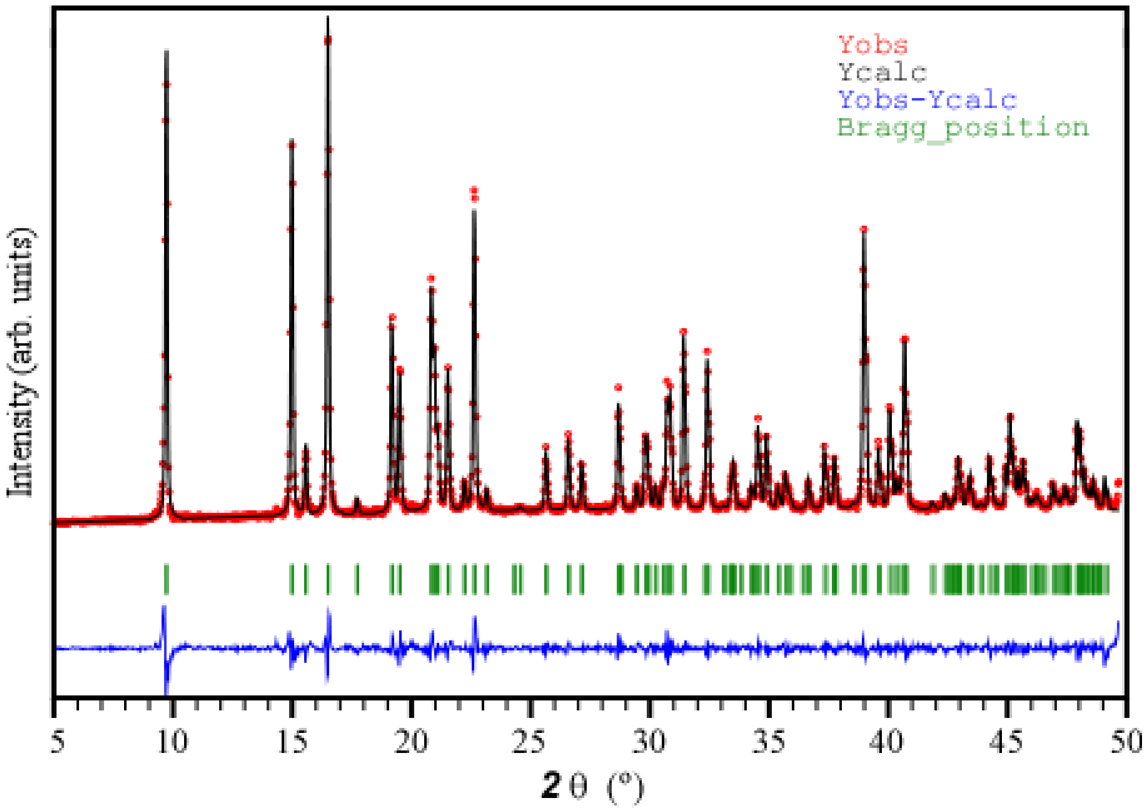

Figure S38. Pattern-matching analysis of polycrystalline sample of compound 2D_Tm-L.

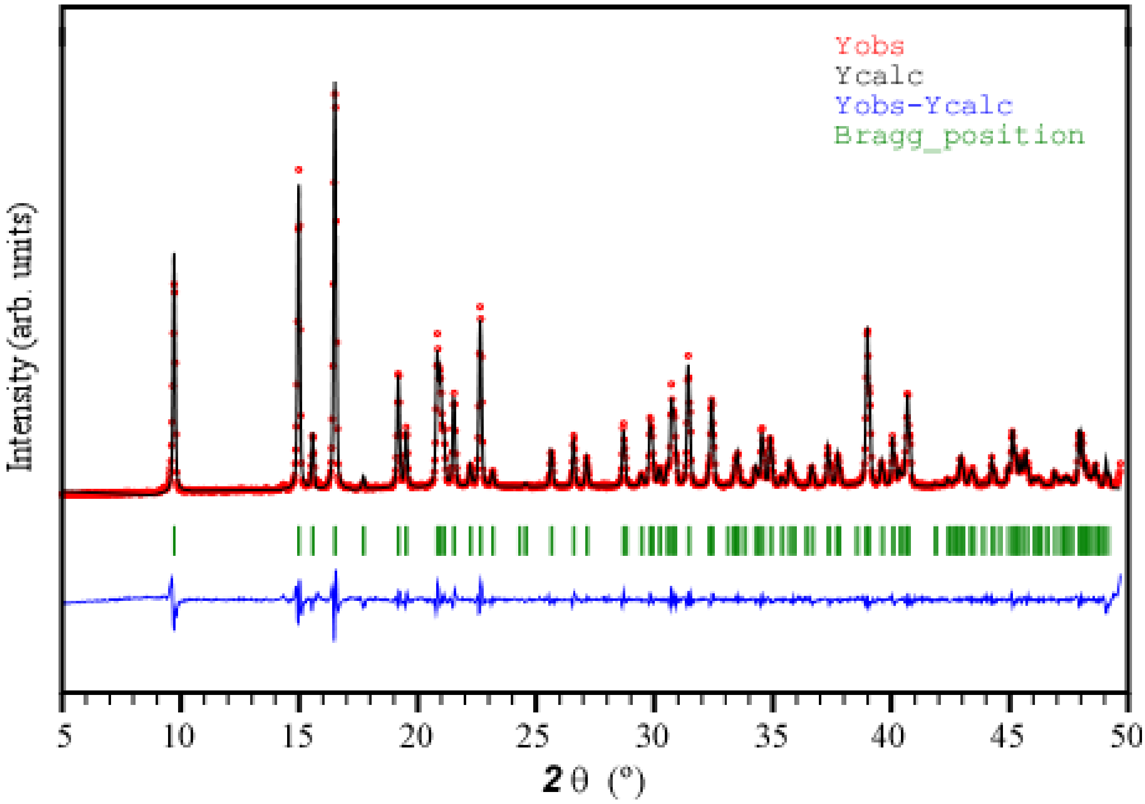

Figure S39. Pattern-matching analysis of polycrystalline sample of compound 2D_Tm-D.

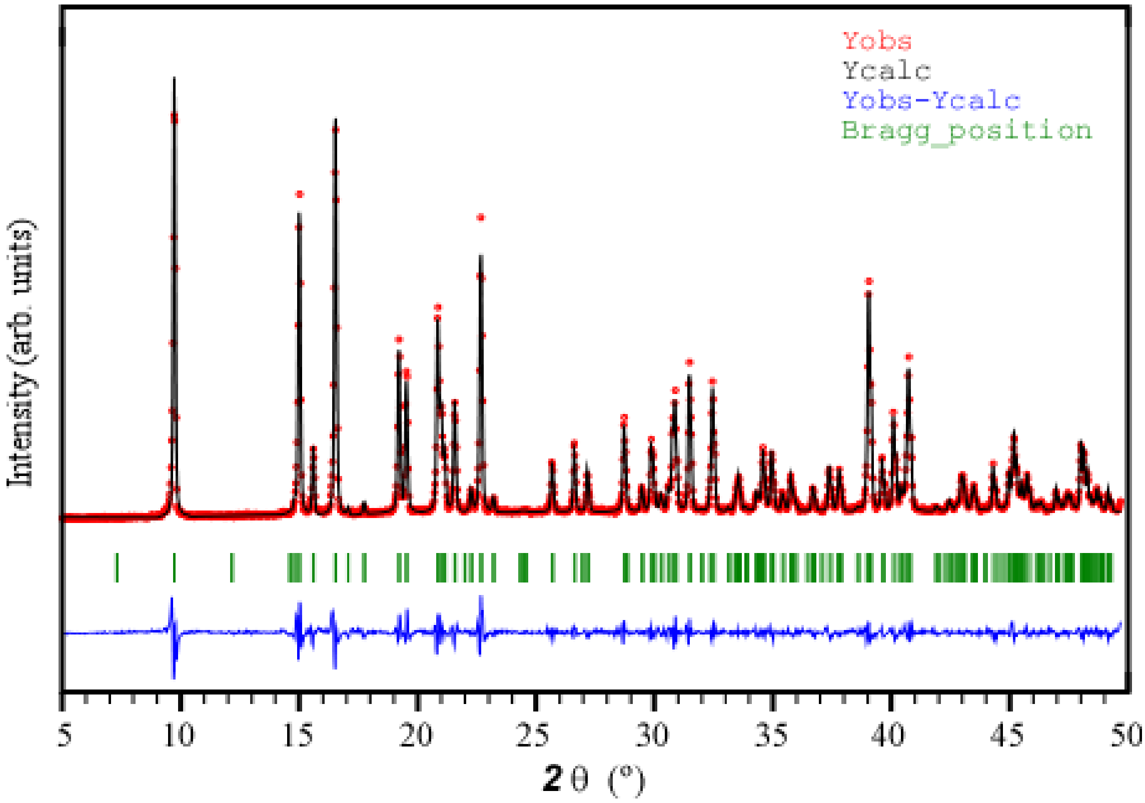

Figure S40. Pattern-matching analysis of polycrystalline sample of compound 2D_Yb-L.

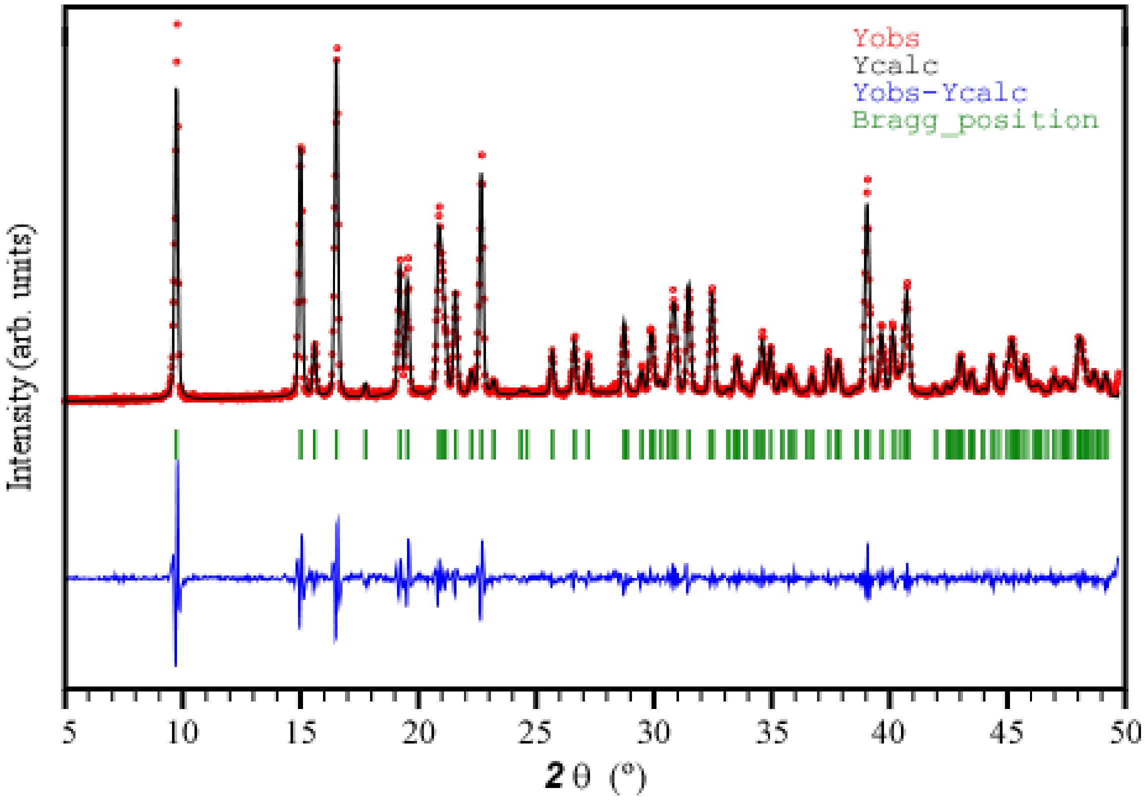

Figure S41. Pattern-matching analysis of polycrystalline sample of compound 2D_Yb-D.

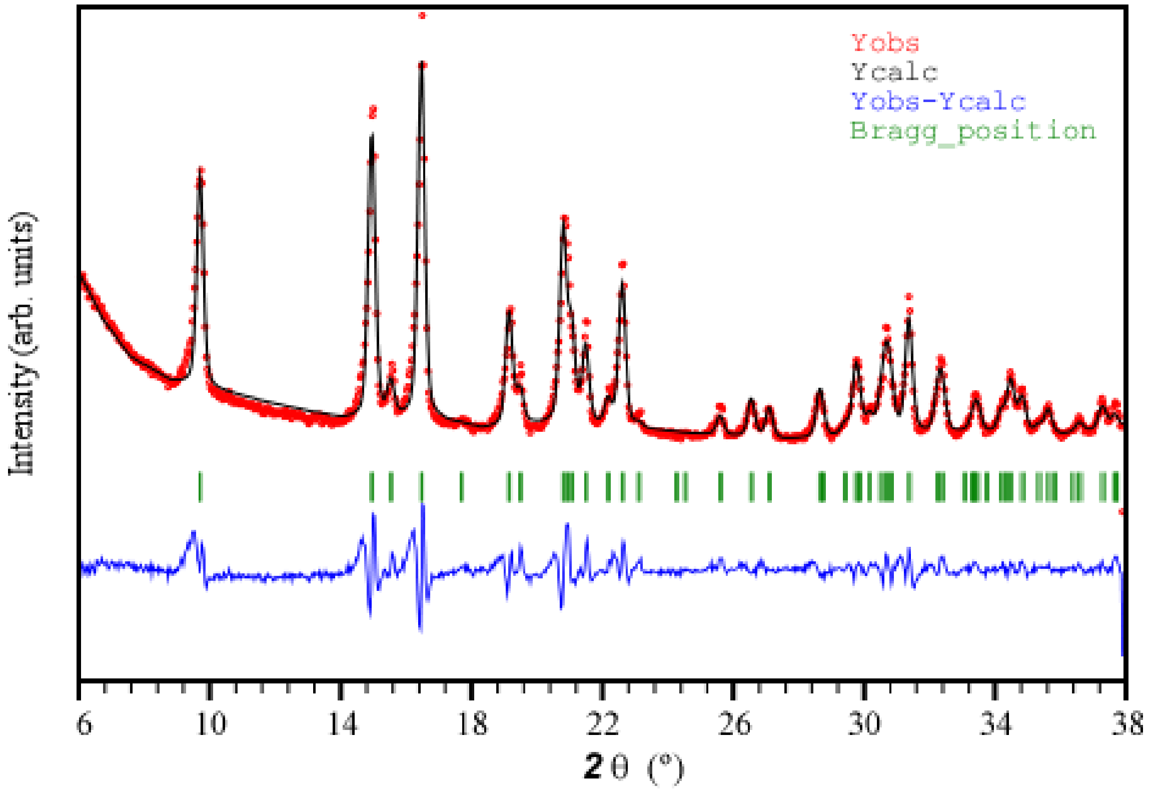

Figure S42. Pattern-matching analysis of the polycrystalline sample of compound 2D_Er-L at 130 °C.

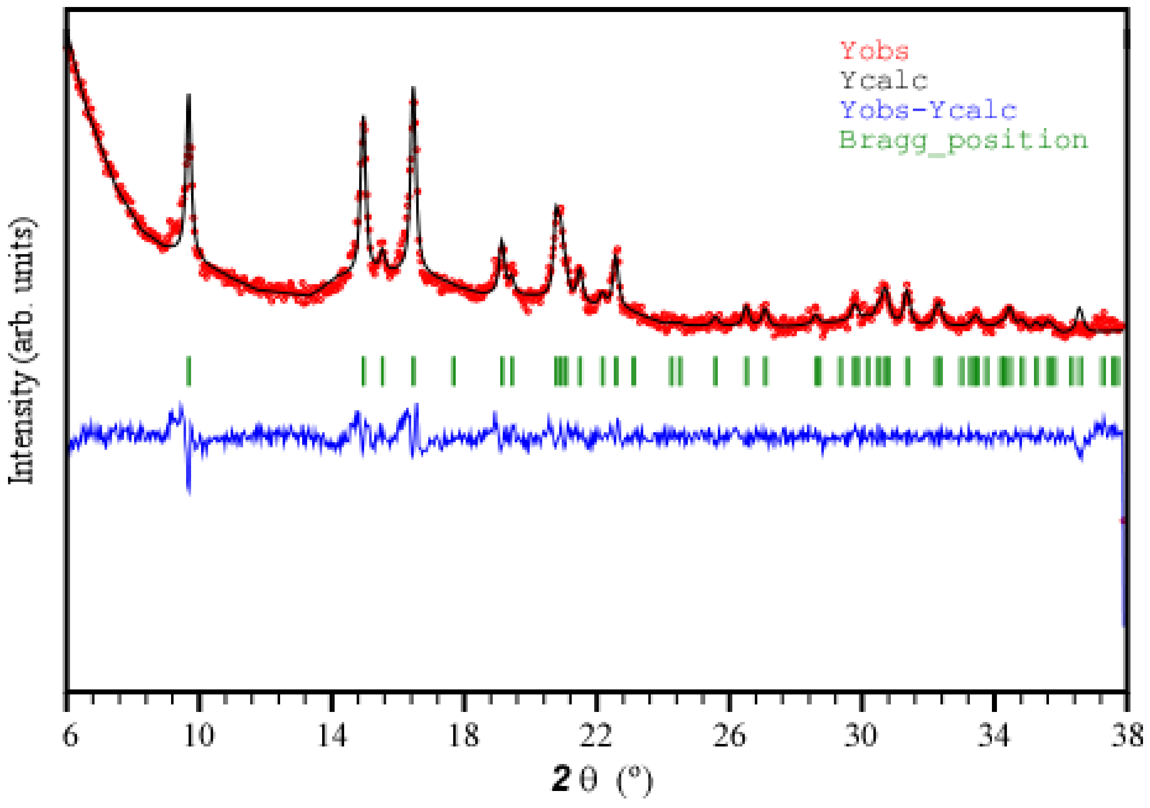

Figure S43. Pattern-matching analysis of the polycrystalline sample of compound 2D_Er-L at 230 °C.

Table S10. Crystallographic data of 2D compounds.

Compounds	Parameters							
	Space group	a (Å)	b (Å)	c (Å)	α (º)	β (º)	γ (º)	V (Å^3)
**2D_Y-L**	*P4₁2₁2*	6.01699	6.01699	36.47774	90	90	90	1320.648
**2D_Y-D**	*P4₃2₁2*	6.01803	6.01803	36.48772	90	90	90	1321.465
**2D_Sm-L**	*P4₁2₁2*	6.08282	6.08282	36.65903	90	90	90	1356.411
**2D_Sm-D**	*P4₃2₁2*	6.08515	6.08515	36.66785	90	90	90	1357.774
**2D_Eu-L**	*P4₁2₁2*	6.06928	6.06928	36.60643	90	90	90	1348.439
**2D_Eu-D**	*P4₃2₁2*	6.07119	6.07119	36.61676	90	90	90	1349.668
**2D_Gd-L**	*P4₁2₁2*	6.05866	6.05866	36.58027	90	90	90	1342.766
**2D_Gd-D**	*P4₃2₁2*	6.05770	6.05770	36.57058	90	90	90	1341.982
**2D_Tb-L**	*P4₁2₁2*	6.04235	6.04235	36.52659	90	90	90	1333.586
**2D_Tb-D**	*P4₃2₁2*	6.04226	6.04226	36.52816	90	90	90	1333.605
**2D_Dy-L**	*P4₁2₁2*	6.02962	6.02962	36.49097	90	90	90	1326.679
**2D_Dy-D**	*P4₃2₁2*	6.02925	6.02925	36.49286	90	90	90	1326.582
**2D_Ho-L**	*P4₁2₁2*	6.01563	6.01563	36.45838	90	90	90	1319.350
**2D_Ho-D**	*P4₃2₁2*	6.01551	6.01551	36.45694	90	90	90	1319.243
**2D_Er-L**	*P4₁2₁2*	6.00398	6.00398	36.42653	90	90	90	1313.094
**2D_Er-D**	*P4₃2₁2*	6.00260	6.00260	36.42982	90	90	90	1312.609
**2D_Tm-L**	*P4₁2₁2*	5.99192	5.99192	36.39906	90	90	90	1306.839
**2D_Tm-D**	*P4₃2₁2*	5.99120	5.99120	36.39936	90	90	90	1306.537
**2D_Yb-L**	*P4₁2₁2*	5.98117	5.98117	36.37256	90	90	90	1301.206
**2D_Yb-D**	*P4₃2₁2*	5.98344	5.98344	36.35849	90	90	90	1301.692
**2D_Er-L-130 ºC**	*P4₁2₁2*	6.00541	6.00541	36.44605	90	90	90	1314.426
**2D_Er-L-230 ºC**	*P4₁2₁2*	6.00518	6.00518	36.53342	90	90	90	1317.477

S14. FT-IR spectroscopy of chiral 3D MOFs.

At high frequencies of FTIR spectrum, all compounds exhibit an intense and broad band between 3600 and 2900 cm^{-1} which corresponds to the stretching vibration of O–H bonds of hydroxyl groups and water molecules. Weak shoulders between 2850 and 2400 cm^{-1} are attributed to C–H stretching vibrations of the tartrate ligand. The intense vibration around 1610 cm^{-1} correspond to the asymmetric stretching of the metal coordinated carboxylate groups, while the symmetric stretching and bending of the same group occur at 1405 cm^{-1}. Bands between 1335 and 1150 cm^{-1} are representative of C–C–H, hydroxyl C–O–H, carboxylate O–C–O, and C–C–O bond bending, followed by two strong and sharp bands at 1135 and 1070 cm^{-1} related to stretching and bending of hydroxyl C–O–H bonds. Different bands at the range of 960-800 cm^{-1} are attributed to trisubstituted C–H

bending vibrations. The vibration bands of the M–O bonds (M = Sm, Eu, Gd) are observed around the range of 605-400 cm^{-1}.

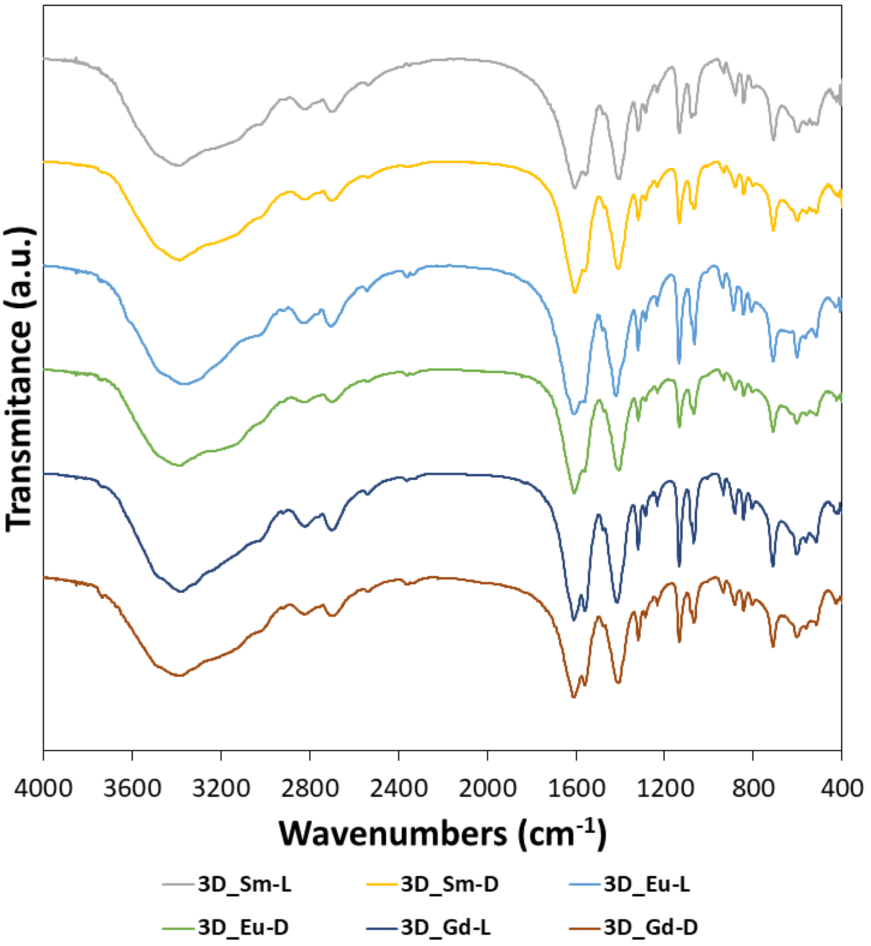

Figure S44. FTIR spectra of all 3D compounds.

S15. FT-IR spectroscopy of chiral 2D MOFs.

As will be detailed below, it is important to note that despite the similarity between the FTIR spectra of the 3D and 2D compounds, there is a notable difference in the bands corresponding to the carboxylic and carboxylate groups sited in the range of 1700-1600 cm^{-1}. At high frequencies of FTIR spectrum, all compounds exhibit an intense and broad band between 3700 and 3100 cm^{-1} which corresponds to the stretching vibration of O–H bonds of hydroxyl and carboxylic groups and coordinated hydroxide and water molecules. Right after, in the range of 3000-2400 cm^{-1}, weak shoulders and peaks attributed to C–H stretching vibrations of the tartrate ligand are observed. Band at 1715 cm^{-1} is related to the asymmetric stretching of the free carboxylic C=O bond, while the same vibrational mode of the metal coordinated carboxylate groups appear at ca. 1600 cm^{-1}. This fact confirms the presence of partially deprotonated hydrogen tartrate ligands. The symmetric stretching and bending of the carboxylic and carboxylate C–O bonds give rise to the same band at 1405 cm^{-1}. Bands between 1320 and 1150 cm^{-1} are representative of C–C–H, carboxylic and hydroxyl C–O–H, carboxylic and carboxylate O–C–O, and C–C–O bond bending, followed by two strong and sharp bands at 1140 and 1070 cm^{-1} related to stretching and bending of hydroxyl C–O–H bonds, although the first band just mentioned is also representative of the carboxylic O–C=O bond bending. Different bands at the range of 950-800 cm^{-1} are attributed to C–C stretching and C–C–H and C–C–C bending vibrations. Finally, vibration bands of the M–O bonds (M = Y, Sm, Eu, Gd, Tb, Dy, Ho, Er, Tm, Yb) are observed around the range of 605-400 cm^{-1}.

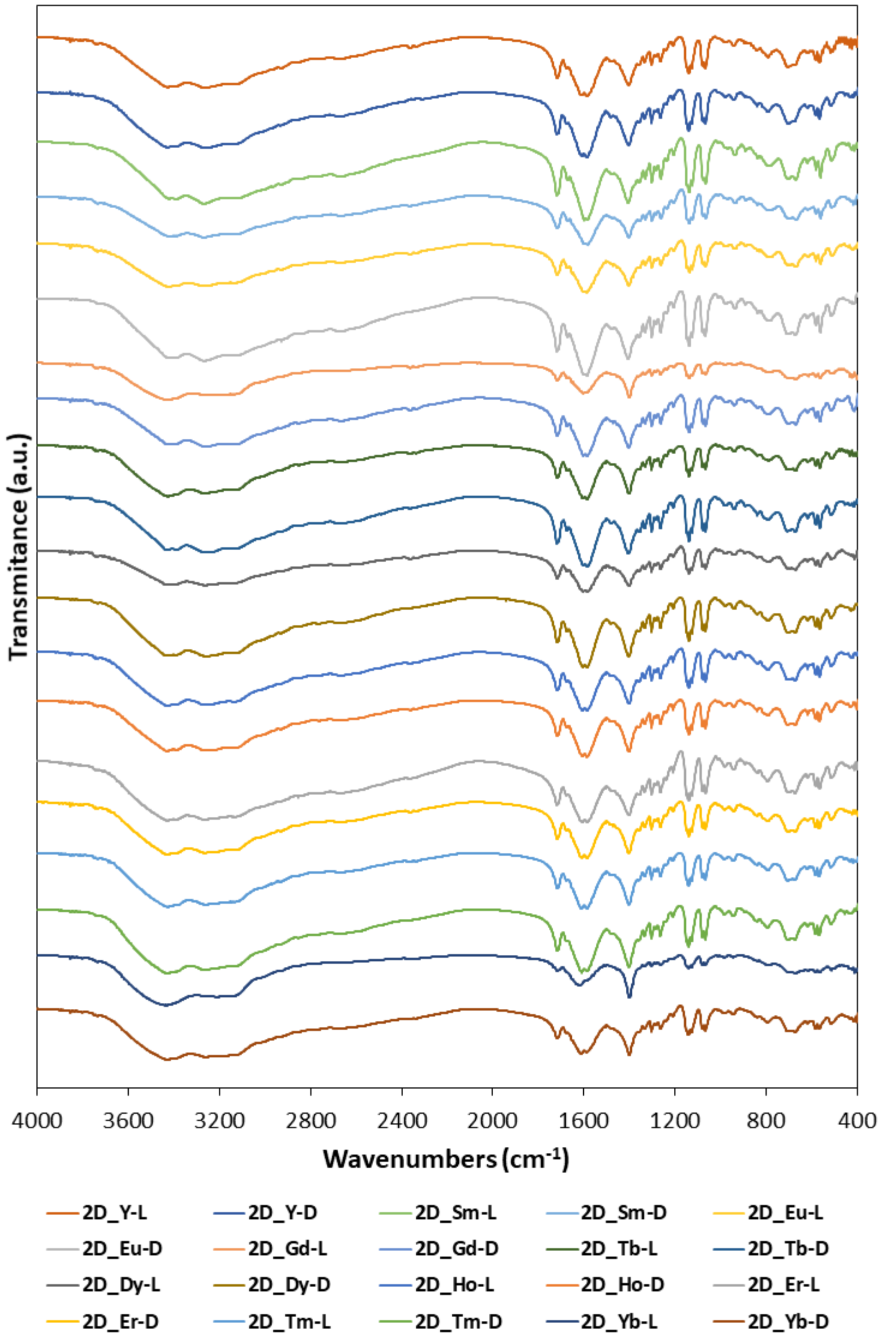

Figure S45. FTIR spectra of all 2D compounds.

S16. Photoluminescence measurements of chiral 3D MOFs.

The following figure shows a comparative view of the visible spectra collected for the europium based enantiomeric 3D MOFs (compounds **3D_Eu-L** and **3D_Eu-D**) under the same experimental conditions to corroborate their equality.

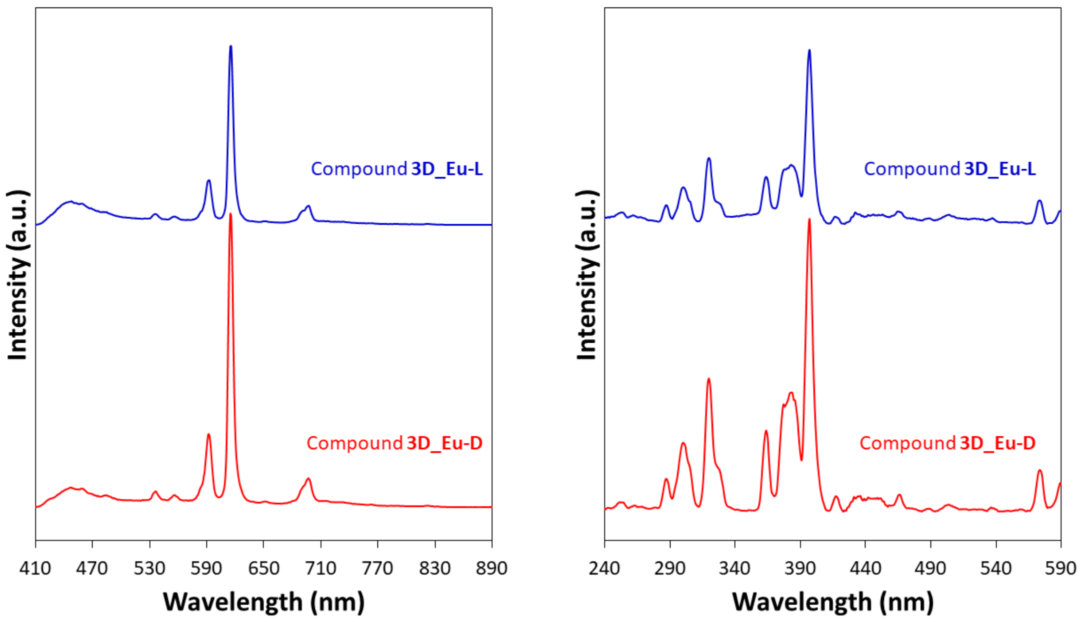

Figure S46. Luminescence properties of Eu-based 3D compounds (3D_Eu-L and 3D_Eu-D) measured at room temperature: (a) Emission spectra under λ_{ex} = 397 nm, and (b) Excitation spectra recorded at the main emission line at 615 nm.

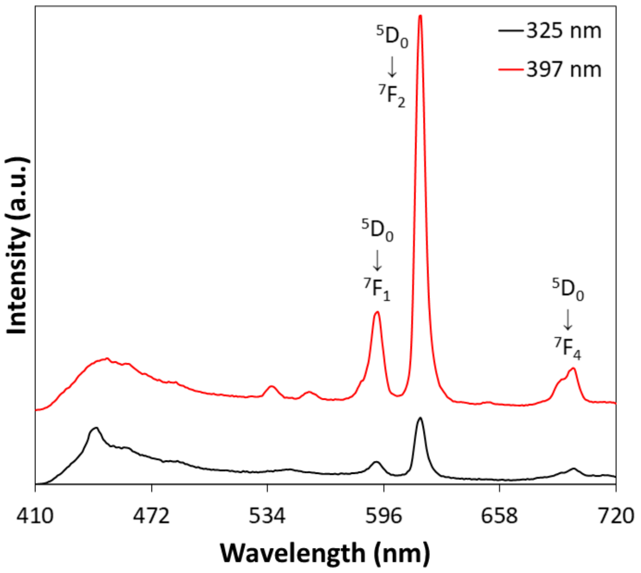

Figure S47. Emission spectra of compound 3D_Eu-L recorded at room temperature.

When compounds **3D_Sm-L** and **3D_Gd-L** are exposed to UV excitation, they only display emission bands in the 400–650 nm range (see Figures S59–60) corresponding to transitions of tartrate ligands. The absence of any ligand-to-samarium energy transfer makes that the emission band does not depend on the excitation wavelength, in such a way that all excitation spectra recorded under different emission lines show almost the same pattern, except for the intensity which changes according to the emission wavelength.

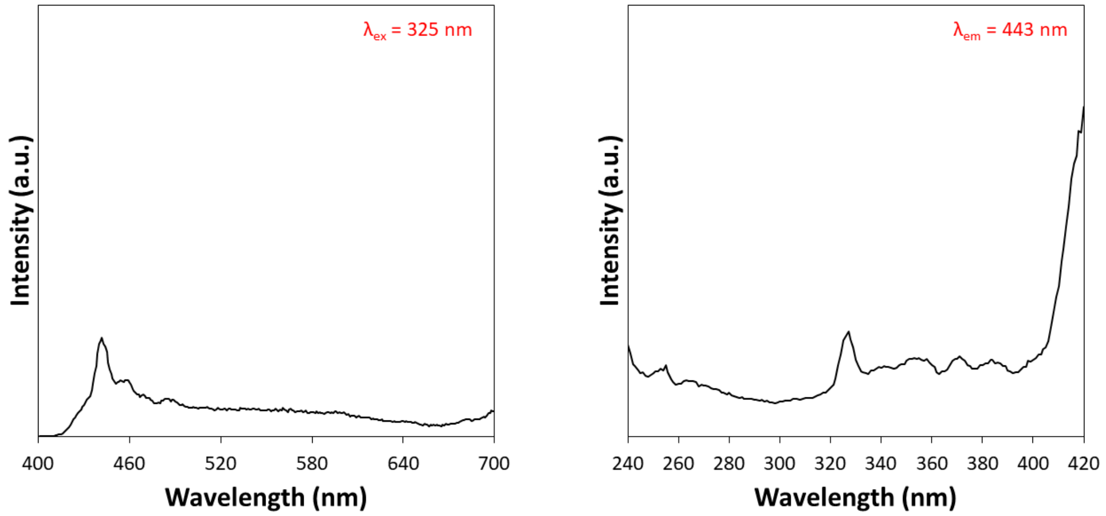

Figure S48. Emission (λ_{ex} = 325 nm) and excitation (λ_{ex} = 443 nm) spectra of 3D_Sm-L.

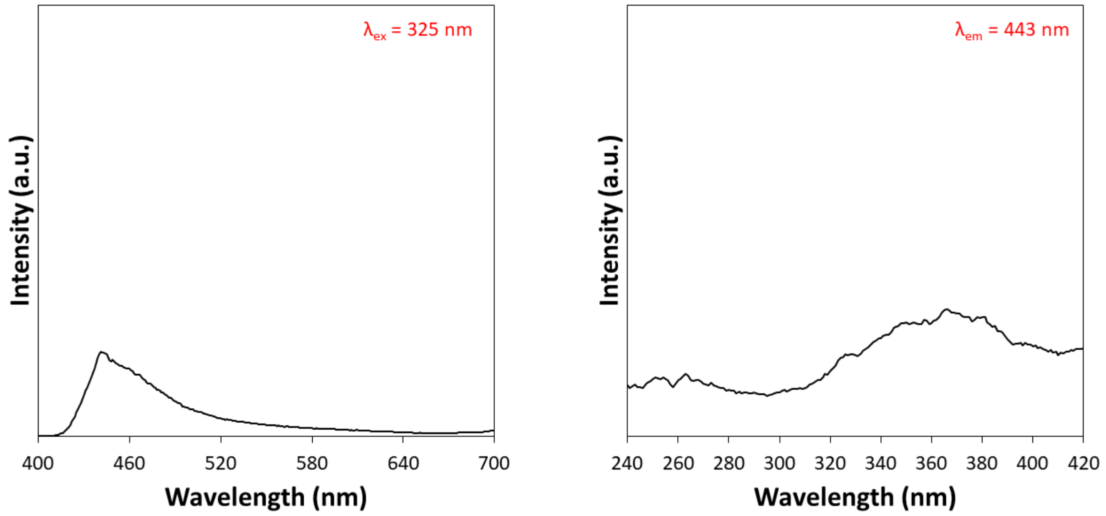

Figure S49. Emission (λ_{ex} = 325 nm) and excitation (λ_{ex} = 443 nm) spectra of 3D_Gd-L.

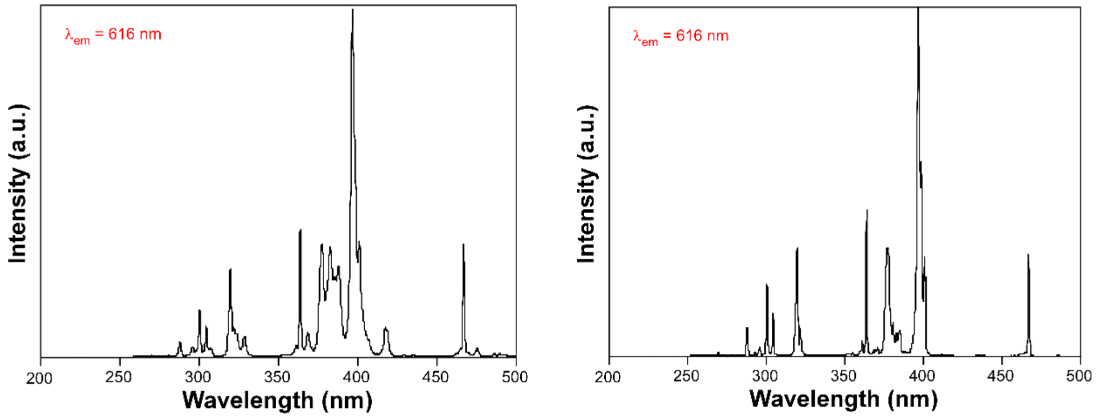

(a) (b)

Figure S50. Excitation spectra recorded at the main emission line at 615 nm for compound 3D_Eu-L (a) at room temperature and (b) 10 K.

S17. Photoluminescence measurements of chiral 2D MOFs.

The following figure shows a comparative view of the visible spectra collected for the europium based enantiomeric 2D MOFs (compounds **2D_Eu-L** and **2D_Eu-D**) under the same experimental conditions to corroborate their equality.

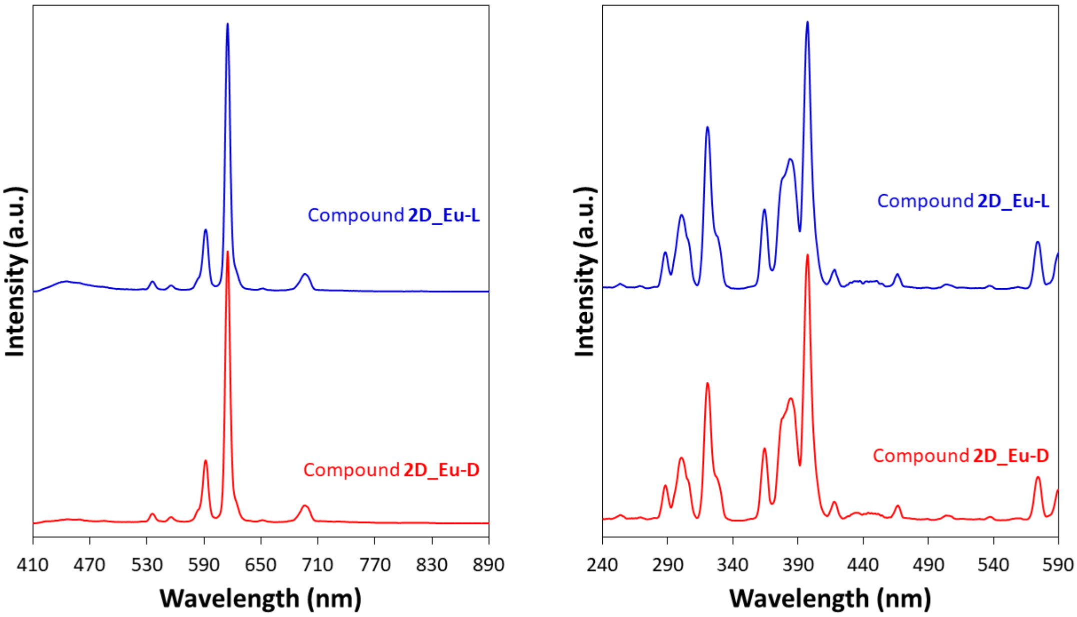

(a) (b)

Figure S51. Luminescent properties of Eu-based 2D compounds (2D_Eu-L and 2D_Eu-D) measured at room temperature: (a) Emission spectra under λ_{ex} = 397 nm, and (b) Excitation spectra recorded at the main emission line at 615 nm.

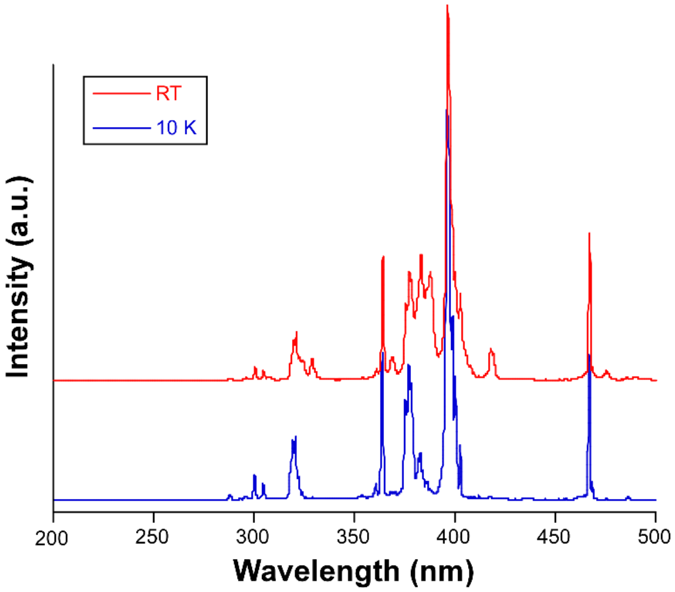

Figure S52. Excitation spectra recorded at the main emission line at 615 nm for compound 2D_Eu-L at room temperature and 10 K.

The luminescence thermometric response was analysed for compound **2D_Eu-L** in view of its interesting temperature dependent luminescence. To that end, a ratiometric analysis between the ligand's fluorescent ($\lambda_{em,max}$ = 430 nm) and main Eu-centred (the hypersensitive $^7F_2 \leftarrow {}^5D_0$ transition centred at 616 nm) bands, from which Δ parameter was calculated. From the latter, the relative sensitivity (S_r) was estimated for the whole temperature range. As observed in Figure S53, the maximum S_r is observed at 50 K (1.42 %K^{-1}).

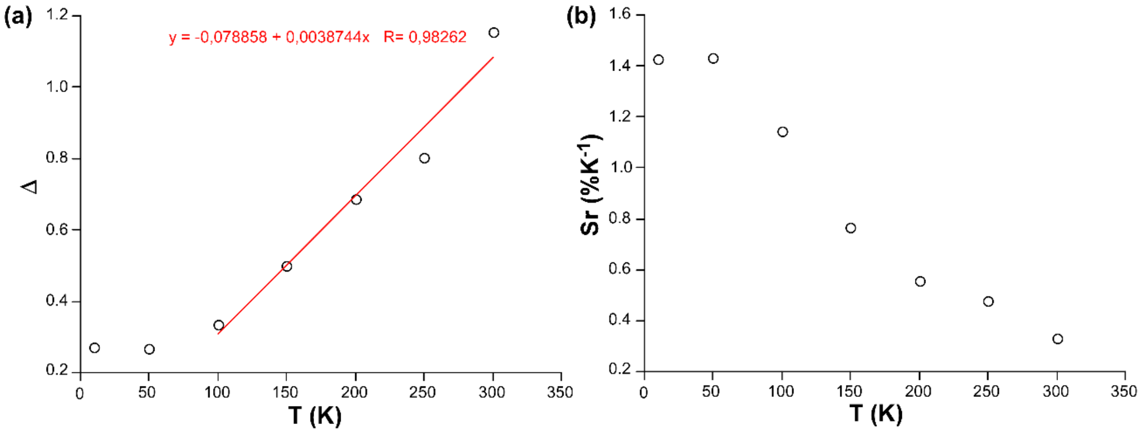

Figure S53. Thermoluminescent data for compound 2D_Eu-L in the form of: (a) Δ vs T plot showing the best fit for the linear high temperature range and (b) relative sensitivity vs T plot.

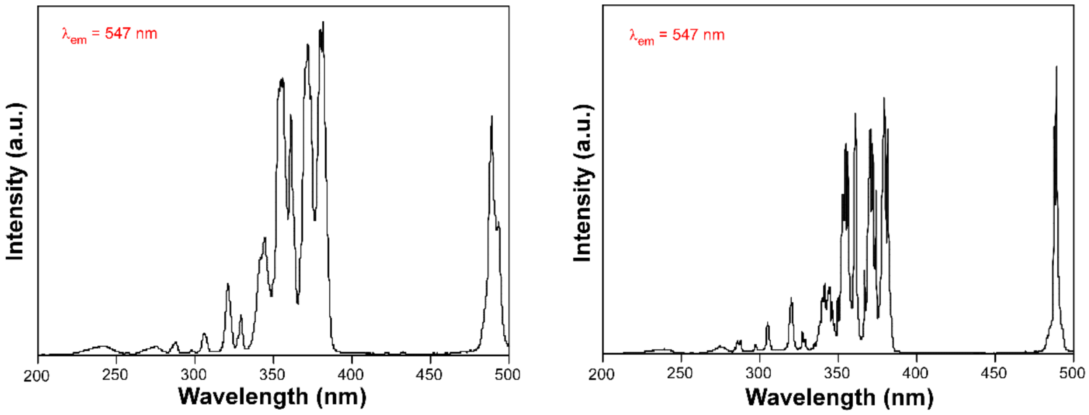

(a) **(b)**

Figure S54. Excitation spectra recorded at the main emission line at 545 nm for compound 2D_Tb-L (a) at room temperature and (b) 10 K.

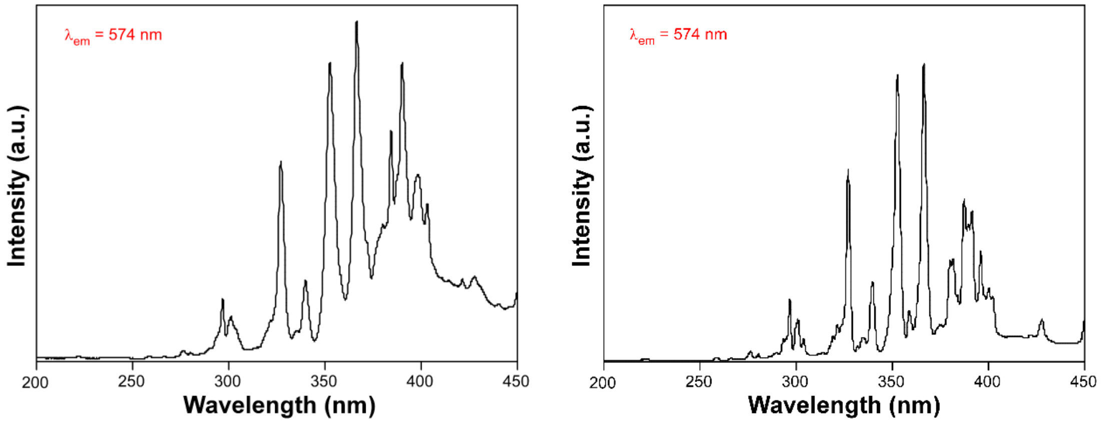

(a) (b)

Figure S55. Excitation spectra recorded at the main emission line at 573 nm for compound 2D_Dy-L (a) at room temperature and (b) 10 K.

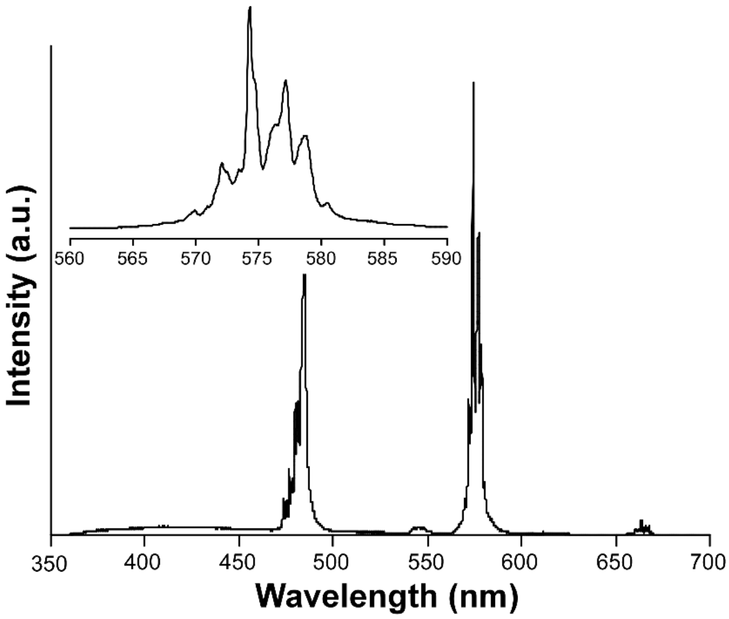

Figure S56. Emission spectrum of compound 2D_Dy-L recorded at 325 nm light at 10 K.

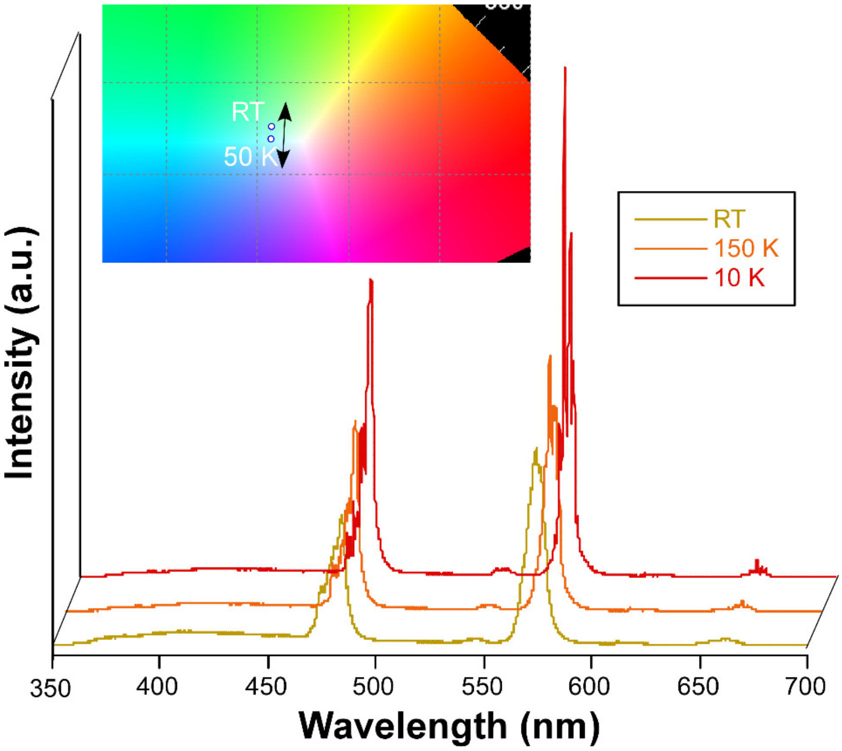

Figure S57. Emission spectrum of compound 2D_Dy-L recorded at 325 nm light at variable temperature. Inset shows the small variation of the emitted colour.

When compounds **2D_Y-L, 2D_Sm-L, 2D_Gd-L, 2D_Ho-L, 2D_Er-L, 2D_Tm-L** and **3D_Yb-L** are exposed to UV excitation, they only display emission bands in the 400–650 nm range (see Figures S57–63) corresponding to transitions of tartrate ligands. The whole emission band corresponds to a unique excitation pathway given that all excitation spectra recorded under different emission lines show almost the same pattern, except for the intensity which changes according to the emission wavelength.

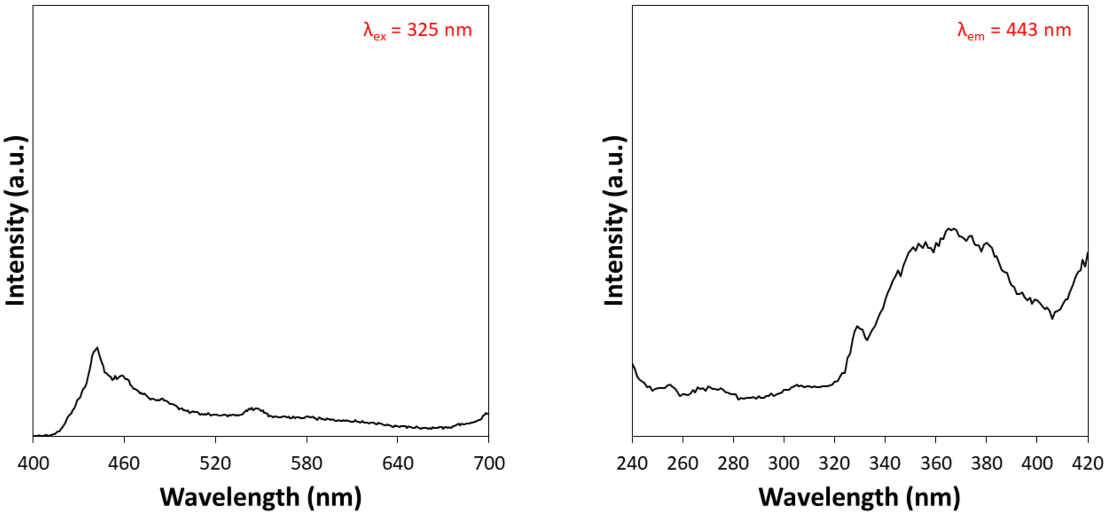

Figure S58. Emission (λ_{ex} = 325 nm) and excitation (λ_{ex} = 443 nm) spectra of 2D_Y-L.

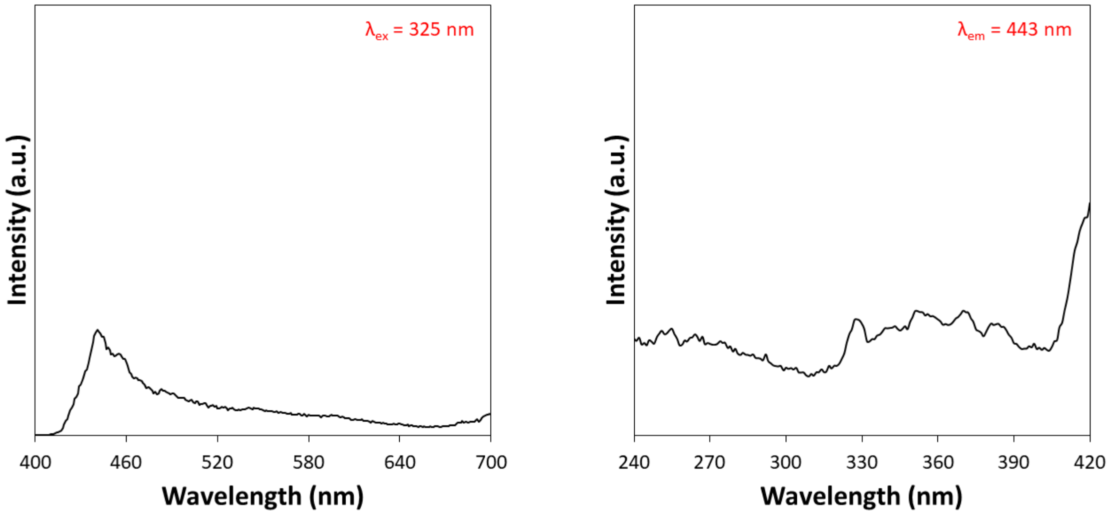

Figure S59. Emission (λ_{ex} = 325 nm) and excitation (λ_{ex} = 443 nm) spectra of 2D_Sm-L.

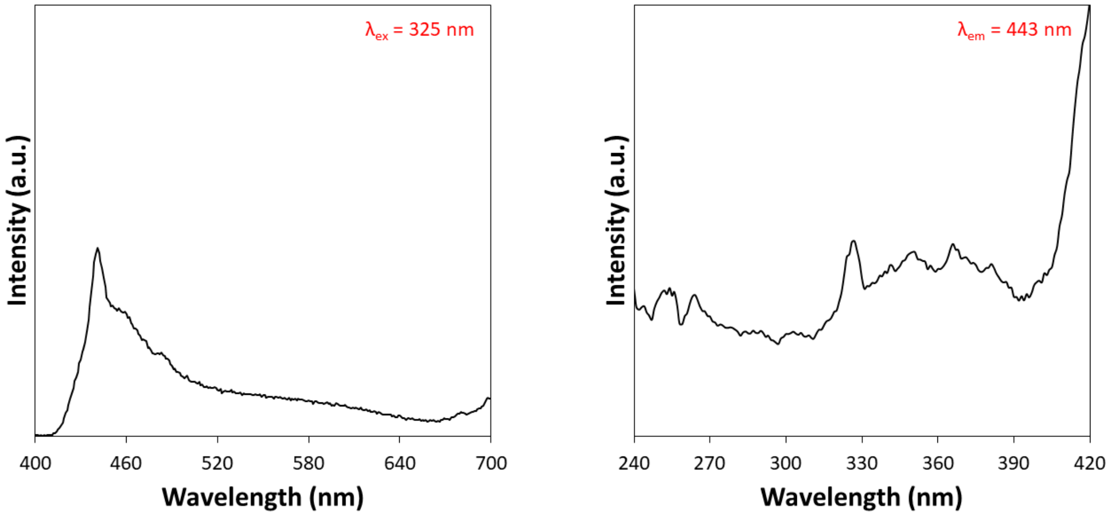

Figure S60. Emission (λ_{ex} = 325 nm) and excitation (λ_{ex} = 443 nm) spectra of 2D_Gd-L.

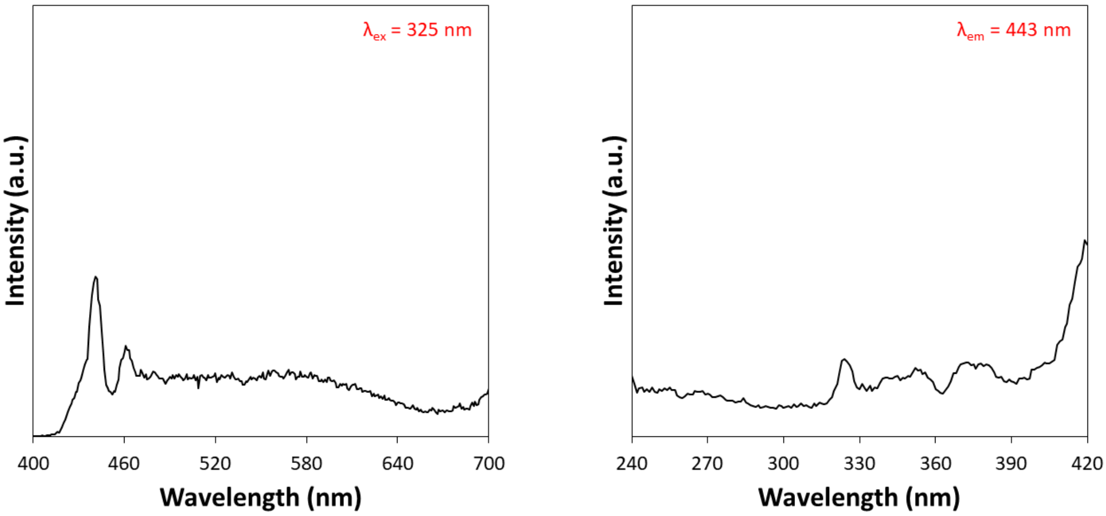

Figure S61. Emission (λ_{ex} = 325 nm) and excitation (λ_{ex} = 443 nm) spectra of 2D_Ho-L.

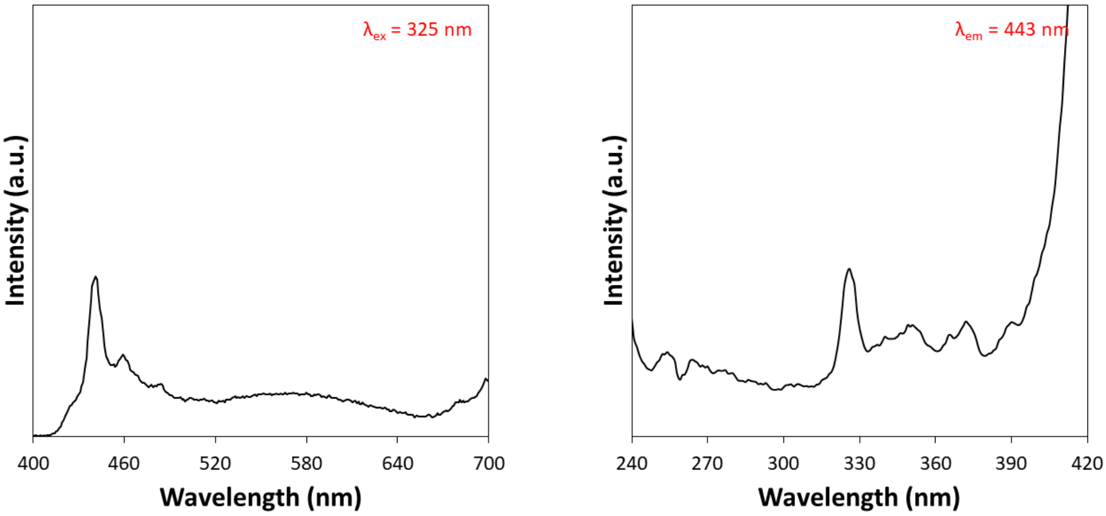

Figure S62. Emission (λ_{ex} = 325 nm) and excitation (λ_{ex} = 443 nm) spectra of 2D_Er-L.

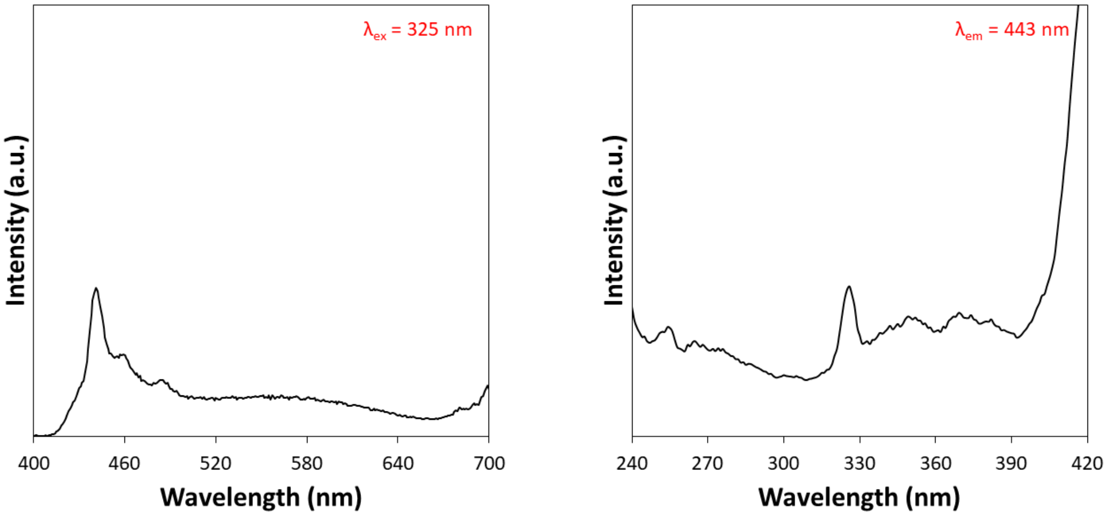

Figure S63. Emission (λ_{ex} = 325 nm) and excitation (λ_{ex} = 443 nm) spectra of 2D_Tm-L.

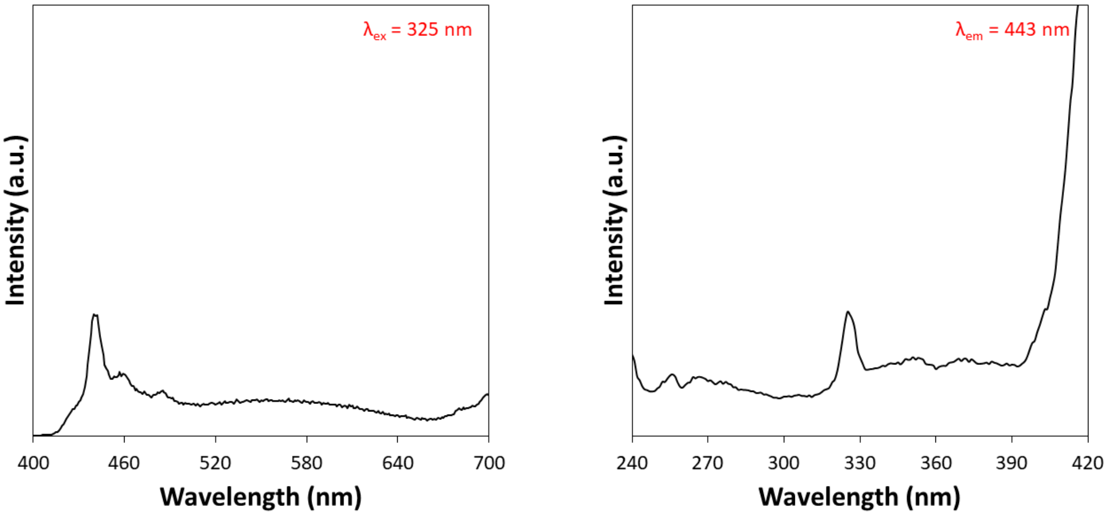

Figure S64. Emission (λ_{ex} = 325 nm) and excitation (λ_{ex} = 443 nm) spectra of 2D_Yb-L.

S18. Comparative luminescence of Eu(III)-based compounds.

To start with the comparative analysis of the luminescence of these compounds **2D_Eu-L** and **3D_Eu-L**, the following figure shows their emission spectra measured under the same experimental conditions.

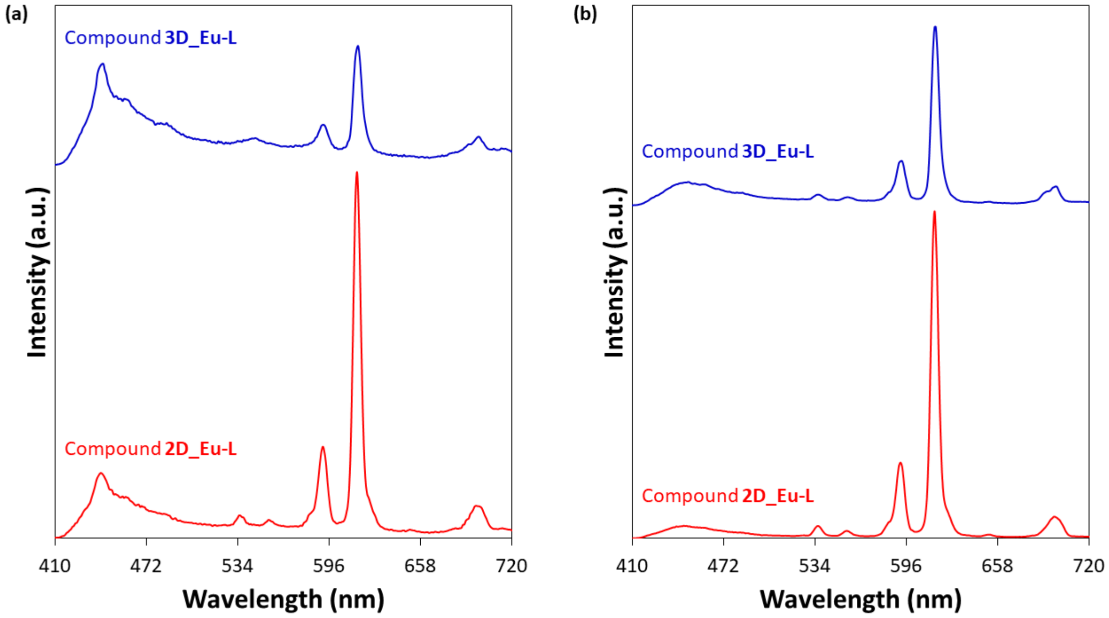

Figure S65. Emission spectra for 2D_Eu-L and 3D_Eu-L at room temperature with (a) λ_{ex} = 325 nm and (b) λ_{ex} = 397 nm.

S19. Lifetime measurements.

The emission lifetimes for **2D_Eu-L** were estimated from the fitting of the mono-exponential decay curves at RT and 10 K.

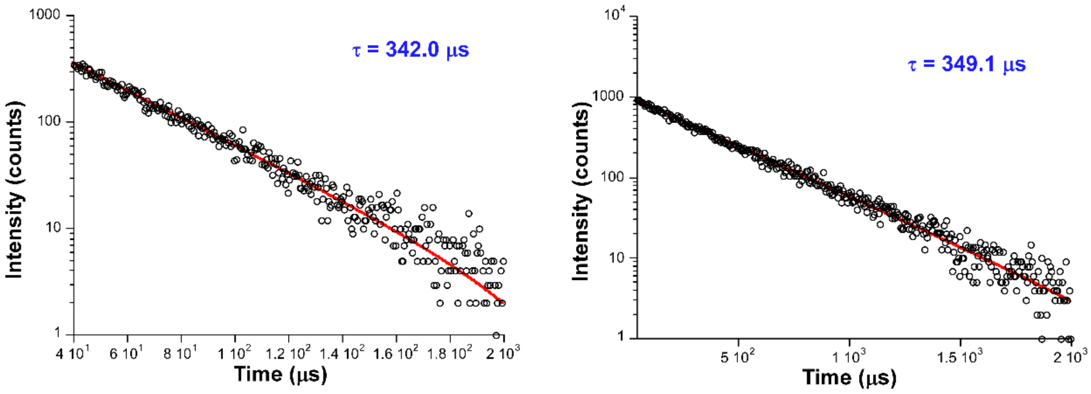

(a) **(b)**

Figure S66. Decay curves recorded for 2D_Eu-L at (a) room temperature and (b) 10 K with λ_{ex} = 325 nm and λ_{em} = 616 nm.

The ligand emission corresponds to short fluorescence in **2D_Eu-L** and could only be measured at low temperature owing to the low emission intensity, owing to the energy transfer occurred to the Eu(III) ion. In order to obtain a reliable fitting, the data had to be deconvoluted using the reference signal recorded at the emission wavelength of the LED source (by reflection) as shown in the following figure.

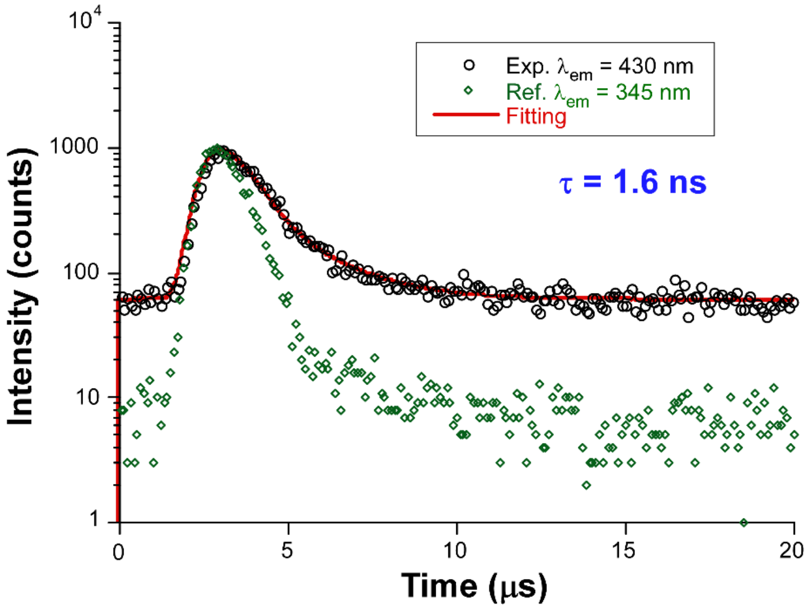

Figure S67. Decay curve recorded for 2D_Eu-L to estimate the lifetime of the ligand's fluorescence (10 K, λ_{ex} = 345 nm and λ_{em} = 430 nm).

The lifetime was shown to be lengthened for **3D_Eu-L** both at RT and 10 K.

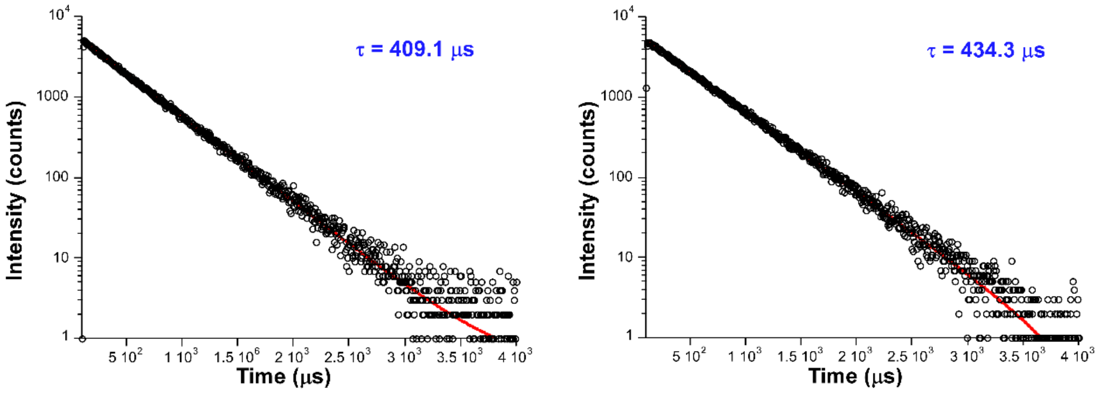

(a) **(b)**

Figure S68. Decay curves recorded for 3D_Eu-L at (a) room temperature and (b) 10 K with λ_{ex} = 325 nm and λ_{em} = 616 nm.

Lifetimes of compound **2D_Tb-L** were achieved in the same way.

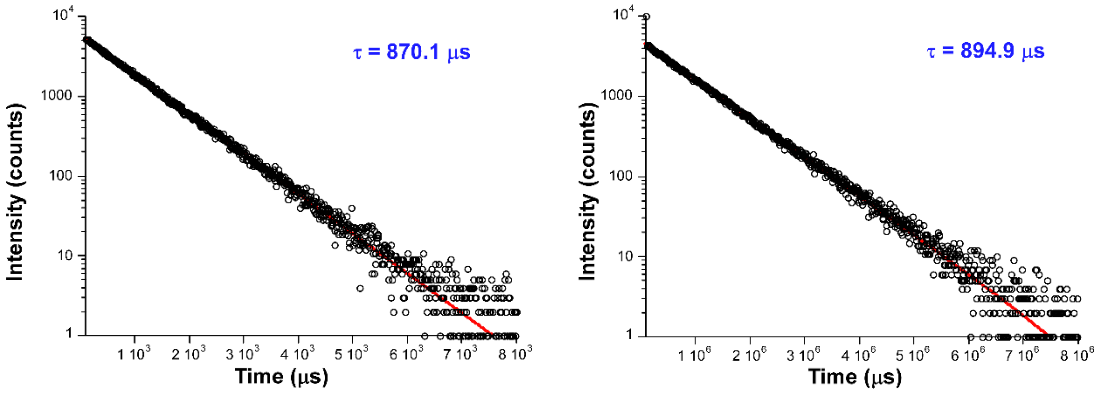

(a) **(b)**

Figure S69. Decay curves recorded for 2D_Tb-L at (a) room temperature and (b) 10 K with λ_{ex} = 325 nm and λ_{em} = 544 nm.

In the case of **2D_Dy-L**, both the lifetime associated with the ligand and Dy(III) centre were recorded using the same experimental setup.

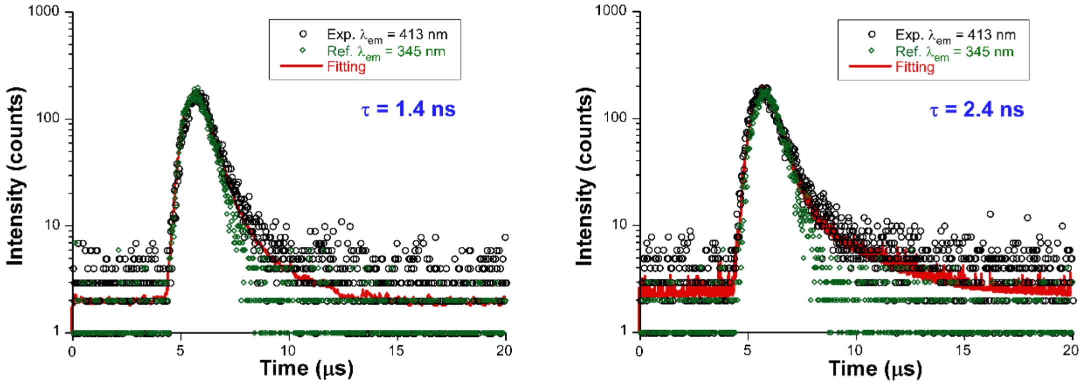

(a) **(b)**

Figure S70. Decay curves recorded for 2D_Dy-L to estimate the lifetime of the ligand's fluorescence (λ_{ex} = 345 nm and λ_{em} = 413 nm) at (a) RT and (b) 10 K.

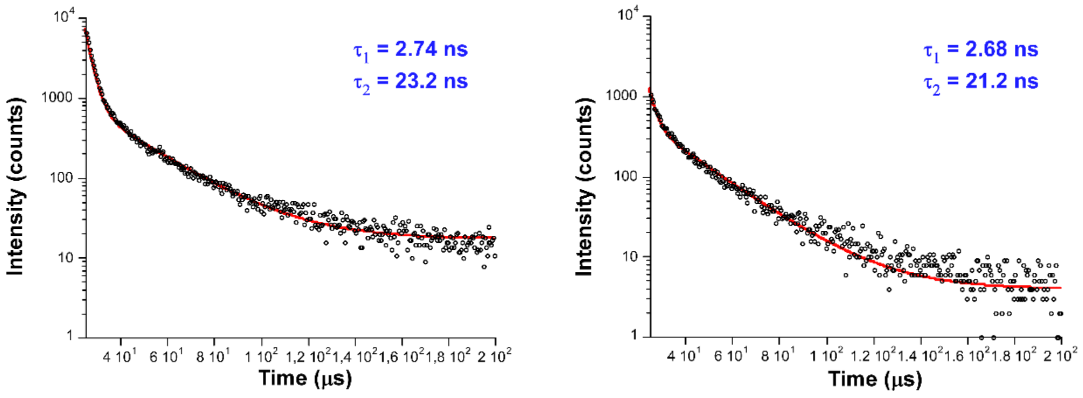

(a) **(b)**

Figure S71. Decay curves recorded for 2D_Dy-L at (a) room temperature and (b) 10 K with λ_{ex} = 325 nm and λ_{em} = 574 nm.

S20. Circular Dichroism and Circularly Polarized Luminescence measurements.

Samples for CD measurements were prepared as follows: a precision weighted amount of material (2 mg) was suspended in Milli-Q H_2O (4 mL) and sonicated over 2 h.

In emission and CPL solid sample measurements, 5 mg of each sample were suspended Mili-Q H_2O (4 mL) and sonicated during 1 hour. The obtained suspension was used directly for fluorescence and CPL measurements. They were performed in an Olis DSM172 spectrophotometer with a 90º angle system, using a 1.0 cm path-length quartz cell and a fixed wavelength LED (370 nm) as the excitation source. Unfortunately, the only samples which showed total fluorescence signals were **2D_Tb-L** and **2D_Tb-D** (Figure S71), but they did not have enough intensity to get a reliable CPL signal. In addition, a scattered signal from the excitation source lead to a background signal, thus preventing even a good emission spectra under the above mentioned conditions. Although undesirable scattering interferences can be avoided in part under 180º angle geometry in a JASCO CPL-300 spectrophotometer we only observed very weak fluorescence intensities under a (150 W) Xe lamp as excitation source.

.

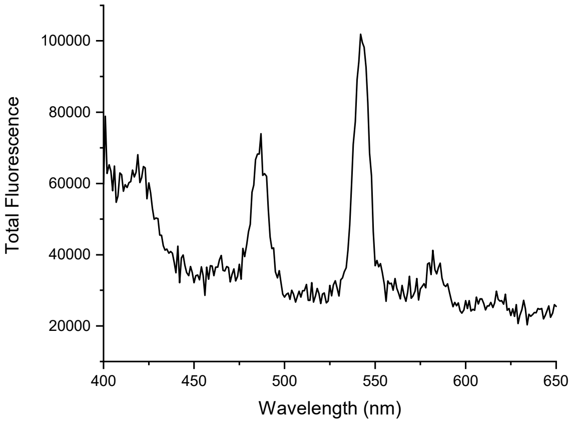

Figure S72. Total fluorescence spectra of 2D_Tb-L and 2D_Tb-D in aqueous suspensions.

Finally, measurements of **2D_Eu** and **2D_Tb** samples were possible using the corresponding dispersions in potassium bromide pills. 4 mg of sample in each case were grinded together with 225 mg of KBr until getting a homogeneous powder. It was pressed

with a mechanical press Specac and Evacuable Pellet Die 2I-03000-10 in high vacuum conditions, obtaining a transparent pill of 13 mm diameter.

To excitate **2D_Eu** samples, a fixed wavelength at 397 nm was used, while 374 nm was selected as excitation wavelength to **2D_Tb** samples. Solid sample CPL measurements are prone to generate artefacts. For that reason, measurements were carried out rotating the sample and the signal is the average of all of orientations. Thus, CPL spectra were collected by accumulating 80 scans: 10 scans in each direction and rotating 45° until completing the turn. In all the cases a 0.5 seg of integration time were selected.

All of samples showed good emission signals, but ΔI of both **2D_Tb** enantiomers was found in the detection limit of our equipment, and consequently g_{lum} value is not reliable (Figures S72 and S73). On the other hand, front and back side signals were measured and compared. In each case, both sides showed similar signals, demonstrating again that there is no interference due to the macroscopic anisotropies of the sample (Figures S74-S77).

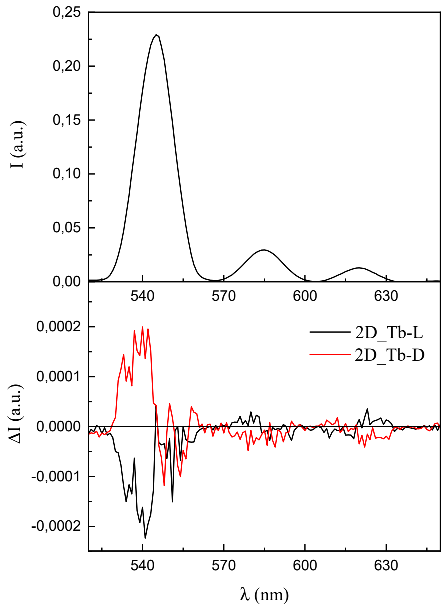

Figure S73. Total fluorescence and ΔI spectra of 2D_Tb-L and 2D_Tb-D in KBr pills.

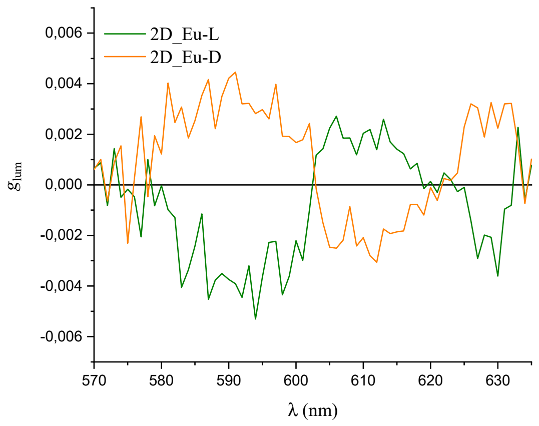

Figure S74. g_{lum} values of 2D_Eu dispersed in KBr pills.

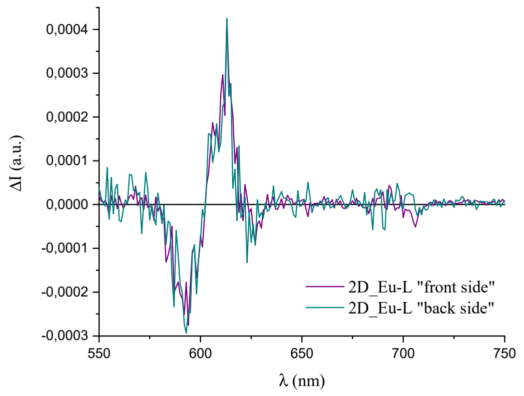

Figure S75. ΔI spectra of 2D_Eu-L in KBr pills measuring both front and back side.

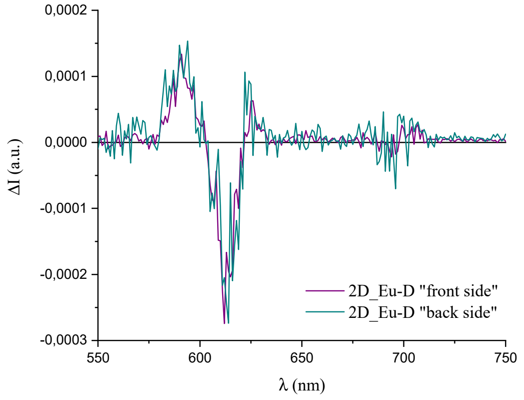

Figure S76. ΔI spectra of 2D_Eu-D in KBr pills measuring both front and back side.

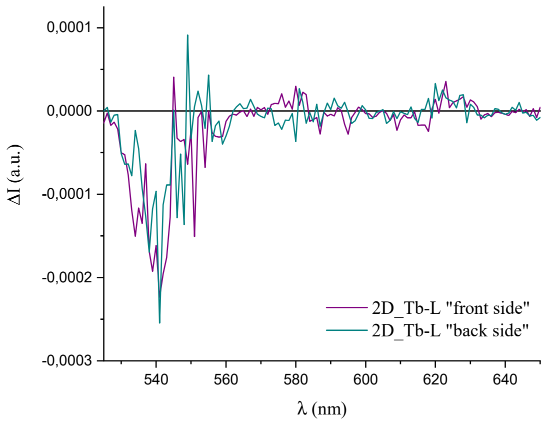

Figure S77. ΔI spectra of 2D_Tb-L in KBr pills measuring both front and back side.

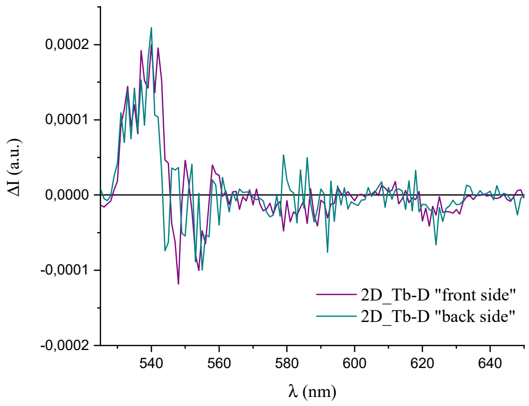

Figure S78. ΔI spectra of 2D_Tb-D in KBr pills measuring both front and back side.

S21. UV-Vis measurements.

Samples of CD were reused to UV-Vis measurements.

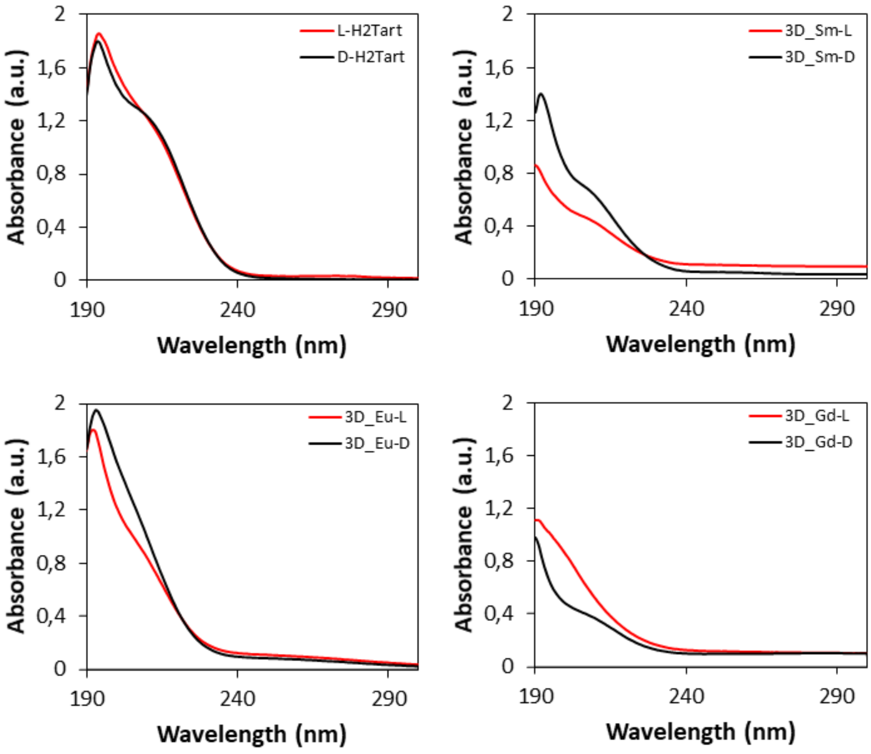

Figure S79. UV-Vis spectra of L- and D-tartaric acid and all 3D compounds.

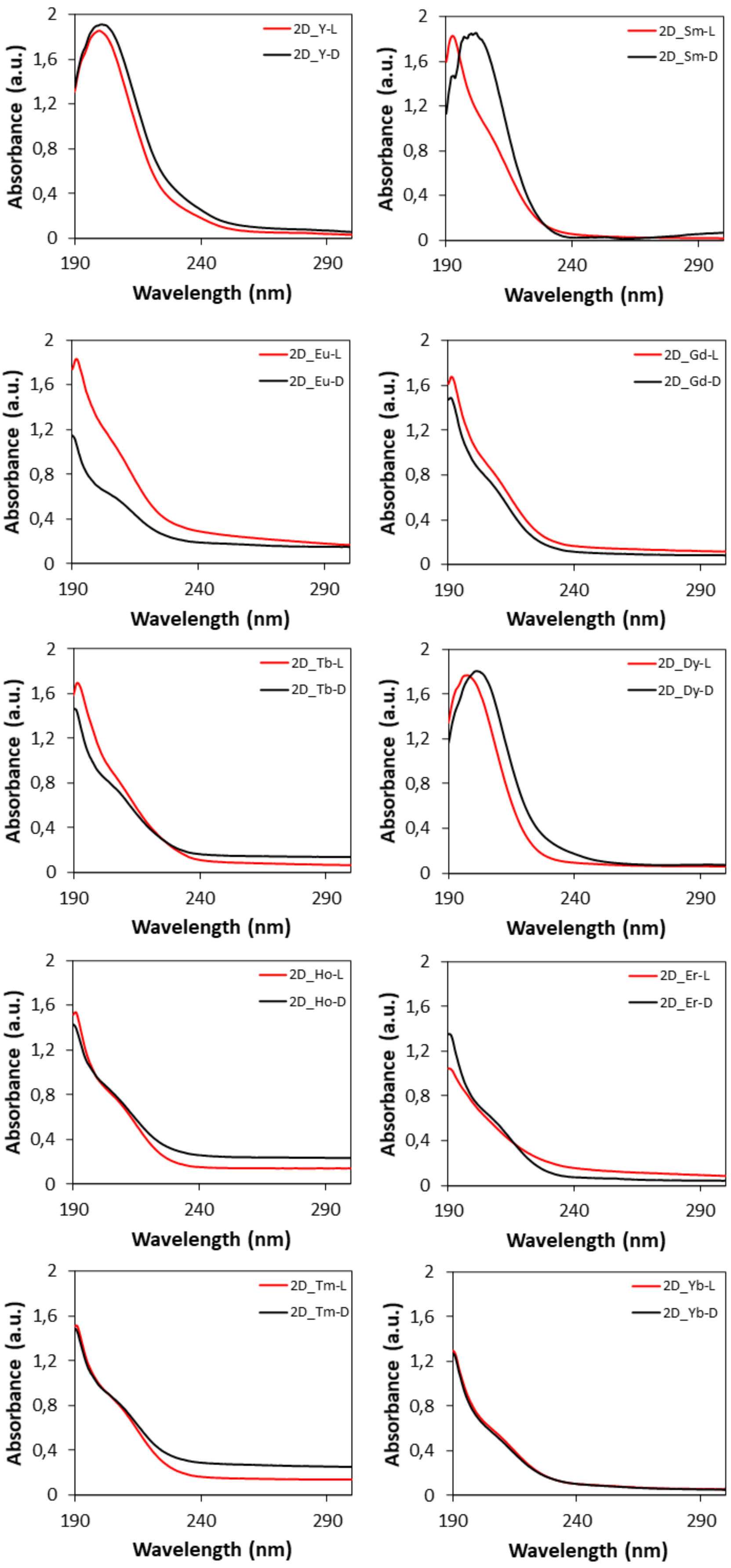

Figure S80. UV-Vis spectra of all 2D compounds.

References

1. CrysAlisPro Software System, Agilent Technologies UK Ltd, Oxford, UK, **2019**.
2. Bruker Apex2, Bruker AXS Inc., Madison, Wisconsin, USA, **2004**.
3. G. M. Sheldrick. SADABS, Program for Empirical Adsorption Correction, Institute for Inorganic Chemistry, University of Göttingen, Göttingen, Germany, **1996**.
4. Altomare, M. C. Burla, M. Camilla, G. L. Cascarano, C. Giacovazzo, A. Guagliardi, A. G. G. Moliterni, G. Polidori and R. Spagna. SIR97: a new tool for crystal structure determination and refinement. J. Appl. Crystallogr., **1999**, *32*, 115-119.
5. G. M. Sheldrick. SHELX-2014, Program for Crystal Structure Refinement, University of Göttingen, Göttingen, Germany, 2014. (b) L. J. Farrugia. WinGX suite for small-molecule single-crystal crystallography. J. Appl. Cryst., **1999**, *32*, 837-838.
6. J. Rodriguez-Carvajal. Recent advances in magnetic structure determination by neutron powder diffraction. Physica B., **1993**, *192*, 55-69.
